# PROTOCOL: Mega-Map of Systematic Reviews and Evidence and Gap Maps on Interventions and Programs for Age-Friendly Environments

**DOI:** 10.1177/18911803261437804

**Published:** 2026-07-15

**Authors:** Amanda Fernandes, Ruvimbo Nhandara, Louise Lafortune, Thomas Katairo, Promise Nduku, Thiago Herick de Sá

**Affiliations:** 1 3489 Health Determinants, Promotion and Prevention Department, World Health Organization, Geneva, Switzerland; 2 Pan-African Collective for Evidence, Johannesburg, South Africa; 3 3710 McMaster Institute for Research on Aging, Hamilton, Ontario, Canada

## Abstract

This is the protocol for a Campbell mega-map. This mega-map has two main objectives: 1. Identify, map, and describe existing evidence from systematic reviews and evidence and gap maps on interventions and programs aimed at creating and sustaining age-friendly environments. 2. Identify existing gaps in methods, interventions, outcomes, and geographies to assist researchers and policymakers in making evidence-informed research and funding decisions.

## Background

### The Problem, Condition, or Issue

Population aging is a global reality. As we achieve longer life expectancy, a longer healthy life is not yet a reality for everyone. Population aging is a major demographic shift experienced by countries worldwide, with low- and middle-income countries facing rapid changes and less time to adapt (WHO, 2024b). By 2030, one in six people globally will be aged 60 years or older, with the total number reaching 1.4 billion (and 2.1 billion by 2050). The majority of these individuals will live in low- and middle-income countries (WHO, 2025c).

The demographic shift toward an aging population affects every sector of society and requires collective, sustainable action. Worldwide, countries are facing significant difficulties in adapting their health and social systems to respond effectively to this unprecedented change. Furthermore, rapid urbanization and migration add complexity to the global challenge to provide longer, healthier lives for all (United Nations Department of Economic and Social Affairs, 2024).

The physical, social, and policy environments are critical determinants of health and wellbeing throughout the aging process. To maximize opportunities for healthy aging, it is essential to create supportive environments that foster individual’s ability to pursue what they value, irrespective of age (WHO, 2015b, 2021a). Age-friendly environments (such as in the home, community) foster healthy aging by building and maintaining intrinsic capacity across the life course and enabling greater functional ability in someone with a given level of capacity (WHO, 2007). Notably, the creation of age-friendly environments is one of the four action areas of the United Nations Decade of Healthy Ageing (WHO, 2021a). Developing age-friendly cities and communities (AFCC) has become a widely adopted strategy for promoting age-friendly environments, centring on AFCC interventions across eight domains of action: housing, transportation, outdoor spaces and buildings, community support and health services, communication and information, social participation, respect and social inclusion, and civic participation and employment as outlined in the WHO AFCC framework (WHO, 2007, 2023).

WHO’s Global Network for Age-friendly Cities and Communities (GNAFCC) has over 1,700 members in more than 50 countries as of October 2025 (WHO, 2025a). Their local AFCC programs follow a well-established cycle to improve age-friendliness, starting from engaging people, sectors, and stakeholders to understand needs and opportunities, then moving on to strategic planning and implementing interventions, and finally evaluating the process and outcomes to learn lessons and guide the development of the next cycle. In parallel, the development of age-friendly environments can be accelerated through subnational and national AFCC programs (WHO, 2023). Through their activities and support for local AFCC programs in both rural and urban areas, these programs help ensure that more people can age with health and well‑being. National and subnational AFCC programs also enable countries to fulfill global commitments, such as those of the United Nations Decade of Healthy Ageing (2021–2030) action plan, which calls for national guidance on fostering healthy aging in cities and communities (WHO, 2021a). These initiatives also serve as inspiration for other countries to develop their own AFCC ecosystem.

The spread and diversity of interventions across the eight domains of action can be observed within GNAFCC with a growing number of cities and communities committing to becoming more age-friendly (WHO, 2023, 2025a). Concomitantly, we observe a growing number of studies on the impacts of age-friendly environments interventions and programs (Hérick de Sá et al., 2024; Hong et al., 2023; Montayre et al., 2022; Sánchez-González et al., 2020), which is essential to ensure that actions prioritized and deployed by AFCC are informed by evidence. Although AFCC programs continue to expand, stakeholders still encounter significant challenges in effectively drawing on the existing evidence base. This difficulty stems in part from the absence of a comprehensive, consolidated map of available evidence to guide policy and implementation decisions. Equally lacking is a systematic and detailed mapping of evidence gaps that could help establish research priorities and direct future evaluation efforts. Enhancing the capacity to use existing evidence efficiently—while rigorously identifying and addressing areas where knowledge remains limited— has been recognized by GNAFCC as essential to enhancing both the network’s capacity and the overall impact of AFCC programs (WHO, 2018, 2023).

To enhance the evidence base for age-friendly environments, this mega-map aims to identify, describe, and provide a visual, interactive display of the evidence on (i) interventions designed to create and sustain age-friendly environments across each of the eight domains of the WHO framework, and (ii) AFCC programs at local, subnational, or national levels.

### The Intervention

There are several different types of interventions and programs that create and sustain age-friendly environments. In 2007, the WHO launched the “Global Age-Friendly Cities: A Guide” as part of the global movement for AFCC and its accompanying Checklist of Essential Features of Age-Friendly Cities (WHO, 2007). Developed through collaboration with older people, their communities and research conducted in multiple cities across different countries, the guide provides a foundational framework for prioritizing actions and engaging stakeholders in the creation of AFCC. The guide identifies eight domains of action (WHO, 2007):

(1) Housing
(2) Transportation
(3) Outdoor spaces and buildings
(4) Community support and health services
(5) Communication and information
(6) Social participation
(7) Respect and social inclusion
(8) Civic participation and employment

Interventions can be implemented across one or more domains at different levels, such as one-on-one or group-focused, as well as at community (e.g., any geographically, physically, digitally, or culturally defined environments such as public community spaces, workplaces, and educational settings), local (e.g., the level below the sub-national government, often at city, metropolitan level, villages, and townships), regional/subnational (e.g., the level below the national government, often at the regional, provincial or state level), and national levels (e.g., usually at government- or public authority-led initiatives undertaken at the country level) (WHO, 2007, 2015a, 2018, 2023, 2025b). In mapping the body of available evidence, we set operational definitions to describe the scope and focus of each of the domains of action (WHO, 2007, WHO, 2015a). In addition to the domain-specific interventions, we added AFCC programs as the ninth intervention category to capture evidence on programs in their entirety, as opposed to individual components. Table 1 lists and provides operational definitions for each intervention category.

**Table 1:** Categories of Interventions: Operational definitions and examples

Domain of action	Operational definition - describes the scope and focus of the action, intervention, program or initiative
Housing	The housing domain refers to the physical and social aspects of housing that impact the well-being and quality of life of older people. This includes ensuring that housing options are safe, affordable, accessible, and suitable for the diverse and changing needs and preferences of older people. Housing design, location, affordability, and social support networks are key considerations in creating environments that enable older people to age in place and maintain their autonomy and social connections.
	This definition emphasizes the need for housing policies and practices that support the well-being and independence of older populations.
Transportation	The transportation domain refers to the means by which people move from one place to another and, as such, could be read as “transport & mobility”. It includes public and private modes of transport such as buses, trains, taxis, cars, bicycles, and walking.
	This definition underscores the importance of affordable and accessible transportation in enabling older adults to remain independent, maintain mobility, social participation, and access to essential services within their community, whether urban, rural or remote. It emphasizes the need for age-friendly transportation systems that cater to the diverse needs of older populations and promote active aging and social inclusion.
Outdoor spaces & building	The outdoor spaces and buildings domain typically refers to the design, accessibility, and usability of public spaces, parks, streets, and buildings to accommodate the needs and preferences of older people. This domain focuses on creating environments that are safe, inclusive, and conducive to physical activity, social interaction, and community engagement for older residents. Key elements may include features such as well-maintained sidewalks and pathways, seating areas, public restrooms, accessible public transportation stops, pedestrian-friendly streets, age-friendly parks and recreational facilities, adequate lighting, and signage that is clear and easy to read. By enhancing the design and infrastructure of outdoor spaces and buildings, age-friendly initiatives aim to promote active aging, social participation, and overall well-being among older people, while also improving the overall liveability and accessibility of physical environments for residents of all ages.
Community support & health services	The community support and health services domain typically refers to the availability, accessibility, and quality of community-based support services, health and social care resources for older people. Key components may include programs and services that address the social, emotional, and physical needs of older people, such as home care services, caregiver support, health promotion initiatives, social activities, and access to healthcare facilities.
	This domain emphasizes the importance of creating a supportive environment that enables older people to age in place, maintain their health and well-being, and access the care and assistance they need to live independently and actively within their communities.
Communication & information	The communication and information domain refers to the accessibility and usability of communication technologies, media, and information resources for older people. This includes ensuring that older people have access to relevant and timely information, communication tools, and digital technologies that enable them to stay informed, connected, and engaged with their communities. Improving communication and information resources for older people can enhance their social participation, access to services, and overall quality of life. This definition underscores the importance of addressing digital inclusion and information accessibility for older people within age-friendly initiatives. Enhancing communication and information resources tailored to the needs of older populations can empower older people to remain active, informed, and connected in an increasingly digital world.
Social participation	The social participation domain refers to the engagement of older people in activities that provide them with opportunities for interaction, connection, and contribution to their communities. It can also be understood as social connection, which is a broader concept that incorporates social participation. This includes involvement in social, cultural, spiritual, and civic activities that promote social inclusion, mutual support, and a sense of belonging. Social participation is essential for maintaining mental and emotional well-being, combating social isolation, and fostering a sense of purpose and fulfilment in later life.
	This definition underscores the importance of creating environments that facilitate social engagement and meaningful participation for older people, thereby promoting active and healthy aging within communities.
Respect & social inclusion	The respect and social inclusion domain in age-friendly initiatives typically refers to the promotion of attitudes, behaviors, and policies that foster respect, dignity, and social inclusion for older people within the community. This domain emphasizes the importance of combating ageism, promoting intergenerational connections, and creating a culture of inclusivity that values the contributions and experiences of older people. Respect and social inclusion are closely related to the United Nations Decade of Healthy Ageing (2021-2030) (WHO, 2021a) action area of combating ageism and could be read as such.
	Key components of actions targeting this domain may include initiatives that raise awareness about age-related stereotypes and discrimination, promote positive images of aging, facilitate opportunities for older people to participate in community life, and encourage meaningful interactions between generations. By promoting respect and social inclusion, age-friendly initiatives aim to create environments where older adults feel valued, supported, and engaged, leading to improved social connections, mental well-being, and quality of life for older residents.
Civic participation & employment	The civic participation and employment domain typically refers to the opportunities and support systems available to older people for engaging in civic activities, volunteering, and remaining active in the workforce if desired. This domain encompasses initiatives that promote older adults’ participation in community decision-making, volunteering, lifelong learning, and continued employment or meaningful activities that contribute to society.
	By fostering civic engagement and employment opportunities for older people, age-friendly initiatives aim to enhance social inclusion, promote intergenerational connections, and harness the skills and experiences of older people for the benefit of the community.
AFCC programs	Programs specifically aimed at improving the lives of older people and highlighted in the objective of the systematic review (e.g., a review focused on age-friendly programs or multicomponent interventions targeting one or multiple domains) or EGM.

*Note.* Adapted from (WHO, 2007, 2015a, 2024b).

AFCC = Age-Friendly Cities and Communities.

### Why It Is Important to Develop the Mega-map

There is a wide variety of interventions to create and sustain age-friendly environments across all relevant sectors and domains of action described above. Likewise, there is a large and growing number of local, subnational, and national AFCC programs that either lead or support such interventions (WHO, 2023). While relevant systematic and scoping reviews on interventions covering one or more of the eight domains exist (Hérick de Sá et al., 2024; Hong et al., 2023; Montayre et al., 2022; Sánchez-González et al., 2020), there has not been, to the best of our knowledge, a comprehensive mapping of the available evidence across all domains of action and AFCC programs (WHO, 2007).

There are four directly relevant evidence and gap maps (EGMs) commissioned by the WHO that pertain to the United Nations Decade of Healthy Ageing (2021-2030) (Mikton et al., 2022; Welch et al., 2021, 2022, 2023). All four EGMs focus on specific outcomes, namely elder abuse, social isolation and loneliness (Mikton et al., 2022; Welch et al., 2023, 2024), and functional ability (Welch et al., 2021), leaving a whole range of outcomes relevant to older people no covered by available EGMs. Additionally, one EGM has been published with the Campbell Collaboration on social prescribing (Ghogomu et al., 2024), a category of intervention highly relevant to AFCC programs, but too narrow to meet the evidence need across several domains and diverse priorities. Nevertheless, the five EGMs synthesize evidence from interventions that fall within the eight domains of action and AFCC programs, serving as robust “building blocks” for the mega-map. Organizing the evidence based on the eight domains of action and AFCC programs is crucial for two reasons: (i) ensuring usability by age-friendly environments and AFCC partners, whose objective is to design evidence-based programs that impact multiple outcomes, and (ii) developing an evidence base to inform strategies and actions.

A mega-map provides a visual and interactive display of systematic reviews and EGMs (White et al., 2020), in this case focusing on domain-specific interventions and programs for creating and sustaining age-friendly environments. The mega-map will serve the following important purposes:

• Increase the discoverability and use of evidence on age-friendly environments by policy and decision-makers, program commissioners, and practitioners in countries.
• Identify areas where more research is needed and guide the commissioning of research in a more coordinated and strategic way (e.g., age-friendly environments-specific EGMs).
• Contribute to building an “evidence-action architecture” for the field of age-friendly environments (WHO, 2021b).
• Inform on priority areas to develop knowledge translation strategies.
• Help raise awareness of the topic with a view to increasing the extent to which it is perceived as a global priority.
• Help WHO refine its global strategy to leverage age-friendly environments globally within the United Nations Decade of Healthy Ageing 2020–2030 (WHO, 2021a).

### Objectives

This mega-map has two main objectives:

(1) Identify, map, and describe existing evidence from systematic reviews and EGMs on interventions and programs aimed at creating and sustaining age-friendly environments.
(2) Based on the evidence identified, mapped and described in objective 1, identify existing gaps in methods, interventions, outcomes, and geographies to assist researchers and policymakers in making evidence-informed research and funding decisions.

## Methods

### Defining mega-maps and EGMs

We will follow the Campbell Collaboration guidance for producing a mega-map (White et al., 2020).

EGMs are systematic evidence synthesis tools that visually present existing evidence within a specific topic or research area (Snilstveit et al., 2017; Welch et al., 2021, 2022, 2023; White et al., 2020). EGMs are organized as a two-dimensional matrix, with interventions represented as row headings and outcomes as column headings. Each cell in the matrix reflects studies providing evidence on the intersection of a specific intervention and outcome (White et al., 2020). A mega-map has a wider focus that spans a large sector or multiple sectors, including only systematic reviews and EGMs (White et al., 2020). Mega-maps provide an overview of the existing evidence, helping policymakers and practitioners prioritize areas for investment and research (Saran et al., 2020).

### Framework Development and Scope

We developed an intervention-outcome framework for this mega-map through a consultative process with stakeholders and key WHO publications on age-friendly environments (WHO, 2007, 2015a, 2015b, 2021a, 2023).

We adopted the population, intervention, comparison, outcome (PICO) framework to ensure a comprehensive and structured approach in shaping the scope of the map. The mega-map framework was developed in four stages:

(1) The preliminary framework matrix of interventions and outcomes was designed using insights from the WHO publications on age-friendly environments (WHO, 2007, 2015a, 2015b, 2021a, 2023).
(2) To determine further relevant interventions and outcomes, the authors engaged with experts in the field to identify key existing systematic reviews and EGMs on the topic.
(3) Stakeholder consultations were conducted to pilot the framework in the form of exercises using examples.
(4) The framework was updated in response to the stakeholders’ feedback, incorporating their insights and recommendations.

Interventions will be coded as relating to the eight domains of action (WHO, 2007) based on the primary focus of the mega-map. For the outcomes, we considered key WHO publications that list and define relevant outcomes for healthy aging (WHO, 2015b) and recent reports from the United Nations Decade of Healthy Ageing (2021-2030) (WHO, 2021a). Additionally, the outcomes were refined through stakeholder consultations, largely aiming to increase their usability for AFCC. The framework considers outcomes based on their proximal or distal nature, ranging from immediate effects to late consequences on health as we age (see more in Section Dimensions):

• Intermediate outcomes: behavior change, risk reduction and prevention, removing barriers, social participation, social inclusion, civic participation and employment, falls related, food and nutrition, and elder abuse.
• Domains of functional ability: Meet older people basic needs, Learn, grow, and make decisions, Be mobile, Build and maintain relationships, Contribute (WHO, 2015b).
• Domains of intrinsic capacity: Locomotor capacity (physical movement), Sensory capacity (such as vision and hearing), Vitality (energy and equilibrium), Cognitive capacity, Psychological capacity (WHO, 2021a).
• Economic impact: service use, quality, satisfaction, and cost effectiveness.
• Late health outcomes: mortality, life-expectancy, life-expectancy at 60 years, healthy life-expectancy, well-being, and quality life.

### Stakeholder Engagement

The Stakeholders were invited to participate in a series of meetings. They were selected based on their expertise in different areas of age-friendly environments, as well as expertise in evidence synthesis, including mega-maps and EGMs, while also ensuring balanced geographical and gender representation. Stakeholders’ profiles ranged from policy and decision-makers across sectors and different levels of government, researchers on age-friendly environments as well as researchers on specific topics and domains (e.g., transport, housing, urban planning, technology, health and social care, physical activity, falls prevention etc.), older people’s representatives, and technical staff from other UN Agencies. All WHO regions, eight intervention domains of action, and AFCC programs were covered by stakeholder expertise.

The expert consultations took place virtually on July 30, 2024, and March 20, 2025, with 12 participants in each of them (invited stakeholders not able to attend also provided contributions in writing). The main objectives of these meetings were to: familiarize key stakeholders with the nature and purposes of EGMs, introduce WHO’s ambition for the development of the EGM/mega-map and related practice focused tools, and consult key stakeholders on the framework and typology of age-friendly environments interventions and programs for this mapping exercise. Stakeholders suggested developing a mega-map, focusing on systematic reviews and EGMs, given the breadth and scope of the initiative. They also provided comments on the intervention-outcome framework. Overall, stakeholders suggested simplifying the framework, adding more granularity to the outcomes, and incorporating additional filters (e.g., to distinguish levels of urbanization). The framework was revised accordingly, and Stakeholders were consulted via email, followed by a third virtual workshop (April 2025) to capture final feedback on the revised framework included in this protocol. Overall, the proposed exercises to test the face validity and usability of the framework worked well, and Stakeholders did not encounter difficulties in identifying the evidence they were looking for.

### Conceptual Framework

**Figure 1:**
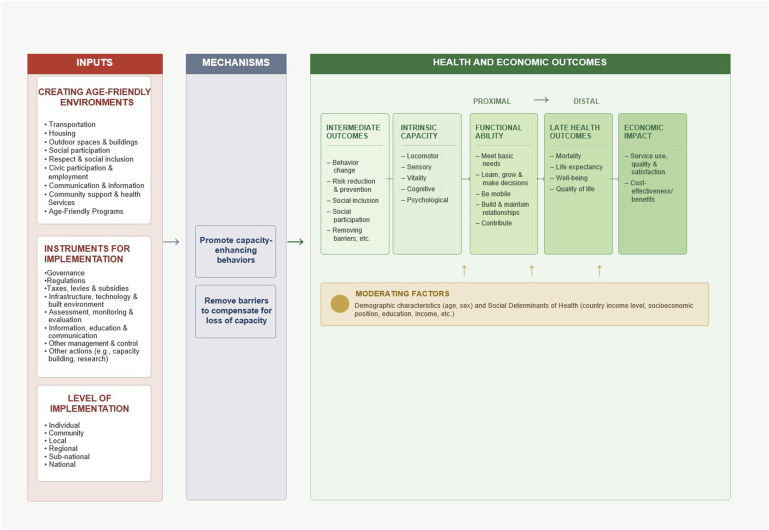
Conceptual Framework. Source: Adapted From (WHO, 2007, 2015a, 2024b)

Our conceptual framework (Figure 1) is based on the understanding that the physical and social environments in which we live can either enable or constrain healthy aging. This framework is built on the WHO World report on aging and health (WHO, 2015b), the Global age-friendly cities: a guide (WHO, 2007), and the Measuring the age-friendliness of cities: a guide to using core indicators (WHO, 2015a). Age-Friendly interventions and programs foster policies, services, settings, and structures that support and enable healthy aging by (WHO, 2007, 2025b):

• recognizing the wide range of capacities and resources among older people;
• anticipating and responding flexibly to aging-related needs and preferences;
• respecting their decisions and lifestyle choices;
• protecting those who are most vulnerable; and
• promoting their inclusion in and contribution to all areas of community life.

Age-friendly environments can be developed through a comprehensive, context-specific approach that interconnects eight key domains of action. These domains are not exhaustive but provide a framework for creating supportive environments for older people (WHO, 2007, 2018, 2023). These interventions can be implemented at various levels, ranging from individual to national, and utilize multiple instruments. These instruments include broad measures such as laws, regulations, and fees, modifications to the built environment, and information, education, and communication campaigns (WHO, 2024a). Interventions can also be implemented within a single domain, as multicomponent interventions across more than one domain or as part of AFCC programs. Upon consultation with stakeholders, we included a ninth intervention category within the mega-map framework to capture evidence of the two last examples (i.e., multicomponent interventions across domains and AFCC programs).

According to the WHO framework on healthy aging (WHO, 2015b), environments can improve the health of older people through two main mechanisms: promoting capacity-enhancing behaviors and removing barriers to compensate for the loss of capacity. In practical terms, capacity-enhancing environments are those that facilitate continued engagement in activities known to support physical, cognitive, and social functioning across the life course. Examples include the availability of safe and accessible spaces for physical activity, opportunities for meaningful social participation, and access to educational or learning initiatives in later life. Conversely, barrier-reducing strategies focus on modifying physical, social, and informational environments in ways that minimize constraints on daily activities, such as improving the accessibility of public spaces and enhancing transportation systems (WHO, 2015b).

Intrinsic capacity refers to the combined physical and mental resources of an individual, shaped over the life course by health conditions, personal characteristics, and biological factors. Functional ability, in turn, describes what individuals can do and be in ways they value, resulting from the interaction between intrinsic capacity and environmental conditions (WHO, 2015b).

By promoting capacity-enhancing behaviours and removing barriers, age-friendly environments help improve both intrinsic capacity and functional ability, ultimately enhancing the overall health and well-being of older people. Additionally, the framework considers the economic impacts of these interventions, highlighting potential cost savings, services use, quality, and satisfaction. Consistent with a previous EGM (Welch et al., 2021), the framework accounts for equity, recognizing the importance of assessing the distribution of impacts across population subgroups.  

### Dimensions

We will include systematic reviews and EGMs on interventions across the eight domains of action as well as AFCC programs. The inclusion of studies will be based on the PICO framework. We will follow the Campbell Collaboration’s guidelines (White et al., 2018a, 2018b), including the definition of systematic reviews, which states that reviews must have predefined inclusion criteria, an explicit and comprehensive search strategy, systematic and replicable coding and analysis of the key features and findings of the studies included in the review, assessment of the validity of findings in the included studies, as well as an integrative summary of the findings. To be eligible for inclusion, EGMs must have predefined inclusion criteria, an explicit and comprehensive search strategy, systematic analysis (reporting) of the included studies, as well as systematic and visual presentations of evidence in age-friendly environments. We will include systematic reviews and EGMs that included primary studies with any comparator. Studies will not be excluded based on their quality.  We will not include qualitative research. On-going systematic reviews and EGMs will be included.

#### Types of Intervention/Problem

We will include any intervention under the eight  domains of action (WHO, 2007) and AFCC programs aimed at creating and sustaining age-friendly environments to improve the lives of older people. The nine intervention categories are:

(1) Housing
(2) Transportation
(3) Outdoor spaces and buildings
(4) Community support and health services
(5) Communication and information
(6) Social participation
(7) Respect and social inclusion
(8) Civic participation and employment
(9) Age-Friendly Cities and Communities Programs

Included interventions will be categorized based on our conceptual framework by the level of implementation and instruments for implementation. The levels of implementation of the interventions will be categorized as individual, inter-individual, care-settings, community, city/metropolitan, sub-national/regional, or national levels (WHO, 2023, 2024a). The instruments for implementation will be classified into governance; regulations, taxes, levies and subsidies; infrastructure, technology and the built environment; assessment, monitoring and evaluation; information, education and communication; other management and control; and other actions (including capacity building; research, etc.) (WHO, 2024a). Table 1 presents operational definitions for each intervention category, while Table 2 presents the levels of implementation and instruments for the implementation of the interventions.

**Table 2:** Level of Implementation and Instruments for Implementation of Interventions and Programs

Level of implementation	Who provide the intervention/AFCC program OR where it was mainly implemented? OR Who is the main addressee of this intervention/AFCC Program? (WHO, 2024b)
National	National level interventions typically refer to government- or public authority-led initiatives undertaken at the country level that influence the economic, social and environmental characteristics of where people live, and determine their well-being. Usually, it is overarching for the whole population.
Sub-national/regional	Sub-national interventions focus on actions made at the level below the national government, often at the regional, provincial or state level to address local challenges. This includes decisions, actions, cooperation & collaboration within or across regions.
City/metropolitan	Interventions focus on actions made at the level below the sub-national government, often at city, metropolitan level, villages and townships
Community	Interventions at the community level can refer to geographically, physically, digitally or culturally defined environments. These tend to occur across a range of settings, such as public community spaces, workplace, and educational settings. It can also refer to rural or remote communities. Usually, it is more applicable to target sub-groups of the population rather than to the whole population.
Care settings	Care settings refer to the various environments where care services are provided to individuals in need of attention or support. This include a range of settings where interventions can be delivered to make these environments age-friendly, such as
	• community clinics
	• Primary care, including general practitioners, dentists, opticians, pharmacies
	• Secondary care, including hospitals, outpatient clinics, admission & emergency, ambulance
	• Tertiary care
	• Integrated care & transitions across settings
	• Long-term care settings
Inter-individual	This level refers to a type of intervention or treatment that focuses on interactions between individuals. This approach involves working with multiple individuals, such as couples, families, or groups, to address issues, improve relationships, or promote positive changes in behaviour or mental health. Inter-individual interventions often aim to enhance communication, problem-solving skills, and support systems among the individuals involved.
Individual	Individual level intervention refers to a type of intervention or treatment that is targeted at a single individual to address specific issues, promote behaviour change, or improve overall well-being. This type of intervention is tailored to the unique needs and circumstances of the individual and may involve strategies such as counselling, therapy, education, skill-building, or medical treatment. Individual level interventions are often used in healthcare, mental health, social services, and other fields to provide personalized support and assistance to help individuals achieve their goals and improve their quality of life.

Instrument for implementation	What is the main type of policy instrument/procedure how the intervention or AFCC program is implemented (WHO, 2024)
Governance	Involves changing how politics are made and how resources are allocated, for example may include increased intersectoral collaboration
Regulations	Involves setting legally binding standards, enforcement of new rules, laws or policies, regulations
Taxes, levies and subsidies	Involves creating financial incentives (can include disincentives)
Infrastructure, technology and the built environment	Involves the generation/adoption of new infrastructure, changes to the built environment or introducing/developing new technologies, also includes development/introduction of new/different products
Assessment, monitoring and evaluation	Involves some form of monitoring/data collection about correct/desired status
Information, education and communication	Involves raising awareness, lobbying, providing information, skill development and advocacy.
Other management and control	Involves adopting new/different ways of management and/or strategies, or performing standard actions under different circumstance
Other actions	In case there is a concern with all other instruments, can be used in conjunction with other instruments

*Note.* Adapted from (WHO, 2024b).

If reviews include a subset of interventions that are not eligible, we will code details on the analysis of groups of studies which meet the PICO criteria of this mega map. We will not code primary studies individually but rather the disaggregated group analyses reported within systematic reviews that meet the PICO criteria of this map. When a systematic review or EGM includes a subset of interventions or outcomes that align with our eligibility criteria, we will extract and code information on the disaggregated analyses presented by the review authors.

#### Population

For this mega-map, we will accept systematic reviews and EGMs looking at people from 50 years old onwards, considering that intervention studies commonly recruit participants at earlier ages to account for the lag between the intervention and its impacts. If systematic reviews and EGMs include younger and older people, we will include those where data can be disaggregated. Using 50 years as the lower boundary enables the mega-map to capture evidence on early aging transitions, such as vision and hearing loss that are clinically and functionally relevant for interventions that support functional ability, social participation and well-being across later adulthood (Zaninotto et al., 2024).

#### Outcome Measures

We will consider five outcome categories:

• Intermediate outcomes
• Domains of functional ability (WHO, 2015b)
• Domains of intrinsic capacity (WHO, 2015b)
• Economic impact
• Late health outcomes

Table 3 lists the outcome categories and sub-categories.

**Table 3:** Outcome Categories

Outcomes	Operational definition
Intermediate outcomes	Short- to mid-term changes in health status or related outcomes: Behavior change, Risk reduction and prevention, Removing barriers, Social participation, Social inclusion, Civic participation and employment, Falls related, Food and nutrition, Elder abuse, etc.
Domains of functional ability (WHO, 2021b)	
Meet older people basic needs	Ability of older people to manage and meet their immediate and future needs to ensure an adequate standard of living as defined in United Nations Article 25 of the universal declaration of human rights. This ability includes older people being able to afford an adequate diet, clothing, suitable housing, and healthcare and long-term care services. It also extends to having support to minimize the impact of economic shocks that may come with illness, disability, losing a spouse or the means of livelihood
Learn, grow, and make decisions	Include efforts to continue to learn and apply knowledge, engage in problem solving, continue personal development, and be able to make choices. Continuing to learn enables older people to have the knowledge and skills to manage their health, to keep abreast of developments in information and technology, to participate (for example, by working or volunteering), to adjust to aging (for example, to retirement, widowhood or becoming a caregiver), to maintain their identity and to keep interested in life
Be mobile	It refers to movement in all its forms, whether powered by the body (with or without an assistive device) or a vehicle. Mobility includes getting up from a chair or moving from a bed to a chair, walking for leisure, exercising, completing daily tasks, driving a car and using public transport
Build and maintain relationships	A broad range of relationships are important to older people, including their relationships with children and other family members, intimate relationships, and informal social relationships with friends, neighbours, colleagues and acquaintances, as well as more formal relationships with community-service providers. For example, the quantity and quality of interpersonal relationships, and the levels of trust within, and feeling of belonging to, a network of people with shared interests
Contribute	A myriad of contributions that older people make to their families and communities, such as assisting friends and neighbours, mentoring peers and younger people, and caring for family members and the wider community. The ability to contribute is closely associated with engagement in social and cultural activities
Domains of intrinsic capacity – (WHO, 2021b)	
Locomotor capacity (physical movement)	A person’s physical capacity to move their body is termed locomotor capacity and is vital to enable people to be mobile, maintain independence and prevent dependence on care. Locomotor capacity is characterized by the functions of joints, bones, reflexes and muscle strength.
Sensory capacity (such as vision and hearing)	This refers to the capacity of the sensory nervous system to process information and respond to stimuli. It consists of functions of the senses, such as seeing, hearing, tasting, smell and sensation.
Vitality (energy and equilibrium)	Biophysiological status of an individual and the capacity for maintaining homeostasis in the face of usual daily exposures, as well as more extreme and unusual or unexpected challenges such as injury or infection.
Cognitive capacity	Person’s capacity to perform a range of mental functions, including but not limited to functions for orientation, sustaining and shifting attention, psychomotor, memory, language, calculation, thought; and higher-level cognitive functions that involve decision-making, abstract thinking, making and carrying out plans, and deciding which behaviours are appropriate under what circumstances.
Psychological capacity	Related to emotional functions – i.e., the mental functions related to the feeling and affective components of the process of the mind.
Late health outcomes (WHO, 2015b)	
Life expectancy (at 60)	The average number of years that a 60-year-old can expect to live if he or she is subject to the age-specific mortality rate during a given period
Life expectancy (at birth)	The average number of years that a newborn would be expected to live if he or she is subject to the age-specific mortality rate during a given period
Healthy life expectancy	Population-based measure of the proportion of expected life span estimated to be healthful and fulfilling, or free of illness, disease and disability according to social norms and perceptions and professional standards
Well-being and quality of life	A general term encompassing the total universe of human life domains, including physical, mental and social aspects, that make up what can be called a “good life”
Economic impact	
Service use, quality, satisfaction	This includes information on the use and intensity of service utilization as a result of the intervention. The benefit component includes benefits derived or forgone due to the quality of and satisfaction with target interventions.
Cost effectiveness/benefit	Refers to economic efficiency and value derived from implementing programs, services, or environmental modifications that support older adults’ health, safety, and well-being.
	Evidence will consist of comparative analysis in the form of cost-effectiveness, cost-utility, cost-benefit and/or social return on investment evaluations.

*Note.* Adapted from (WHO, 2015b, 2021b).

We will not use outcomes as eligibility criteria; however, eligible systematic reviews and EGMs must assess interventions under the eight domains of action (WHO, 2007) or AFCC programs aimed at creating and sustaining age-friendly environments to improve the lives of older people. Evidence relating to potential harm will also be documented.

### Other Eligibility Criteria

#### Types of Location/Situation

We will include evidence coming from all countries and organize it according to the WHO regions (African Region; Eastern Mediterranean Region; European Region; Region of the Americas; South‑East Asia Region; Western Pacific Region) (WHO, 2019) and the World Bank classification by income: low-income economies, lower-middle income economies, upper-middle income economies, high-income economies (World Bank, 2022) as a filters. We will not exclude systematic reviews and EGMs that do not provide information on the countries the evidence included comes from.

#### Types of Settings

We will consider people from 50 years old onwards living in cities and communities of any size and location in urban, peri-urban and rural areas. We will include evidence on interventions and programs at any level of implementation, whether at the local (e.g., city or community), regional (e.g., province, state, prefecture), or national levels, as well as in any settings (e.g., residential or personal homes, nursing homes, and community centres).

### Search Methods and Sources

The initial search strategy was developed by reviewing key systematic reviews and EGMs related to age-friendly environments for older people, and public health interventions targeting aging populations. These reviews provided a foundation of commonly used search terms, which were refined in subsequent search trials, combining the team’s expertise in both evidence synthesis and age-friendly environments. To ensure the robustness of the search strategy, we cross-checked the results of the initial search trial against a curated list of systematic reviews we would expect to retrieve to confirm that key systematic reviews were consistently captured by our searches. The search trial captured 23 of the 24 (95%), *n* = 23/24) systematic reviews on the curated list. The main focus of this exercise was to improve the sensitivity of the search strategy rather than deliberately formulating search strings and search sources that are over-inclusive. This approach might increase the number of citations to be screened, but it will reduce the risk of missing any relevant systematic reviews.

The search strategy aims to find both academic and grey literature. To that end, a two-pronged search strategy will be employed for this mega-map: (i) a formal search of academic databases using pre-defined and explicit search strings and Boolean operators; (ii) a formal search of grey literature in key organizational websites using keywords but applying full search strings in cases where institutional databases allow the application of Boolean operators. The full search strategy is presented in Appendix 1. The iterative trials for the search strategy ensures validity while expert input and the targeted inclusion of both academic and grey literature sources will ensure that the resulting map provides a comprehensive overview of the evidence on age-friendly environments.

We will search the following databases from inception with no date or language restrictions: PubMed (via NCBI), Embase (via Elsevier), Web of Science (via Web of Science Core Collection), Scopus (via Elsevier), Campbell library (via Campbell Collaboration), Cochrane Database of Systematic Reviews (via Wiley Online Library), 3ie Development Evidence Portal (via International Initiative for Impact Evaluation), Global Index Medicus (GIM) (via WHO), Transport Research International Documentation (TRID) (via TRB/OECD ITF platform), WHO Library Institutional Repository for Information Sharing (IRIS) (via WHO), World Bank Open knowledge repository (via World Bank), AgeInfo (via Centre for Policy on Ageing (CPA)) and via EBSCO CINAHL, ERIC, PsycINFO, Academic Source Ultimate, Business Source Ultimate, Social work abstracts, Communication and Mass Media Complete, Political science complete, Econlit, Africa Wide Information, Greenfile (all via EBSCOhost). Studies published in any of the six official WHO languages will be considered. To ensure comprehensive inclusion and minimize language bias, searches, screening and coding of non-English studies will be conducted by native-language co-authors. We will search for grey literature in the specialist organizational websites and research institutes (*n* = 24) listed in Table 4.

**Table 4:** Grey Literature Organizational Websites and Research Institutes

1. Abdul Latif Jameel Poverty Action Lab (J-PAL): https://www.povertyactionlab.org/evaluations
2. African Development Bank (AfDB): https://www.afdb.org/en
3. Asian Development Bank: https://www.adb.org/
4. Bill & Melinda Gates Foundation: https://www.gatesfoundation.org/
5. Campbell Library: https://www.campbellcollaboration.org/campbell-library/
7. European Union (EU): https://europa.eu/
8. Global Alliance of National Human Rights Institutions (GANHRI): https://ganhri.org/
9. Global Index Medicus (GIM): https://www.globalindexmedicus.net/
10. Help Age: https://www.helpage.org/oldernotover/
11. Innovations for Poverty Action (IPA) Publications: https://poverty-action.org/
12. Inter-American Development Bank: https://www.iadb.org/en
13. International Initiative for Impact Evaluation: 3ie Development Evidence Portal: https://developmentevidence.3ieimpact.org/
14. International Institute for Environment and Development: www.iied.org/
15. Transport Research International Documentation (TRID): https://trid.trb.org/
16. UN Framework Convention on Climate Change: https://unfccc.int/
17. United Nations Population Fund (UNFPA): https://www.unfpa.org/
18. UN Women: https://www.unwomen.org/en
19. United Nations Department of Economic and Social Affairs (UNDESA): https://www.un.org/development/desa/ageing/
20. United Nations Economic Commission for Europe (UN-ECE): https://www.un.org/development/desa/ageing/
21. USAID Evaluations Clearinghouse: https://dec.usaid.gov/
22. WHO Library _ Institutional Repository for Information Sharing (IRIS): https://iris.who.int/
23. World Bank Open knowledge repository: https://openknowledge.worldbank.org/home
24. World Health Organization: https://www.who.int/

Using citation chaser, we will screen reference lists of all included systematic reviews and EGMs in EPPI-Reviewer to identify additional systematic reviews and EGMs that may have been missed by the structured searches. In addition, we will conduct forward citation chasing for all included reviews and EGMs to identify any newer relevant studies that have cited included studies. All records will be exported via citation chaser and imported into EPPI-Reviewer for deduplication and screened against the same eligibility criteria used for the database searches. The results will be reflected in the PRISMA-flow diagram. This approach will allow for sensitivity and reduce retrieval and indexing. We will also contact experts from each of the nine intervention categories to confirm that we have not overlooked any important evidence within their domain of expertise.

### Analysis and Presentation

#### Report Structure

The reporting structure will follow Campbell Collaboration guidelines (White et al., 2018a, 2018b), with the standard headings: abstract, plain-language summary, background, methods, results, discussion, and conclusion.

The report will outline the study flow, detailing both included and excluded studies with the addition of a PRISMA flow diagram (Page et al., 2021). Additionally, the report will include the conceptual framework as well as tables and figures summarizing the distribution of included studies across all the coding categories such as study designs, quality of systematic reviews, domains of action types of outcomes, population characteristics, settings, and geographic distribution.

**Figure 2:**
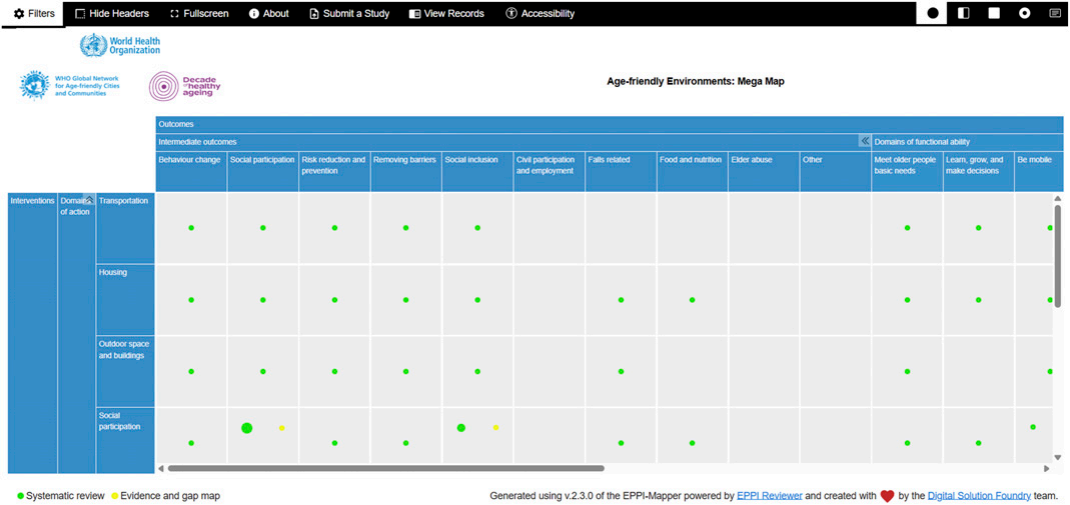
Draft Mega-Map Framework. Source: Generated Using EPPI-Mapper (v2.3.0) (Digital Solution Foundry & EPPI-Centre, 2024)

 The findings will be presented visually in the form of a mega‑map, in which interventions are organized as rows and outcomes are organized as columns. Bubbles of varying sizes will indicate the number of studies, while different colours will differentiate systematic reviews from EGMs. See a sample of the map in Figure 2. The number of included studies and coded information will determine which filters will be used in the map.

#### Filters for Presentation

In addition to intervention and outcomes, the following filters will be used to code systematic reviews and EGMs:

(1) Study characteristics: publication date of included studies, WHO official languages of the publication, type of evidence (Systematic review, EGMs), country in which the interventions were conducted, WHO region (African Region; Eastern Mediterranean Region; European Region; Region of the Americas; South‑East Asia Region; Western Pacific Region), World Bank classification by income (low-income economies, lower-middle-income economies, upper-middle-income economies, high-income economies), degree of urbanization for location of interventions (city or densely populated areas, towns and semi-dense areas or intermediate dense areas, rural areas or thinly populated areas), systematic review quality.
(2) Intervention characteristics: equity (yes/no study aimed to look at equity considerations and health inequalities based on PROGRESS‑Plus framework) (Oliver et al., 2008; O’Neill et al., 2014), domains of action, level of implementation (national, sub-national/regional, city/metropolitan, community, care settings, inter-individual, individual), instruments for implementation (governance, regulations, taxes, levies and subsidies, infrastructure, technology, and the built environment, assessment, monitoring and evaluation, information, education and communication, other management and control, other actions including capacity building)
(3) Population characteristics: age groups, sex, and other sociodemographic factors as well as disability status.

#### Dependency

We will treat multiple reports of the same systematic review or EGM as one review. A systematic review or EGM reporting on multiple interventions categories and/or outcomes will appear in multiple cells on the map, once for each intervention and outcome identified.

### Data Collection and Analysis

#### Screening and Study Selection

We will use EPPI Reviewer 6 (Thomas et al., 2020) to manage the entire screening process. All relevant citations from academic sources will be imported into EPPI Reviewer 6, while search results from organizational websites and citation searches will be captured in MS Word and then imported if relevant. Grey literature not already in EPPI Reviewer will be added manually. Duplicates will be excluded using the duplicate control function.

A manual screening process will assess eligibility on EPPI Reviewer 6. Initially, a double-screening exercise will be conducted at the title and abstract level using the inclusion criteria, with decisions recorded in EPPI Reviewer 6. To ensure quality, 10% of studies will be double screened by two reviewers (RN and TK), and discrepancies will be discussed. Individual screening will proceed. Based on these discussions, the coding tool and variable definitions will be updated to address any ambiguities. A second round of 10% duplicate screening will then be conducted using the updated tool to assess whether agreement has improved before proceeding with single screening of the remaining records.

Following title and abstract screening, a full-text screening will be conducted for studies meeting the inclusion criteria. Two reviewers will independently screen these studies, and any disagreements will be resolved by a senior review team member. The output will be a set of studies suitable for inclusion in the mega-map, reported using a PRISMA flow chart.

All screenings will follow the eligibility criteria listed in Appendix 2.

#### Data Extraction and Management

We will develop and pilot test a data extraction code set in EPPI-Reviewer 6 (Thomas et al., 2020) for data collection (see Appendix 4). We will use a set of included studies for testing. All reviewers (PN, RN, TK) will code the same studies, and the coding will be assessed for agreement. Any discrepancies identified using the reconciliation function in EPPI-Reviewer 6 will be discussed, and the description of the coding criteria will be modified for clarity as necessary. After the pilot, reviewers (PN, RN, TK) will individually extract and code data. Automation and text mining will not be used for coding. To enhance clarity and reliability, data extraction will employ a piloted and standardized coding tool to ensure consistent application of codes. The pilot phase will identify ambiguous items and improve clarity before the full-scale coding. Based on the results and discussion of the pilot the coding tool will be updated accordingly. This transparent and iterative process will strengthen the reliability, validity and reproducibility of data extraction similar with the quality assurance that will be applied to duplicate screening.

We will code the study characteristics (publication date, language, type of evidence, systematic review quality), categories and subcategories of interventions and other intervention characteristics (equity focus defined through the use of the PROGRESS-Plus framework: Place of residence, Race/ethnicity, Occupation, Gender, Religion, Education, Socio-economic status, and Social capital, plus Age, Disability, and other context-specific vulnerabilities) (Oliver et al., 2008; O’Neill et al., 2014), domains of action, level of implementation and instruments for implementation), outcome domains and subdomains, population characteristics, settings, locations (by country, WHO region, and World Bank income classification, degree of urbanization for location of interventions).

We will not contact authors of systematic reviews for any missing information given the expected size of the map (over 500 studies).

#### Tools for Assessing Risk of Bias/Study Quality of Included Reviews

Given the expected large volume of studies, the quality of the included systematic reviews will be assessed using the Rapid Appraisal Fast-and-Frugal Decision Tree (Lorenz et al., 2024). The Rapid Appraisal Fast-and-Frugal Decision Tree allows for rapid yet structured appraisal. This streamlined approach is better suited for our mega-map as it will allow many systematic reviews to be appraised within limited periods. The Rapid Appraisal Fast-and-Frugal Decision Tree works by focusing on a small number of highly predictive cues—in this case, selected AMSTAR2 items (Shea et al., 2017)—and guiding users through a binary decision-making process. Each item in the tree has an “exit leaf”, meaning that a single response can be sufficient to classify a systematic review as either high or low quality (Lorenz et al., 2024) The tool makes use of three AMSTAR2 criteria as decision nodes: list of excluded studies with reasons (AMSTAR2 item 7) the presence of a pre-registered protocol (AMSTAR2 - item 2), and a comprehensive search strategy (AMSTAR2 - item 4). This tool will enable the team to maintain a balance between feasibility and methodological rigour (Lorenz et al., 2024). Any disagreements will be resolved by discussion.

Critical appraisal will not be carried out for EGMs included.

#### Methods for Mapping

We will use the EPPI-Mapping tool (Digital Solution Foundry & EPPI-Centre, 2020) to develop the mega- map.

## Supplemental Material

Supplemental Material - Interventions and Programs for Age-Friendly Environments: A Mega-Map Supplemental Material for Interventions and Programs for Age-Friendly Environments: A Mega-Map by Amanda Paula Fernandes, Ruvimbo Nhandara, Louise Lafortune, Thomas Katairo, Promise Nduku, Thiago Herick de Sá in Campbell Systematic Reviews.
