# Supplementary material for: PROTOCOL: Mega-Map of Systematic Reviews and Evidence and Gap Maps on Interventions and Programs for Age-Friendly Environments
Source: Campbell Syst Rev. 2026 Jul 15;22(3):18911803261437804. doi: 10.1177/18911803261437804 (PMC13376465; doi:10.1177/18911803261437804)
Supplement: Supplemental Material - Interventions and Programs for Age-Friendly Environments: A Mega-Map [file sj-pdf-1-cam-10.1177_18911803261437804.pdf]

Appendices

Appendix 1 - Search strategy

A generic search strategy was developed as shown below and will serve as the foundation for all database searches. This core strategy will be adapted for each database using Polyglot Search Translator (https://polyglot.sr-accelerator.com/). Polyglot is an online tool that converts a single comprehensive search string into equivalent syntax for multiple databases.

The translated searches will be tailored to the specific requirements of each database interface, for example PubMed (via NCBI) example below. This process ensures consistency in search terms across platforms while maintaining compatibility with each database's unique structure and operators.

A. Age terms

(ageing or aging or elder* or eldest or geriatric* or gerontolog* or “age-friendly” or “healthy ageing” or “active ageing” or ageing or aging or “care home resident*” or “community-dwelling” or elder* or frail* or geriatric* or “late life” or “later life” or “mature adult*” or nonagenarian* or “nursing home resident*” or octogenarian* or “old age” or “oldest old” or pensioner* or postmenopaus* or “post-menopaus*” or retired or retiree* or senior citizen* or seniors or (">=50# years old" or ">50# years old" or ">=60# years old" or ">60# years old" or ">=70# years old" or ">7#0 years old" or ">=80# years old" or ">80# years old" or ">=90# years old" or ">90# years old") or ((older adj3 adult*) or old age* or (older adj3 client*) or (older adj3 communit*) or (older adj3 female*) or (older adj3 individual*) or (older adj3 inpatient*) or (older adj3 in-patient*) or (older adj3 male*) or (older adj3 men) or (older adj3 outpatient*) or (older adj3 out-patient*) or (older adj3 patient*) or (older adj3 people) or (older adj3 person*) or (older adj3 population*) or (older adj3 women)) or "age in place" or "aging in place" or “ageing in place” or ((active or healthy) N3 (ageing or aging)) or (friendl* N3 (age or ages or aged or aging or ageing or elder*)))

B) Methodology terms (s)

(systematic* adj2 review*) or "meta-analy*" or "meta analy*" or “evidence map” or“evidence maps” or “evidence mapping” or “evidence and gap map” or EGM or “evaluation map” or evaluation maps or “evaluation mapping” or “systematic map” or “systematic maps” or “systematic mapping” or “descriptive map” or “descriptive maps” or “descriptive mapping”

C) Intervention terms (domain-specific)

Domain 1 terms: Transportation

("active* transport*" or "active* travel*" or ((bicycl* or cycl* or driv* or motorcycl* or walk*) N3 (coach* or course* or educat* or instruct* or lesson* or school* or train*)) or ((bicycl* or cycli* or driv* or motorcycl* or motorist* or passenger* or pedestrian* or rider* or walk* or traffic* or transport* or automobile* or bus or buses or car or cars or carpark* or "dial a ride" or garage* or metro or railway* or parking* or sidewalk* or subway* or taxi* or train or trains or underground or vehicle* or crossing* or highway* or lane or lanes or road or roads or street*) N3 (abilit* or access* or afford* or behaviour* or behavior* or capacit* or danger* or difficult* or ergonomic* or habit* or safe* or stable or stability or unsafe or ((fare* or price* or ticket*) N3 (discount* or free or reduc*)) or accident* or collision* or crash* or hazard* or incident* or speed*)) or ((bicycl* or cycli* or driv* or motorcycl* or automobile* or bus or buses or car or cars or metro or railway* or subway* or taxi* or train or trains or underground or vehicle*) N3 (adapt* or alteration* or modif* or support or usability)))

Domain 2 terms: Housing

((ambient* N3 assist* N3 living) or ((smart or automat*) N3 home*) or (smart N3 environment*) or ((home* or hous* or residen* or accommodation* or bath* or bedroom* or kitchen* or room* or shower* or stair* or toilet*) N3 (access* or adapt* or alteration* or modif* or renovat* or support or usability)) or ((elevator* or handrail* or "hand rail*" or lift or lifts or ramp or ramps or gerontechnolog* or "voice assistant*" or (service N3 robot*) or ((assist* or intelligen* or safe* or "self-help*" or welfare*) N3 (aid or aids or device* or platform* or robot* or solution* or technolog*))) and (home* or hous* or residen* or accommodation* or city or cities or communit* or environment* or neighb* or rural or societ* or town* or urban or village*))) or “naturally occurring retirement communi*” or NORC or housing or “cohousi*” or “co-hous*” or “university based retirement communi*” or “UBRC*” or apartment* or bungalow* or dwelling* or housing or landlord* or rehous* or squatter* or tenant* or bedsit or “bedsitting” or “bed sit” or “bed sitting” or “residential care” or highrise* or “high rise*” or homeowner* or “home owner*” or “indoor air qualit*” or “living environment*” or “live environment*” or “built environment” or “living quarter*” or multistor* or “multi stor*” or owner* occup* or towerblock* or “tower block*” or “cottage*" or "flat*" or "houses" or home or stair or staircase or stairwell or maisonette* or indoor*

Domain 3 terms: Social participation

“social participat*” or “community involve*” or “community-dwelling” or “ engage*” or “active citizen*” or “social involve*” or “community participat*” or “public engag*” or “societal engage*” or “participatory citizenship” or “active participat*” or “collective action” or “engage* in society” or “group participat*” or “collaborat* involve*” or “social integrat*” or “involve* in public life” or “social connect*” or “social health” or “social capital”

Domain 4 terms: Respect and social inclusion

"social inclusion" or "community inclusion" or "e-inclusion" or "digital inclusion" or "social cohesion" or "community cohesion" or "neighbo* cohesion" or "social involvement" or "community involvement" or "social integration" or "community integration" or "social engagement" or " engagement" or "community engagement" or "intergeneration*" or "social recognition" or "information and communication technolog*" or "social exclusion" or "neighbo* inclusion" or "neighbo* exclusion" or "community participation" or "social participation" or "ageism" or "agism" or "age stereotyp*" or "ageing stereotyp*" or "aging stereotyp*" or "age discrimination" or "ageing discrimination" or "aging discrimination" or "digital divide" or "social interaction*" or "social responsabilit*" or "social capital" or "social networks" or "access services" or "access information" or "access opportunit*" or "access faciliti*" or "access volunteer*" or "access learning" or "social exchange*" or "solidarity"

Domain 5 terms: Civic participation and employment

"civil participation" OR "citizen participation" OR "community engagement" OR "public participation" OR " engagement" OR "political participation" OR "employ*" OR "job" OR "occupation" OR "work*" OR "career" OR "labour market" or "labor market" OR "job market" OR "workforce" OR independence

Domain 6 terms: Communication and information

communication or information or negot* or counselling or learning or literacy or training or coaching

Domain 7 terms: Community support and health services

"community support" OR "community assist*" OR "community aid" OR "local support" OR "social support" OR "community resource*" OR "community service*" OR "mutual aid" OR "community welfare" OR "community development" OR "grassroot* support" OR "volunteer support" OR "community engagement*" OR "community involvement*" OR "health service*" OR "healthcare service*" OR "medical service*" OR "healthcare facility*" OR "healthcare system*" OR "healthcare delivery" OR "healthcare access" OR "healthcare utilisation" OR "healthcare utilization" OR "healthcare management" OR "healthcare policy" OR "healthcare administration" OR "healthcare quality" OR "healthcare reform" OR “assisted living” or AL or "retirement home*" OR "retirement communit*" OR "supportive hous*" OR "long-term care" OR "senior care" OR "aged care"

Domain 8 terms: Outdoor space and buildings

(((urban or rural) adj3 (plan* or develop* or design*)) or ((built or green or neighb* or street* or rural or urban or cycling or driving or walking) adj3 environment*) or ((neighb* or rural or urban) adj3 space*) or amenit* or "communit* environment*" or greener* or greenness or greenspace* or greenway* or ((green or blue) adj3 (area* or space*)) or "land use*" or streetscape* or (street* adj3 connect*) or "rural renewal" or "urban renewal" or "rural form*" or "urban form*" or walkabilit* or ((population or residential or retail or rural or urban) adj3 densit*) or ((aesthetic* or garden or gardens or park or parks or ((plan* or develop* or design*) adj3 (facility or facilities)) or ((outdoor* or public) adj3 (space* or building* or facility or facilities*)) or ((indoor* or living or natural or objective or outdoor* or perceived or physical or urban or rural) adj3 environment*)) and (home* or hous* or residen* or accommodation* or city or cities or communit* or neighb* or rural or societ* or town* or urban or village*)) or ((neighb* adj3 (space* or building* or facility or facilities)) and (home* or hous* or residen* or accommodation* or city or cities or communit* or rural or societ* or town* or urban or village*)) or ((communit* adj3 (space* or building* or facility or facilities)) and (home* or hous* or residen* or city or cities or neighb* or rural or societ* or town* or urban or village*)) or (infrastructure* and (city or cities or communit* or cycling or driving or neighb* or pedestrian or public or rural or town* or transport* or urban or village*))) OR

(((emission* OR air OR “particulate matter” OR “ambient particulate” OR “ultrafine particulate*” OR “ultrafine particle*” OR UFP) N4 (control* OR regulation* OR policy OR policies OR guideline OR intervention OR act OR directive* OR vehicle OR transport* OR traffic OR automobile* OR car OR cars OR industr* OR fuel OR “emission filter*” OR cooking OR heating OR cookstove* OR stove* OR “power generat*” OR zone* OR Olympic OR residential OR “wood burning” OR mobile OR Low OR Lower OR Lowered OR reduc* OR improv* OR clean* OR congestion* OR “coal burning” OR ban OR bans)) OR ((improved or clean* or “low emission” or efficient*) N1 (cookstove* or stove or stoves or heater))) OR ((air N2 (pollut* OR quality OR ambient)) OR (atmospher* N2 pollut*) OR (“particulate matter” OR “ambient particulate” OR “ultrafine particulate*” OR “ultrafine particle*” or UFP) OR (“coarse particle*” OR “black smoke” or “black carbon” or “elemental carbon” OR “wood smoke”)))

PubMed (via NCBI)
Fields searched: Title and Abstract

Category		Search terms
A) Age terms	1	(ageing[Title/Abstract] OR aging[Title/Abstract] OR elder*[Title/Abstract] OR eldest[Title/Abstract] OR geriatric*[Title/Abstract] OR gerontolog*[Title/Abstract] OR age-friendly[Title/Abstract] OR "healthy

ageing"[Title/Abstract] OR "active ageing"[Title/Abstract] OR ageing[Title/Abstract] OR aging[Title/Abstract] OR "care home resident*"[Title/Abstract] OR community-dwelling[Title/Abstract] OR elder*[Title/Abstract] OR frail*[Title/Abstract] OR geriatric*[Title/Abstract] OR "late life"[Title/Abstract] OR "later life"[Title/Abstract] OR "mature adult*"[Title/Abstract] OR nonagenarian*[Title/Abstract] OR "nursing home resident*"[Title/Abstract] OR octogenarian*[Title/Abstract] OR "old age"[Title/Abstract] OR "oldest old"[Title/Abstract] OR pensioner*[Title/Abstract] OR postmenopaus*[Title/Abstract] OR post-menopaus*[Title/Abstract] OR retired[Title/Abstract] OR retiree*[Title/Abstract] OR "senior citizen*"[Title/Abstract] OR seniors[Title/Abstract] OR (">=50* years old"[Title/Abstract] OR ">50* years old"[Title/Abstract] OR ">=60* years old"[Title/Abstract] OR ">60* years old"[Title/Abstract] OR ">=70* years old"[Title/Abstract] OR ">7*0 years old"[Title/Abstract] OR ">=80* years old"[Title/Abstract] OR ">80* years old"[Title/Abstract] OR ">=90* years old"[Title/Abstract] OR ">90* years old" [Title/Abstract]) OR ((older[Title/Abstract] AND adult* [Title/Abstract]) OR "old age*"[Title/Abstract] OR (older[Title/Abstract] AND client* [Title/Abstract]) OR (older[Title/Abstract] AND communit* [Title/Abstract]) OR (older[Title/Abstract] AND female* [Title/Abstract]) OR (older[Title/Abstract] AND individual* [Title/Abstract]) OR (older[Title/Abstract] AND inpatient* [Title/Abstract]) OR (older[Title/Abstract] AND in-patient* [Title/Abstract]) OR (older[Title/Abstract] AND male* [Title/Abstract]) OR (older[Title/Abstract] AND men [Title/Abstract]) OR (older[Title/Abstract] AND outpatient*

			[Title/Abstract]) OR (older[Title/Abstract] AND out-patient* [Title/Abstract]) OR (older[Title/Abstract] AND patient* [Title/Abstract]) OR (older[Title/Abstract] AND people [Title/Abstract]) OR (older[Title/Abstract] AND person* [Title/Abstract]) OR (older[Title/Abstract] AND population* [Title/Abstract]) OR (older[Title/Abstract] AND women [Title/Abstract])) OR "age in place"[Title/Abstract] OR "aging in place"[Title/Abstract] OR "ageing in place"[Title/Abstract] OR ((active[Title/Abstract] OR healthy [Title/Abstract]) AND (ageing[Title/Abstract] OR aging [Title/Abstract])) OR (friendl*[Title/Abstract] AND (age[Title/Abstract] OR ages[Title/Abstract] OR aged[Title/Abstract] OR aging[Title/Abstract] OR ageing[Title/Abstract] OR elder* [Title/Abstract])))
B)	Methodology term (s)	2	(systematic*[Title/Abstract] AND review* [Title/Abstract]) OR meta-analy*[Title/Abstract] OR "meta analy*"[Title/Abstract] OR "evidence map"[Title/Abstract] OR "evidence maps"[Title/Abstract] OR "evidence mapping"[Title/Abstract] OR "evidence and gap map"[Title/Abstract] OR EGM[Title/Abstract] OR "evaluation map"[Title/Abstract] OR "evaluation maps"[Title/Abstract] OR "evaluation mapping"[Title/Abstract] OR "systematic map"[Title/Abstract] OR "systematic maps"[Title/Abstract] OR "systematic mapping"[Title/Abstract] OR "descriptive map"[Title/Abstract] OR "descriptive maps"[Title/Abstract] OR "descriptive mapping"[Title/Abstract]
C)	Intervention terms (domain-specific)	3<br><br>Domain 1: Transportation	("active* transport*"[Title/Abstract] OR "active* travel*"[Title/Abstract] OR ((bicycl*[Title/Abstract] OR cycl*[Title/Abstract] OR driv*[Title/Abstract] OR motorcycl*[Title/Abstract] OR walk* [Title/Abstract]) AND (coach*[Title/Abstract] OR course*[Title/Abstract] OR educat*[Title/Abstract] OR instruct*[Title/Abstract] OR

lesson*[Title/Abstract] OR school*[Title/Abstract] OR train* [Title/Abstract])) OR ((bicycl*[Title/Abstract] OR cycli*[Title/Abstract] OR driv*[Title/Abstract] OR motorcycl*[Title/Abstract] OR motorist*[Title/Abstract] OR passenger*[Title/Abstract] OR pedestrian*[Title/Abstract] OR rider*[Title/Abstract] OR walk*[Title/Abstract] OR traffic*[Title/Abstract] OR transport*[Title/Abstract] OR automobile*[Title/Abstract] OR bus[Title/Abstract] OR buses[Title/Abstract] OR car[Title/Abstract] OR cars[Title/Abstract] OR carpark*[Title/Abstract] OR "dial a ride"[Title/Abstract] OR garage*[Title/Abstract] OR metro[Title/Abstract] OR railway*[Title/Abstract] OR parking*[Title/Abstract] OR sidewalk*[Title/Abstract] OR subway*[Title/Abstract] OR taxi*[Title/Abstract] OR train[Title/Abstract] OR trains[Title/Abstract] OR underground[Title/Abstract] OR vehicle*[Title/Abstract] OR crossing*[Title/Abstract] OR highway*[Title/Abstract] OR lane[Title/Abstract] OR lanes[Title/Abstract] OR road[Title/Abstract] OR roads[Title/Abstract] OR street* [Title/Abstract]) AND (abilit*[Title/Abstract] OR access*[Title/Abstract] OR afford*[Title/Abstract] OR behaviour*[Title/Abstract] OR behavior*[Title/Abstract] OR capacit*[Title/Abstract] OR danger*[Title/Abstract] OR difficult*[Title/Abstract] OR ergonomic*[Title/Abstract] OR habit*[Title/Abstract] OR safe*[Title/Abstract] OR stable[Title/Abstract] OR stability[Title/Abstract] OR unsafe[Title/Abstract] OR ((fare*[Title/Abstract] OR price*[Title/Abstract] OR ticket* [Title/Abstract]) AND (discount*[Title/Abstract] OR free[Title/Abstract] OR reduc* [Title/Abstract])) OR accident*[Title/Abstract] OR collision*[Title/Abstract] OR crash*[Title/Abstract] OR hazard*[Title/Abstract] OR

	incident*[Title/Abstract] OR speed* [Title/Abstract])) OR ((bicycl*[Title/Abstract] OR cycli*[Title/Abstract] OR driv*[Title/Abstract] OR motorcycl*[Title/Abstract] OR automobile*[Title/Abstract] OR bus[Title/Abstract] OR buses[Title/Abstract] OR car[Title/Abstract] OR cars[Title/Abstract] OR metro[Title/Abstract] OR railway*[Title/Abstract] OR subway*[Title/Abstract] OR taxi*[Title/Abstract] OR train[Title/Abstract] OR trains[Title/Abstract] OR underground[Title/Abstract] OR vehicle* [Title/Abstract]) AND (adapt*[Title/Abstract] OR alteration*[Title/Abstract] OR modif*[Title/Abstract] OR support[Title/Abstract] OR usability [Title/Abstract])))
4	(((ageing[Title/Abstract] OR aging[Title/Abstract] OR elder*[Title/Abstract] OR eldest[Title/Abstract] OR geriatric*[Title/Abstract] OR gerontolog*[Title/Abstract] OR age-friendly[Title/Abstract] OR "healthy ageing"[Title/Abstract] OR "active ageing"[Title/Abstract] OR ageing[Title/Abstract] OR aging[Title/Abstract] OR "care home resident*"[Title/Abstract] OR community-dwelling[Title/Abstract] OR elder*[Title/Abstract] OR frail*[Title/Abstract] OR geriatric*[Title/Abstract] OR "late life"[Title/Abstract] OR "later life"[Title/Abstract] OR "mature adult*"[Title/Abstract] OR nonagenarian*[Title/Abstract] OR "nursing home resident*"[Title/Abstract] OR octogenarian*[Title/Abstract] OR "old age"[Title/Abstract] OR "oldest old"[Title/Abstract] OR pensioner*[Title/Abstract] OR postmenopaus*[Title/Abstract] OR post-menopaus*[Title/Abstract] OR retired[Title/Abstract] OR retiree*[Title/Abstract] OR "senior citizen*"[Title/Abstract] OR seniors[Title/Abstract] OR (">=50* years old"[Title/Abstract] OR ">50*

years old"[Title/Abstract] OR ">=60* years old"[Title/Abstract] OR ">60* years old"[Title/Abstract] OR ">=70* years old"[Title/Abstract] OR ">7*0 years old"[Title/Abstract] OR ">=80* years old"[Title/Abstract] OR ">80* years old"[Title/Abstract] OR ">=90* years old"[Title/Abstract] OR ">90* years old" [Title/Abstract]) OR ((older[Title/Abstract] AND adult* [Title/Abstract]) OR "old age*"[Title/Abstract] OR (older[Title/Abstract] AND client* [Title/Abstract]) OR (older[Title/Abstract] AND communit* [Title/Abstract]) OR (older[Title/Abstract] AND female* [Title/Abstract]) OR (older[Title/Abstract] AND individual* [Title/Abstract]) OR (older[Title/Abstract] AND inpatient* [Title/Abstract]) OR (older[Title/Abstract] AND in-patient* [Title/Abstract]) OR (older[Title/Abstract] AND male* [Title/Abstract]) OR (older[Title/Abstract] AND men [Title/Abstract]) OR (older[Title/Abstract] AND outpatient* [Title/Abstract]) OR (older[Title/Abstract] AND out-patient* [Title/Abstract]) OR (older[Title/Abstract] AND patient* [Title/Abstract]) OR (older[Title/Abstract] AND people [Title/Abstract]) OR (older[Title/Abstract] AND person* [Title/Abstract]) OR (older[Title/Abstract] AND population* [Title/Abstract]) OR (older[Title/Abstract] AND women [Title/Abstract])) OR "age in place"[Title/Abstract] OR "aging in place"[Title/Abstract] OR "ageing in place"[Title/Abstract] OR ((active[Title/Abstract] OR healthy [Title/Abstract]) AND (ageing[Title/Abstract] OR aging [Title/Abstract])) OR (friendl*[Title/Abstract] AND (age[Title/Abstract] OR ages[Title/Abstract] OR aged[Title/Abstract] OR aging[Title/Abstract] OR ageing[Title/Abstract] OR elder* [Title/Abstract])))) AND ((systematic*[Title/Abstract] AND review* [Title/Abstract]) OR meta-analy*[Title/Abstract] OR

"meta analy*"[Title/Abstract] OR "evidence map"[Title/Abstract] OR "evidence maps"[Title/Abstract] OR "evidence mapping"[Title/Abstract] OR "evidence and gap map"[Title/Abstract] OR EGM[Title/Abstract] OR "evaluation map"[Title/Abstract] OR "evaluation maps"[Title/Abstract] OR "evaluation mapping"[Title/Abstract] OR "systematic map"[Title/Abstract] OR "systematic maps"[Title/Abstract] OR "systematic mapping"[Title/Abstract] OR "descriptive map"[Title/Abstract] OR "descriptive maps"[Title/Abstract] OR "descriptive mapping"[Title/Abstract])) AND (("active* transport*"[Title/Abstract] OR "active* travel*"[Title/Abstract] OR ((bicycl*[Title/Abstract] OR cycl*[Title/Abstract] OR driv*[Title/Abstract] OR motorcycl*[Title/Abstract] OR walk* [Title/Abstract]) AND (coach*[Title/Abstract] OR course*[Title/Abstract] OR educat*[Title/Abstract] OR instruct*[Title/Abstract] OR lesson*[Title/Abstract] OR school*[Title/Abstract] OR train* [Title/Abstract])) OR ((bicycl*[Title/Abstract] OR cycli*[Title/Abstract] OR driv*[Title/Abstract] OR motorcycl*[Title/Abstract] OR motorist*[Title/Abstract] OR passenger*[Title/Abstract] OR pedestrian*[Title/Abstract] OR rider*[Title/Abstract] OR walk*[Title/Abstract] OR traffic*[Title/Abstract] OR transport*[Title/Abstract] OR automobile*[Title/Abstract] OR bus[Title/Abstract] OR buses[Title/Abstract] OR car[Title/Abstract] OR cars[Title/Abstract] OR carpark*[Title/Abstract] OR "dial a ride"[Title/Abstract] OR garage*[Title/Abstract] OR metro[Title/Abstract] OR railway*[Title/Abstract] OR parking*[Title/Abstract] OR sidewalk*[Title/Abstract] OR subway*[Title/Abstract] OR taxi*[Title/Abstract] OR train[Title/Abstract] OR trains[Title/Abstract] OR

	underground[Title/Abstract] OR vehicle*[Title/Abstract] OR crossing*[Title/Abstract] OR highway*[Title/Abstract] OR lane[Title/Abstract] OR lanes[Title/Abstract] OR road[Title/Abstract] OR roads[Title/Abstract] OR street* [Title/Abstract]) AND (abilit*[Title/Abstract] OR access*[Title/Abstract] OR afford*[Title/Abstract] OR behaviour*[Title/Abstract] OR behavior*[Title/Abstract] OR capacit*[Title/Abstract] OR danger*[Title/Abstract] OR difficult*[Title/Abstract] OR ergonomic*[Title/Abstract] OR habit*[Title/Abstract] OR safe*[Title/Abstract] OR stable[Title/Abstract] OR stability[Title/Abstract] OR unsafe[Title/Abstract] OR ((fare*[Title/Abstract] OR price*[Title/Abstract] OR ticket* [Title/Abstract]) AND (discount*[Title/Abstract] OR free[Title/Abstract] OR reduc* [Title/Abstract])) OR accident*[Title/Abstract] OR collision*[Title/Abstract] OR crash*[Title/Abstract] OR hazard*[Title/Abstract] OR incident*[Title/Abstract] OR speed* [Title/Abstract])) OR ((bicycl*[Title/Abstract] OR cycli*[Title/Abstract] OR driv*[Title/Abstract] OR motorcycl*[Title/Abstract] OR automobile*[Title/Abstract] OR bus[Title/Abstract] OR buses[Title/Abstract] OR car[Title/Abstract] OR cars[Title/Abstract] OR metro[Title/Abstract] OR railway*[Title/Abstract] OR subway*[Title/Abstract] OR taxi*[Title/Abstract] OR train[Title/Abstract] OR trains[Title/Abstract] OR underground[Title/Abstract] OR vehicle* [Title/Abstract]) AND (adapt*[Title/Abstract] OR alteration*[Title/Abstract] OR modif*[Title/Abstract] OR support[Title/Abstract] OR usability [Title/Abstract]))))
A) Age terms	(ageing[Title/Abstract] OR aging[Title/Abstract] OR elder*[Title/Abstract] OR eldest[Title/Abstract] OR geriatric*[Title/Abstract] OR gerontolog*[Title/Abstract] OR age-

friendly[Title/Abstract] OR "healthy ageing"[Title/Abstract] OR "active ageing"[Title/Abstract] OR ageing[Title/Abstract] OR aging[Title/Abstract] OR "care home resident*"[Title/Abstract] OR community-dwelling[Title/Abstract] OR elder*[Title/Abstract] OR frail*[Title/Abstract] OR geriatric*[Title/Abstract] OR "late life"[Title/Abstract] OR "later life"[Title/Abstract] OR "mature adult*"[Title/Abstract] OR nonagenarian*[Title/Abstract] OR "nursing home resident*"[Title/Abstract] OR octogenarian*[Title/Abstract] OR "old age"[Title/Abstract] OR "oldest old"[Title/Abstract] OR pensioner*[Title/Abstract] OR postmenopaus*[Title/Abstract] OR post-menopaus*[Title/Abstract] OR retired[Title/Abstract] OR retiree*[Title/Abstract] OR "senior citizen*"[Title/Abstract] OR seniors[Title/Abstract] OR (">=50* years old"[Title/Abstract] OR ">50* years old"[Title/Abstract] OR ">=60* years old"[Title/Abstract] OR ">60* years old"[Title/Abstract] OR ">=70* years old"[Title/Abstract] OR ">7*0 years old"[Title/Abstract] OR ">=80* years old"[Title/Abstract] OR ">80* years old"[Title/Abstract] OR ">=90* years old"[Title/Abstract] OR ">90* years old" [Title/Abstract]) OR ((older[Title/Abstract] AND adult* [Title/Abstract]) OR "old age*"[Title/Abstract] OR (older[Title/Abstract] AND client* [Title/Abstract]) OR (older[Title/Abstract] AND communit* [Title/Abstract]) OR (older[Title/Abstract] AND female* [Title/Abstract]) OR (older[Title/Abstract] AND individual* [Title/Abstract]) OR (older[Title/Abstract] AND inpatient* [Title/Abstract]) OR (older[Title/Abstract] AND in-patient* [Title/Abstract]) OR (older[Title/Abstract] AND male* [Title/Abstract]) OR (older[Title/Abstract] AND men [Title/Abstract])

		OR (older[Title/Abstract] AND outpatient* [Title/Abstract]) OR (older[Title/Abstract] AND out-patient* [Title/Abstract]) OR (older[Title/Abstract] AND patient* [Title/Abstract]) OR (older[Title/Abstract] AND people [Title/Abstract]) OR (older[Title/Abstract] AND person* [Title/Abstract]) OR (older[Title/Abstract] AND population* [Title/Abstract]) OR (older[Title/Abstract] AND women [Title/Abstract])) OR "age in place"[Title/Abstract] OR "aging in place"[Title/Abstract] OR "ageing in place"[Title/Abstract] OR ((active[Title/Abstract] OR healthy [Title/Abstract]) AND (ageing[Title/Abstract] OR aging [Title/Abstract])) OR (friendl*[Title/Abstract] AND (age[Title/Abstract] OR ages[Title/Abstract] OR aged[Title/Abstract] OR aging[Title/Abstract] OR ageing[Title/Abstract] OR elder* [Title/Abstract])))
B) Methodology term (s)	2	(systematic*[Title/Abstract] AND review* [Title/Abstract]) OR meta-analy*[Title/Abstract] OR "meta analy*"[Title/Abstract] OR "evidence map"[Title/Abstract] OR "evidence maps"[Title/Abstract] OR "evidence mapping"[Title/Abstract] OR "evidence and gap map"[Title/Abstract] OR EGM[Title/Abstract] OR "evaluation map"[Title/Abstract] OR "evaluation maps"[Title/Abstract] OR "evaluation mapping"[Title/Abstract] OR "systematic map"[Title/Abstract] OR "systematic maps"[Title/Abstract] OR "systematic mapping"[Title/Abstract] OR "descriptive map"[Title/Abstract] OR "descriptive maps"[Title/Abstract] OR "descriptive mapping"[Title/Abstract]
C) Intervention terms	3<br><br>Domain 2: Housing	(((ambient*[Title/Abstract] AND assist*[Title/Abstract] AND living [Title/Abstract]) OR ((smart[Title/Abstract] OR automat* [Title/Abstract]) AND home* [Title/Abstract]) OR (smart[Title/Abstract] AND environment* [Title/Abstract]) OR ((home*[Title/Abstract] OR

hous*[Title/Abstract] OR residen*[Title/Abstract] OR accommodation*[Title/Abstract] OR bath*[Title/Abstract] OR bedroom*[Title/Abstract] OR kitchen*[Title/Abstract] OR room*[Title/Abstract] OR shower*[Title/Abstract] OR stair*[Title/Abstract] OR toilet* [Title/Abstract]) AND (access*[Title/Abstract] OR adapt*[Title/Abstract] OR alteration*[Title/Abstract] OR modif*[Title/Abstract] OR renovat*[Title/Abstract] OR support[Title/Abstract] OR usability [Title/Abstract])) OR ((elevator*[Title/Abstract] OR handrail*[Title/Abstract] OR "hand rail*"[Title/Abstract] OR lift[Title/Abstract] OR lifts[Title/Abstract] OR ramp[Title/Abstract] OR ramps[Title/Abstract] OR gerontechnolog*[Title/Abstract] OR "voice assistant*"[Title/Abstract] OR (service[Title/Abstract] AND robot* [Title/Abstract]) OR ((assist*[Title/Abstract] OR intelligen*[Title/Abstract] OR safe*[Title/Abstract] OR self-help*[Title/Abstract] OR welfare* [Title/Abstract]) AND (aid[Title/Abstract] OR aids[Title/Abstract] OR device*[Title/Abstract] OR platform*[Title/Abstract] OR robot*[Title/Abstract] OR solution*[Title/Abstract] OR technolog* [Title/Abstract]))) AND (home*[Title/Abstract] OR hous*[Title/Abstract] OR residen*[Title/Abstract] OR accommodation*[Title/Abstract] OR city[Title/Abstract] OR cities[Title/Abstract] OR communit*[Title/Abstract] OR environment*[Title/Abstract] OR neighb*[Title/Abstract] OR rural[Title/Abstract] OR societ*[Title/Abstract] OR town*[Title/Abstract] OR urban[Title/Abstract] OR village* [Title/Abstract]))))
OR ("naturally occurring retirement communi*"[Title/Abstract] OR NORC[Title/Abstract] OR housing[Title/Abstract] OR cohousi*[Title/Abstract] OR co-hous*[Title/Abstract] OR "university based

retirement communi*"[Title/Abstract] OR UBRC*[Title/Abstract] OR apartment*[Title/Abstract] OR bungalow*[Title/Abstract] OR dwelling*[Title/Abstract] OR housing[Title/Abstract] OR landlord*[Title/Abstract] OR rehous*[Title/Abstract] OR squatter*[Title/Abstract] OR tenant*[Title/Abstract] OR bedsit[Title/Abstract] OR bedsitting[Title/Abstract] OR "bed sit"[Title/Abstract] OR "bed sitting"[Title/Abstract] OR "residential care"[Title/Abstract] OR highrise*[Title/Abstract] OR "high rise*"[Title/Abstract] OR homeowner*[Title/Abstract] OR "home owner*"[Title/Abstract] OR "indoor air qualit*"[Title/Abstract] OR "living environment*"[Title/Abstract] OR "live environment*or "built environment""[Title/Abstract] OR "living quarter*"[Title/Abstract] OR multistor*[Title/Abstract] OR "multi stor*"[Title/Abstract] OR "owner* occup*"[Title/Abstract] OR towerblock*[Title/Abstract] OR "tower block*"[Title/Abstract] OR cottage*[Title/Abstract] OR flat*[Title/Abstract] OR houses[Title/Abstract] OR home[Title/Abstract] OR stair[Title/Abstract] OR staircase[Title/Abstract] OR stairwell[Title/Abstract] OR maisonette*[Title/Abstract] OR indoor*[Title/Abstract])

4 (((ageing[Title/Abstract] OR aging[Title/Abstract] OR elder*[Title/Abstract] OR eldest[Title/Abstract] OR geriatric*[Title/Abstract] OR gerontolog*[Title/Abstract] OR age-friendly[Title/Abstract] OR "healthy ageing"[Title/Abstract] OR "active ageing"[Title/Abstract] OR ageing[Title/Abstract] OR aging[Title/Abstract] OR "care home resident*"[Title/Abstract] OR community-dwelling[Title/Abstract] OR elder*[Title/Abstract] OR frail*[Title/Abstract] OR

geriatric*[Title/Abstract] OR "late life"[Title/Abstract] OR "later life"[Title/Abstract] OR "mature adult*"[Title/Abstract] OR nonagenarian*[Title/Abstract] OR "nursing home resident*"[Title/Abstract] OR octogenarian*[Title/Abstract] OR "old age"[Title/Abstract] OR "oldest old"[Title/Abstract] OR pensioner*[Title/Abstract] OR postmenopaus*[Title/Abstract] OR post-menopaus*[Title/Abstract] OR retired[Title/Abstract] OR retiree*[Title/Abstract] OR "senior citizen*"[Title/Abstract] OR seniors[Title/Abstract] OR (">=50* years old"[Title/Abstract] OR ">50* years old"[Title/Abstract] OR ">=60* years old"[Title/Abstract] OR ">60* years old"[Title/Abstract] OR ">=70* years old"[Title/Abstract] OR ">7*0 years old"[Title/Abstract] OR ">=80* years old"[Title/Abstract] OR ">80* years old"[Title/Abstract] OR ">=90* years old"[Title/Abstract] OR ">90* years old" [Title/Abstract]) OR ((older[Title/Abstract] AND adult* [Title/Abstract]) OR "old age*"[Title/Abstract] OR (older[Title/Abstract] AND client* [Title/Abstract]) OR (older[Title/Abstract] AND communit* [Title/Abstract]) OR (older[Title/Abstract] AND female* [Title/Abstract]) OR (older[Title/Abstract] AND individual* [Title/Abstract]) OR (older[Title/Abstract] AND inpatient* [Title/Abstract]) OR (older[Title/Abstract] AND in-patient* [Title/Abstract]) OR (older[Title/Abstract] AND male* [Title/Abstract]) OR (older[Title/Abstract] AND men [Title/Abstract]) OR (older[Title/Abstract] AND outpatient* [Title/Abstract]) OR (older[Title/Abstract] AND out-patient* [Title/Abstract]) OR (older[Title/Abstract] AND patient* [Title/Abstract]) OR (older[Title/Abstract] AND people [Title/Abstract]) OR (older[Title/Abstract] AND person* [Title/Abstract]) OR (older[Title/Abstract] AND

population* [Title/Abstract]) OR (older[Title/Abstract] AND women [Title/Abstract])) OR "age in place"[Title/Abstract] OR "aging in place"[Title/Abstract] OR "ageing in place"[Title/Abstract] OR ((active[Title/Abstract] OR healthy [Title/Abstract]) AND (ageing[Title/Abstract] OR aging [Title/Abstract])) OR (friendl*[Title/Abstract] AND (age[Title/Abstract] OR ages[Title/Abstract] OR aged[Title/Abstract] OR aging[Title/Abstract] OR ageing[Title/Abstract] OR elder* [Title/Abstract])))) AND ((systematic*[Title/Abstract] AND review* [Title/Abstract]) OR meta-analy*[Title/Abstract] OR "meta analy*"[Title/Abstract] OR "evidence map"[Title/Abstract] OR "evidence maps"[Title/Abstract] OR "evidence mapping"[Title/Abstract] OR "evidence and gap map"[Title/Abstract] OR EGM[Title/Abstract] OR "evaluation map"[Title/Abstract] OR "evaluation maps"[Title/Abstract] OR "evaluation mapping"[Title/Abstract] OR "systematic map"[Title/Abstract] OR "systematic maps"[Title/Abstract] OR "systematic mapping"[Title/Abstract] OR "descriptive map"[Title/Abstract] OR "descriptive maps"[Title/Abstract] OR "descriptive mapping"[Title/Abstract])) AND ((((ambient*[Title/Abstract] AND assist*[Title/Abstract] AND living [Title/Abstract]) OR ((smart[Title/Abstract] OR automat* [Title/Abstract]) AND home* [Title/Abstract]) OR (smart[Title/Abstract] AND environment* [Title/Abstract]) OR ((home*[Title/Abstract] OR hous*[Title/Abstract] OR residen*[Title/Abstract] OR accommodation*[Title/Abstract] OR bath*[Title/Abstract] OR bedroom*[Title/Abstract] OR kitchen*[Title/Abstract] OR room*[Title/Abstract] OR shower*[Title/Abstract] OR stair*[Title/Abstract] OR toilet* [Title/Abstract]) AND (access*[Title/Abstract] OR

adapt*[Title/Abstract] OR alteration*[Title/Abstract] OR modif*[Title/Abstract] OR renovat*[Title/Abstract] OR support[Title/Abstract] OR usability [Title/Abstract])) OR ((elevator*[Title/Abstract] OR handrail*[Title/Abstract] OR "hand rail*"[Title/Abstract] OR lift[Title/Abstract] OR lifts[Title/Abstract] OR ramp[Title/Abstract] OR ramps[Title/Abstract] OR gerontechnolog*[Title/Abstract] OR "voice assistant*"[Title/Abstract] OR (service[Title/Abstract] AND robot* [Title/Abstract]) OR ((assist*[Title/Abstract] OR intelligen*[Title/Abstract] OR safe*[Title/Abstract] OR self-help*[Title/Abstract] OR welfare* [Title/Abstract]) AND (aid[Title/Abstract] OR aids[Title/Abstract] OR device*[Title/Abstract] OR platform*[Title/Abstract] OR robot*[Title/Abstract] OR solution*[Title/Abstract] OR technolog* [Title/Abstract]))) AND (home*[Title/Abstract] OR hous*[Title/Abstract] OR residen*[Title/Abstract] OR accommodation*[Title/Abstract] OR city[Title/Abstract] OR cities[Title/Abstract] OR communit*[Title/Abstract] OR environment*[Title/Abstract] OR neighb*[Title/Abstract] OR rural[Title/Abstract] OR societ*[Title/Abstract] OR town*[Title/Abstract] OR urban[Title/Abstract] OR village* [Title/Abstract]))) OR ("naturally occurring retirement communi*"[Title/Abstract] OR NORC[Title/Abstract] OR housing[Title/Abstract] OR cohousi*[Title/Abstract] OR co-hous*[Title/Abstract] OR "university based retirement communi*"[Title/Abstract] OR UBRC*[Title/Abstract] OR apartment*[Title/Abstract] OR bungalow*[Title/Abstract] OR dwelling*[Title/Abstract] OR housing[Title/Abstract] OR landlord*[Title/Abstract] OR rehous*[Title/Abstract] OR squatter*[Title/Abstract]

		OR tenant*[Title/Abstract] OR bedsit[Title/Abstract] OR bedsitting[Title/Abstract] OR "bed sit"[Title/Abstract] OR "bed sitting"[Title/Abstract] OR "residential care"[Title/Abstract] OR highrise*[Title/Abstract] OR "high rise*"[Title/Abstract] OR homeowner*[Title/Abstract] OR "home owner*"[Title/Abstract] OR "indoor air qualit*"[Title/Abstract] OR "living environment*"[Title/Abstract] OR "live environment*or "built environment""[Title/Abstract] OR "living quarter*"[Title/Abstract] OR multistor*[Title/Abstract] OR "multi stor*"[Title/Abstract] OR "owner* occup*"[Title/Abstract] OR towerblock*[Title/Abstract] OR "tower block*"[Title/Abstract] OR cottage*[Title/Abstract] OR flat*[Title/Abstract] OR houses[Title/Abstract] OR home[Title/Abstract] OR stair[Title/Abstract] OR staircase[Title/Abstract] OR stairwell[Title/Abstract] OR maisonette*[Title/Abstract] OR indoor*[Title/Abstract]))
A) Age terms	1	(ageing[Title/Abstract] OR aging[Title/Abstract] OR elder*[Title/Abstract] OR eldest[Title/Abstract] OR geriatric*[Title/Abstract] OR gerontolog*[Title/Abstract] OR age-friendly[Title/Abstract] OR "healthy ageing"[Title/Abstract] OR "active ageing"[Title/Abstract] OR ageing[Title/Abstract] OR aging[Title/Abstract] OR "care home resident*"[Title/Abstract] OR community-dwelling[Title/Abstract] OR elder*[Title/Abstract] OR frail*[Title/Abstract] OR geriatric*[Title/Abstract] OR "late life"[Title/Abstract] OR "later life"[Title/Abstract] OR "mature adult*"[Title/Abstract] OR nonagenarian*[Title/Abstract] OR "nursing home resident*"[Title/Abstract] OR octogenarian*[Title/Abstract] OR "old age"[Title/Abstract] OR "oldest old"[Title/Abstract]

OR pensioner*[Title/Abstract] OR postmenopaus*[Title/Abstract] OR post-menopaus*[Title/Abstract] OR retired[Title/Abstract] OR retiree*[Title/Abstract] OR "senior citizen*"[Title/Abstract] OR seniors[Title/Abstract] OR (">=50* years old"[Title/Abstract] OR ">50* years old"[Title/Abstract] OR ">=60* years old"[Title/Abstract] OR ">60* years old"[Title/Abstract] OR ">=70* years old"[Title/Abstract] OR ">7*0 years old"[Title/Abstract] OR ">=80* years old"[Title/Abstract] OR ">80* years old"[Title/Abstract] OR ">=90* years old"[Title/Abstract] OR ">90* years old" [Title/Abstract]) OR ((older[Title/Abstract] AND adult* [Title/Abstract]) OR "old age*"[Title/Abstract] OR (older[Title/Abstract] AND client* [Title/Abstract]) OR (older[Title/Abstract] AND communit* [Title/Abstract]) OR (older[Title/Abstract] AND female* [Title/Abstract]) OR (older[Title/Abstract] AND individual* [Title/Abstract]) OR (older[Title/Abstract] AND inpatient* [Title/Abstract]) OR (older[Title/Abstract] AND in-patient* [Title/Abstract]) OR (older[Title/Abstract] AND male* [Title/Abstract]) OR (older[Title/Abstract] AND men [Title/Abstract]) OR (older[Title/Abstract] AND outpatient* [Title/Abstract]) OR (older[Title/Abstract] AND out-patient* [Title/Abstract]) OR (older[Title/Abstract] AND patient* [Title/Abstract]) OR (older[Title/Abstract] AND people [Title/Abstract]) OR (older[Title/Abstract] AND person* [Title/Abstract]) OR (older[Title/Abstract] AND population* [Title/Abstract]) OR (older[Title/Abstract] AND women [Title/Abstract])) OR "age in place"[Title/Abstract] OR "aging in place"[Title/Abstract] OR "ageing in place"[Title/Abstract] OR ((active[Title/Abstract] OR healthy [Title/Abstract]) AND (ageing[Title/Abstract] OR aging [Title/Abstract]))

		OR (friendl*[Title/Abstract] AND (age[Title/Abstract] OR ages[Title/Abstract] OR aged[Title/Abstract] OR aging[Title/Abstract] OR ageing[Title/Abstract] OR elder* [Title/Abstract])))
B) Methodology term (s)	2	(systematic*[Title/Abstract] AND review* [Title/Abstract]) OR meta-analy*[Title/Abstract] OR "meta analy*"[Title/Abstract] OR "evidence map"[Title/Abstract] OR "evidence maps"[Title/Abstract] OR "evidence mapping"[Title/Abstract] OR "evidence and gap map"[Title/Abstract] OR EGM[Title/Abstract] OR "evaluation map"[Title/Abstract] OR "evaluation maps"[Title/Abstract] OR "evaluation mapping"[Title/Abstract] OR "systematic map"[Title/Abstract] OR "systematic maps"[Title/Abstract] OR "systematic mapping"[Title/Abstract] OR "descriptive map"[Title/Abstract] OR "descriptive maps"[Title/Abstract] OR "descriptive mapping"[Title/Abstract]
C) Intervention terms	3<br><br>Domain 3: Social participation	"social participat*"[Title/Abstract] OR "community involve*"[Title/Abstract] OR community-dwelling[Title/Abstract] OR "civic engage*"[Title/Abstract] OR "active citizen*"[Title/Abstract] OR "social involve*"[Title/Abstract] OR "community participat*"[Title/Abstract] OR "public engag*"[Title/Abstract] OR "societal engage*"[Title/Abstract] OR "participatory citizenship"[Title/Abstract] OR "active participat*"[Title/Abstract] OR "collective action"[Title/Abstract] OR "engage* in society"[Title/Abstract] OR "group participat*"[Title/Abstract] OR "collaborat* involve*"[Title/Abstract] OR "social integrat*"[Title/Abstract] OR "involve* in public life"[Title/Abstract] OR "social connect*"[Title/Abstract] OR "social health"[Title/Abstract] OR "social capital"[Title/Abstract]

4	(((ageing[Title/Abstract] OR aging[Title/Abstract] OR elder*[Title/Abstract] OR eldest[Title/Abstract] OR geriatric*[Title/Abstract] OR gerontolog*[Title/Abstract] OR age-friendly[Title/Abstract] OR "healthy ageing"[Title/Abstract] OR "active ageing"[Title/Abstract] OR ageing[Title/Abstract] OR aging[Title/Abstract] OR "care home resident*"[Title/Abstract] OR community-dwelling[Title/Abstract] OR elder*[Title/Abstract] OR frail*[Title/Abstract] OR geriatric*[Title/Abstract] OR "late life"[Title/Abstract] OR "later life"[Title/Abstract] OR "mature adult*"[Title/Abstract] OR nonagenarian*[Title/Abstract] OR "nursing home resident*"[Title/Abstract] OR octogenarian*[Title/Abstract] OR "old age"[Title/Abstract] OR "oldest old"[Title/Abstract] OR pensioner*[Title/Abstract] OR postmenopaus*[Title/Abstract] OR post-menopaus*[Title/Abstract] OR retired[Title/Abstract] OR retiree*[Title/Abstract] OR "senior citizen*"[Title/Abstract] OR seniors[Title/Abstract] OR (">=50* years old"[Title/Abstract] OR ">50* years old"[Title/Abstract] OR ">=60* years old"[Title/Abstract] OR ">60* years old"[Title/Abstract] OR ">=70* years old"[Title/Abstract] OR ">7*0 years old"[Title/Abstract] OR ">=80* years old"[Title/Abstract] OR ">80* years old"[Title/Abstract] OR ">=90* years old"[Title/Abstract] OR ">90* years old" [Title/Abstract]) OR ((older[Title/Abstract] AND adult* [Title/Abstract]) OR "old age*"[Title/Abstract] OR (older[Title/Abstract] AND client* [Title/Abstract]) OR (older[Title/Abstract] AND communit* [Title/Abstract]) OR (older[Title/Abstract] AND female* [Title/Abstract]) OR (older[Title/Abstract] AND individual* [Title/Abstract]) OR (older[Title/Abstract] AND

inpatient* [Title/Abstract]) OR (older[Title/Abstract] AND in-patient* [Title/Abstract]) OR (older[Title/Abstract] AND male* [Title/Abstract]) OR (older[Title/Abstract] AND men [Title/Abstract]) OR (older[Title/Abstract] AND outpatient* [Title/Abstract]) OR (older[Title/Abstract] AND out-patient* [Title/Abstract]) OR (older[Title/Abstract] AND patient* [Title/Abstract]) OR (older[Title/Abstract] AND people [Title/Abstract]) OR (older[Title/Abstract] AND person* [Title/Abstract]) OR (older[Title/Abstract] AND population* [Title/Abstract]) OR (older[Title/Abstract] AND women [Title/Abstract])) OR "age in place"[Title/Abstract] OR "aging in place"[Title/Abstract] OR "ageing in place"[Title/Abstract] OR ((active[Title/Abstract] OR healthy [Title/Abstract]) AND (ageing[Title/Abstract] OR aging [Title/Abstract])) OR (friendl*[Title/Abstract] AND (age[Title/Abstract] OR ages[Title/Abstract] OR aged[Title/Abstract] OR aging[Title/Abstract] OR ageing[Title/Abstract] OR elder* [Title/Abstract])))) AND ((systematic*[Title/Abstract] AND review* [Title/Abstract]) OR meta-analy*[Title/Abstract] OR "meta analy*"[Title/Abstract] OR "evidence map"[Title/Abstract] OR "evidence maps"[Title/Abstract] OR "evidence mapping"[Title/Abstract] OR "evidence and gap map"[Title/Abstract] OR EGM[Title/Abstract] OR "evaluation map"[Title/Abstract] OR "evaluation maps"[Title/Abstract] OR "evaluation mapping"[Title/Abstract] OR "systematic map"[Title/Abstract] OR "systematic maps"[Title/Abstract] OR "systematic mapping"[Title/Abstract] OR "descriptive map"[Title/Abstract] OR "descriptive maps"[Title/Abstract] OR "descriptive mapping"[Title/Abstract])) AND ("social participat*"[Title/Abstract] OR "community involve*"[Title/Abstract] OR community-

		dwelling[Title/Abstract] OR "civic engage*"[Title/Abstract] OR "active citizen*"[Title/Abstract] OR "social involve*"[Title/Abstract] OR "community participat*"[Title/Abstract] OR "public engag*"[Title/Abstract] OR "societal engage*"[Title/Abstract] OR "participatory citizenship"[Title/Abstract] OR "active participat*"[Title/Abstract] OR "collective action"[Title/Abstract] OR "engage* in society"[Title/Abstract] OR "group participat*"[Title/Abstract] OR "collaborat* involve*"[Title/Abstract] OR "social integrat*"[Title/Abstract] OR "involve* in public life"[Title/Abstract] OR "social connect*"[Title/Abstract] OR "social health"[Title/Abstract] OR "social capital"[Title/Abstract])
A) Age terms	1	(ageing[Title/Abstract] OR aging[Title/Abstract] OR elder*[Title/Abstract] OR eldest[Title/Abstract] OR geriatric*[Title/Abstract] OR gerontolog*[Title/Abstract] OR age-friendly[Title/Abstract] OR "healthy ageing"[Title/Abstract] OR "active ageing"[Title/Abstract] OR ageing[Title/Abstract] OR aging[Title/Abstract] OR "care home resident*"[Title/Abstract] OR community-dwelling[Title/Abstract] OR elder*[Title/Abstract] OR frail*[Title/Abstract] OR geriatric*[Title/Abstract] OR "late life"[Title/Abstract] OR "later life"[Title/Abstract] OR "mature adult*"[Title/Abstract] OR nonagenarian*[Title/Abstract] OR "nursing home resident*"[Title/Abstract] OR octogenarian*[Title/Abstract] OR "old age"[Title/Abstract] OR "oldest old"[Title/Abstract] OR pensioner*[Title/Abstract] OR postmenopaus*[Title/Abstract] OR post-menopaus*[Title/Abstract] OR retired[Title/Abstract] OR retiree*[Title/Abstract] OR "senior

citizen*"[Title/Abstract] OR seniors[Title/Abstract] OR (">=50* years old"[Title/Abstract] OR ">50* years old"[Title/Abstract] OR ">=60* years old"[Title/Abstract] OR ">60* years old"[Title/Abstract] OR ">=70* years old"[Title/Abstract] OR ">7*0 years old"[Title/Abstract] OR ">=80* years old"[Title/Abstract] OR ">80* years old"[Title/Abstract] OR ">=90* years old"[Title/Abstract] OR ">90* years old" [Title/Abstract]) OR ((older[Title/Abstract] AND adult* [Title/Abstract]) OR "old age*"[Title/Abstract] OR (older[Title/Abstract] AND client* [Title/Abstract]) OR (older[Title/Abstract] AND communit* [Title/Abstract]) OR (older[Title/Abstract] AND female* [Title/Abstract]) OR (older[Title/Abstract] AND individual* [Title/Abstract]) OR (older[Title/Abstract] AND inpatient* [Title/Abstract]) OR (older[Title/Abstract] AND in-patient* [Title/Abstract]) OR (older[Title/Abstract] AND male* [Title/Abstract]) OR (older[Title/Abstract] AND men [Title/Abstract]) OR (older[Title/Abstract] AND outpatient* [Title/Abstract]) OR (older[Title/Abstract] AND out-patient* [Title/Abstract]) OR (older[Title/Abstract] AND patient* [Title/Abstract]) OR (older[Title/Abstract] AND people [Title/Abstract]) OR (older[Title/Abstract] AND person* [Title/Abstract]) OR (older[Title/Abstract] AND population* [Title/Abstract]) OR (older[Title/Abstract] AND women [Title/Abstract])) OR "age in place"[Title/Abstract] OR "aging in place"[Title/Abstract] OR "ageing in place"[Title/Abstract] OR ((active[Title/Abstract] OR healthy [Title/Abstract]) AND (ageing[Title/Abstract] OR aging [Title/Abstract])) OR (friendl*[Title/Abstract] AND (age[Title/Abstract] OR ages[Title/Abstract] OR aged[Title/Abstract] OR aging[Title/Abstract] OR ageing[Title/Abstract] OR elder* [Title/Abstract])))

A) Methodology term (s)	2	(systematic*[Title/Abstract] AND review*[Title/Abstract]) OR meta-analy*[Title/Abstract] OR "meta analy*"[Title/Abstract] OR "evidence map"[Title/Abstract] OR "evidence maps"[Title/Abstract] OR "evidence mapping"[Title/Abstract] OR "evidence and gap map"[Title/Abstract] OR EGM[Title/Abstract] OR "evaluation map"[Title/Abstract] OR "evaluation maps"[Title/Abstract] OR "evaluation mapping"[Title/Abstract] OR "systematic map"[Title/Abstract] OR "systematic maps"[Title/Abstract] OR "systematic mapping"[Title/Abstract] OR "descriptive map"[Title/Abstract] OR "descriptive maps"[Title/Abstract] OR "descriptive mapping"[Title/Abstract]
A) Intervention terms	3<br><br>Domain 4: Respect and social inclusion	"social inclusion"[Title/Abstract] OR "community inclusion"[Title/Abstract] OR e-inclusion[Title/Abstract] OR "digital inclusion"[Title/Abstract] OR "social cohesion"[Title/Abstract] OR "community cohesion"[Title/Abstract] OR "neighbo* cohesion"[Title/Abstract] OR "social involvement"[Title/Abstract] OR "community involvement"[Title/Abstract] OR "social integration"[Title/Abstract] OR "community integration"[Title/Abstract] OR "social engagement"[Title/Abstract] OR "civic engagement"[Title/Abstract] OR "community engagement"[Title/Abstract] OR intergeneration*[Title/Abstract] OR "social recognition"[Title/Abstract] OR "information and communication technolog*"[Title/Abstract] OR "social exclusion"[Title/Abstract] OR "neighbo* inclusion"[Title/Abstract] OR "neighbo* exclusion"[Title/Abstract] OR "community participation"[Title/Abstract] OR "social participation"[Title/Abstract] OR ageism[Title/Abstract] OR agism[Title/Abstract] OR "age stereotyp*"[Title/Abstract] OR "ageing

	stereotyp*"[Title/Abstract] OR "aging stereotyp*"[Title/Abstract] OR "age discrimination"[Title/Abstract] OR "ageing discrimination"[Title/Abstract] OR "aging discrimination"[Title/Abstract] OR "digital divide"[Title/Abstract] OR "social interaction*"[Title/Abstract] OR "social responsabilit*"[Title/Abstract] OR "social capital"[Title/Abstract] OR "social networks"[Title/Abstract] OR "access services"[Title/Abstract] OR "access information"[Title/Abstract] OR "access opportunit*"[Title/Abstract] OR "access faciliti*"[Title/Abstract] OR "access volunteer*"[Title/Abstract] OR "access learning"[Title/Abstract] OR "social exchange*"[Title/Abstract] OR solidarity[Title/Abstract]
4	(((ageing[Title/Abstract] OR aging[Title/Abstract] OR elder*[Title/Abstract] OR eldest[Title/Abstract] OR geriatric*[Title/Abstract] OR gerontolog*[Title/Abstract] OR age-friendly[Title/Abstract] OR "healthy ageing"[Title/Abstract] OR "active ageing"[Title/Abstract] OR ageing[Title/Abstract] OR aging[Title/Abstract] OR "care home resident*"[Title/Abstract] OR community-dwelling[Title/Abstract] OR elder*[Title/Abstract] OR frail*[Title/Abstract] OR geriatric*[Title/Abstract] OR "late life"[Title/Abstract] OR "later life"[Title/Abstract] OR "mature adult*"[Title/Abstract] OR nonagenarian*[Title/Abstract] OR "nursing home resident*"[Title/Abstract] OR octogenarian*[Title/Abstract] OR "old age"[Title/Abstract] OR "oldest old"[Title/Abstract] OR pensioner*[Title/Abstract] OR postmenopaus*[Title/Abstract] OR post-menopaus*[Title/Abstract] OR retired[Title/Abstract] OR retiree*[Title/Abstract] OR "senior

citizen*"[Title/Abstract] OR seniors[Title/Abstract] OR (">=50* years old"[Title/Abstract] OR ">50* years old"[Title/Abstract] OR ">=60* years old"[Title/Abstract] OR ">60* years old"[Title/Abstract] OR ">=70* years old"[Title/Abstract] OR ">7*0 years old"[Title/Abstract] OR ">=80* years old"[Title/Abstract] OR ">80* years old"[Title/Abstract] OR ">=90* years old"[Title/Abstract] OR ">90* years old" [Title/Abstract]) OR ((older[Title/Abstract] AND adult* [Title/Abstract]) OR "old age*"[Title/Abstract] OR (older[Title/Abstract] AND client* [Title/Abstract]) OR (older[Title/Abstract] AND communit* [Title/Abstract]) OR (older[Title/Abstract] AND female* [Title/Abstract]) OR (older[Title/Abstract] AND individual* [Title/Abstract]) OR (older[Title/Abstract] AND inpatient* [Title/Abstract]) OR (older[Title/Abstract] AND in-patient* [Title/Abstract]) OR (older[Title/Abstract] AND male* [Title/Abstract]) OR (older[Title/Abstract] AND men [Title/Abstract]) OR (older[Title/Abstract] AND outpatient* [Title/Abstract]) OR (older[Title/Abstract] AND out-patient* [Title/Abstract]) OR (older[Title/Abstract] AND patient* [Title/Abstract]) OR (older[Title/Abstract] AND people [Title/Abstract]) OR (older[Title/Abstract] AND person* [Title/Abstract]) OR (older[Title/Abstract] AND population* [Title/Abstract]) OR (older[Title/Abstract] AND women [Title/Abstract])) OR "age in place"[Title/Abstract] OR "aging in place"[Title/Abstract] OR "ageing in place"[Title/Abstract] OR ((active[Title/Abstract] OR healthy [Title/Abstract]) AND (ageing[Title/Abstract] OR aging [Title/Abstract])) OR (friendl*[Title/Abstract] AND (age[Title/Abstract] OR ages[Title/Abstract] OR aged[Title/Abstract] OR aging[Title/Abstract] OR ageing[Title/Abstract] OR elder* [Title/Abstract]))))

AND ((systematic*[Title/Abstract] AND review* [Title/Abstract]) OR meta-analy*[Title/Abstract] OR "meta analy*"[Title/Abstract] OR "evidence map"[Title/Abstract] OR "evidence maps"[Title/Abstract] OR "evidence mapping"[Title/Abstract] OR "evidence and gap map"[Title/Abstract] OR EGM[Title/Abstract] OR "evaluation map"[Title/Abstract] OR "evaluation maps"[Title/Abstract] OR "evaluation mapping"[Title/Abstract] OR "systematic map"[Title/Abstract] OR "systematic maps"[Title/Abstract] OR "systematic mapping"[Title/Abstract] OR "descriptive map"[Title/Abstract] OR "descriptive maps"[Title/Abstract] OR "descriptive mapping"[Title/Abstract])) AND ("social inclusion"[Title/Abstract] OR "community inclusion"[Title/Abstract] OR e-inclusion[Title/Abstract] OR "digital inclusion"[Title/Abstract] OR "social cohesion"[Title/Abstract] OR "community cohesion"[Title/Abstract] OR "neighbo* cohesion"[Title/Abstract] OR "social involvement"[Title/Abstract] OR "community involvement"[Title/Abstract] OR "social integration"[Title/Abstract] OR "community integration"[Title/Abstract] OR "social engagement"[Title/Abstract] OR "civic engagement"[Title/Abstract] OR "community engagement"[Title/Abstract] OR intergeneration*[Title/Abstract] OR "social recognition"[Title/Abstract] OR "information and communication technolog*"[Title/Abstract] OR "social exclusion"[Title/Abstract] OR "neighbo* inclusion"[Title/Abstract] OR "neighbo* exclusion"[Title/Abstract] OR "community participation"[Title/Abstract] OR "social participation"[Title/Abstract] OR ageism[Title/Abstract] OR agism[Title/Abstract] OR "age stereotyp*"[Title/Abstract] OR "ageing

		stereotyp*"[Title/Abstract] OR "aging stereotyp*"[Title/Abstract] OR "age discrimination"[Title/Abstract] OR "ageing discrimination"[Title/Abstract] OR "aging discrimination"[Title/Abstract] OR "digital divide"[Title/Abstract] OR "social interaction*"[Title/Abstract] OR "social responsabilit*"[Title/Abstract] OR "social capital"[Title/Abstract] OR "social networks"[Title/Abstract] OR "access services"[Title/Abstract] OR "access information"[Title/Abstract] OR "access opportunit*"[Title/Abstract] OR "access faciliti*"[Title/Abstract] OR "access volunteer*"[Title/Abstract] OR "access learning"[Title/Abstract] OR "social exchange*"[Title/Abstract] OR solidarity[Title/Abstract])
A) Age terms	1	(ageing[Title/Abstract] OR aging[Title/Abstract] OR elder*[Title/Abstract] OR eldest[Title/Abstract] OR geriatric*[Title/Abstract] OR gerontolog*[Title/Abstract] OR age-friendly[Title/Abstract] OR "healthy ageing"[Title/Abstract] OR "active ageing"[Title/Abstract] OR ageing[Title/Abstract] OR aging[Title/Abstract] OR "care home resident*"[Title/Abstract] OR community-dwelling[Title/Abstract] OR elder*[Title/Abstract] OR frail*[Title/Abstract] OR geriatric*[Title/Abstract] OR "late life"[Title/Abstract] OR "later life"[Title/Abstract] OR "mature adult*"[Title/Abstract] OR nonagenarian*[Title/Abstract] OR "nursing home resident*"[Title/Abstract] OR octogenarian*[Title/Abstract] OR "old age"[Title/Abstract] OR "oldest old"[Title/Abstract] OR pensioner*[Title/Abstract] OR postmenopaus*[Title/Abstract] OR post-menopaus*[Title/Abstract] OR retired[Title/Abstract] OR retiree*[Title/Abstract] OR "senior

citizen*"[Title/Abstract] OR seniors[Title/Abstract]
OR (">=50* years old"[Title/Abstract] OR ">50*
years old"[Title/Abstract] OR ">=60* years
old"[Title/Abstract] OR ">60* years
old"[Title/Abstract] OR ">=70* years
old"[Title/Abstract] OR ">7*0 years
old"[Title/Abstract] OR ">=80* years
old"[Title/Abstract] OR ">80* years
old"[Title/Abstract] OR ">=90* years
old"[Title/Abstract] OR ">90* years old"
[Title/Abstract]) OR ((older[Title/Abstract] AND
adult* [Title/Abstract]) OR "old
age*"[Title/Abstract] OR (older[Title/Abstract] AND
client* [Title/Abstract]) OR (older[Title/Abstract]
AND communit* [Title/Abstract]) OR
(older[Title/Abstract] AND female* [Title/Abstract])
OR (older[Title/Abstract] AND individual*
[Title/Abstract]) OR (older[Title/Abstract] AND
inpatient* [Title/Abstract]) OR (older[Title/Abstract]
AND in-patient* [Title/Abstract]) OR
(older[Title/Abstract] AND male* [Title/Abstract])
OR (older[Title/Abstract] AND men [Title/Abstract])
OR (older[Title/Abstract] AND outpatient*
[Title/Abstract]) OR (older[Title/Abstract] AND out-
patient* [Title/Abstract]) OR (older[Title/Abstract]
AND patient* [Title/Abstract]) OR
(older[Title/Abstract] AND people [Title/Abstract])
OR (older[Title/Abstract] AND person*
[Title/Abstract]) OR (older[Title/Abstract] AND
population* [Title/Abstract]) OR
(older[Title/Abstract] AND women [Title/Abstract]))
OR "age in place"[Title/Abstract] OR "aging in
place"[Title/Abstract] OR "ageing in
place"[Title/Abstract] OR ((active[Title/Abstract] OR
healthy [Title/Abstract]) AND
(ageing[Title/Abstract] OR aging [Title/Abstract]))
OR (friendl*[Title/Abstract] AND
(age[Title/Abstract] OR ages[Title/Abstract] OR
aged[Title/Abstract] OR aging[Title/Abstract] OR
ageing[Title/Abstract] OR elder* [Title/Abstract])))

B) Methodology term (s)	2	(systematic*[Title/Abstract] AND review* [Title/Abstract]) OR meta-analy*[Title/Abstract] OR "meta analy*"[Title/Abstract] OR "evidence map"[Title/Abstract] OR "evidence maps"[Title/Abstract] OR "evidence mapping"[Title/Abstract] OR "evidence and gap map"[Title/Abstract] OR EGM[Title/Abstract] OR "evaluation map"[Title/Abstract] OR "evaluation maps"[Title/Abstract] OR "evaluation mapping"[Title/Abstract] OR "systematic map"[Title/Abstract] OR "systematic maps"[Title/Abstract] OR "systematic mapping"[Title/Abstract] OR "descriptive map"[Title/Abstract] OR "descriptive maps"[Title/Abstract] OR "descriptive mapping"[Title/Abstract]
C) Interventions terms	3<br><br>Domain 5: Civic participation and employment	"civil participation"[Title/Abstract] OR "citizen participation"[Title/Abstract] OR "community engagement"[Title/Abstract] OR "public participation"[Title/Abstract] OR "civic engagement"[Title/Abstract] OR "political participation"[Title/Abstract] OR employ*[Title/Abstract] OR job[Title/Abstract] OR occupation[Title/Abstract] OR work*[Title/Abstract] OR career[Title/Abstract] OR "labour market"[Title/Abstract] OR "labor market"[Title/Abstract] OR "job market"[Title/Abstract] OR workforce[Title/Abstract] OR independence[Title/Abstract]
	4	(((ageing[Title/Abstract] OR aging[Title/Abstract] OR elder*[Title/Abstract] OR eldest[Title/Abstract] OR geriatric*[Title/Abstract] OR gerontolog*[Title/Abstract] OR age-friendly[Title/Abstract] OR "healthy ageing"[Title/Abstract] OR "active ageing"[Title/Abstract] OR ageing[Title/Abstract] OR aging[Title/Abstract] OR "care home resident*"[Title/Abstract] OR community-dwelling[Title/Abstract] OR elder*[Title/Abstract]

OR frail*[Title/Abstract] OR geriatric*[Title/Abstract] OR "late life"[Title/Abstract] OR "later life"[Title/Abstract] OR "mature adult*"[Title/Abstract] OR nonagenarian*[Title/Abstract] OR "nursing home resident*"[Title/Abstract] OR octogenarian*[Title/Abstract] OR "old age"[Title/Abstract] OR "oldest old"[Title/Abstract] OR pensioner*[Title/Abstract] OR postmenopaus*[Title/Abstract] OR post-menopaus*[Title/Abstract] OR retired[Title/Abstract] OR retiree*[Title/Abstract] OR "senior citizen*"[Title/Abstract] OR seniors[Title/Abstract] OR (">=50* years old"[Title/Abstract] OR ">50* years old"[Title/Abstract] OR ">=60* years old"[Title/Abstract] OR ">60* years old"[Title/Abstract] OR ">=70* years old"[Title/Abstract] OR ">7*0 years old"[Title/Abstract] OR ">=80* years old"[Title/Abstract] OR ">80* years old"[Title/Abstract] OR ">=90* years old"[Title/Abstract] OR ">90* years old" [Title/Abstract]) OR ((older[Title/Abstract] AND adult* [Title/Abstract]) OR "old age*"[Title/Abstract] OR (older[Title/Abstract] AND client* [Title/Abstract]) OR (older[Title/Abstract] AND communit* [Title/Abstract]) OR (older[Title/Abstract] AND female* [Title/Abstract]) OR (older[Title/Abstract] AND individual* [Title/Abstract]) OR (older[Title/Abstract] AND inpatient* [Title/Abstract]) OR (older[Title/Abstract] AND in-patient* [Title/Abstract]) OR (older[Title/Abstract] AND male* [Title/Abstract]) OR (older[Title/Abstract] AND men [Title/Abstract]) OR (older[Title/Abstract] AND outpatient* [Title/Abstract]) OR (older[Title/Abstract] AND out-patient* [Title/Abstract]) OR (older[Title/Abstract] AND patient* [Title/Abstract]) OR (older[Title/Abstract] AND people [Title/Abstract]) OR (older[Title/Abstract] AND person*

[Title/Abstract]) OR (older[Title/Abstract] AND population* [Title/Abstract]) OR (older[Title/Abstract] AND women [Title/Abstract])) OR "age in place"[Title/Abstract] OR "aging in place"[Title/Abstract] OR "ageing in place"[Title/Abstract] OR ((active[Title/Abstract] OR healthy [Title/Abstract]) AND (ageing[Title/Abstract] OR aging [Title/Abstract])) OR (friendl*[Title/Abstract] AND (age[Title/Abstract] OR ages[Title/Abstract] OR aged[Title/Abstract] OR aging[Title/Abstract] OR ageing[Title/Abstract] OR elder* [Title/Abstract])))) AND ((systematic*[Title/Abstract] AND review* [Title/Abstract]) OR meta-analy*[Title/Abstract] OR "meta analy*"[Title/Abstract] OR "evidence map"[Title/Abstract] OR "evidence maps"[Title/Abstract] OR "evidence mapping"[Title/Abstract] OR "evidence and gap map"[Title/Abstract] OR EGM[Title/Abstract] OR "evaluation map"[Title/Abstract] OR "evaluation maps"[Title/Abstract] OR "evaluation mapping"[Title/Abstract] OR "systematic map"[Title/Abstract] OR "systematic maps"[Title/Abstract] OR "systematic mapping"[Title/Abstract] OR "descriptive map"[Title/Abstract] OR "descriptive maps"[Title/Abstract] OR "descriptive mapping"[Title/Abstract])) AND ("civil participation"[Title/Abstract] OR "citizen participation"[Title/Abstract] OR "community engagement"[Title/Abstract] OR "public participation"[Title/Abstract] OR "civic engagement"[Title/Abstract] OR "political participation"[Title/Abstract] OR employ*[Title/Abstract] OR job[Title/Abstract] OR occupation[Title/Abstract] OR work*[Title/Abstract] OR career[Title/Abstract] OR "labour market"[Title/Abstract] OR "labor market"[Title/Abstract] OR "job market"[Title/Abstract] OR

		workforce[Title/Abstract] OR independence[Title/Abstract])
A) Age terms	1	(ageing[Title/Abstract] OR aging[Title/Abstract] OR elder*[Title/Abstract] OR eldest[Title/Abstract] OR geriatric*[Title/Abstract] OR gerontolog*[Title/Abstract] OR age-friendly[Title/Abstract] OR "healthy ageing"[Title/Abstract] OR "active ageing"[Title/Abstract] OR ageing[Title/Abstract] OR aging[Title/Abstract] OR "care home resident*"[Title/Abstract] OR community-dwelling[Title/Abstract] OR elder*[Title/Abstract] OR frail*[Title/Abstract] OR geriatric*[Title/Abstract] OR "late life"[Title/Abstract] OR "later life"[Title/Abstract] OR "mature adult*"[Title/Abstract] OR nonagenarian*[Title/Abstract] OR "nursing home resident*"[Title/Abstract] OR octogenarian*[Title/Abstract] OR "old age"[Title/Abstract] OR "oldest old"[Title/Abstract] OR pensioner*[Title/Abstract] OR postmenopaus*[Title/Abstract] OR post-menopaus*[Title/Abstract] OR retired[Title/Abstract] OR retiree*[Title/Abstract] OR "senior citizen*"[Title/Abstract] OR seniors[Title/Abstract] OR (">=50* years old"[Title/Abstract] OR ">50* years old"[Title/Abstract] OR ">=60* years old"[Title/Abstract] OR ">60* years old"[Title/Abstract] OR ">=70* years old"[Title/Abstract] OR ">7*0 years old"[Title/Abstract] OR ">=80* years old"[Title/Abstract] OR ">80* years old"[Title/Abstract] OR ">=90* years old"[Title/Abstract] OR ">90* years old" [Title/Abstract]) OR ((older[Title/Abstract] AND adult* [Title/Abstract]) OR "old age*"[Title/Abstract] OR (older[Title/Abstract] AND client* [Title/Abstract]) OR (older[Title/Abstract] AND communit* [Title/Abstract]) OR (older[Title/Abstract] AND female* [Title/Abstract])

		OR (older[Title/Abstract] AND individual* [Title/Abstract]) OR (older[Title/Abstract] AND inpatient* [Title/Abstract]) OR (older[Title/Abstract] AND in-patient* [Title/Abstract]) OR (older[Title/Abstract] AND male* [Title/Abstract]) OR (older[Title/Abstract] AND men [Title/Abstract]) OR (older[Title/Abstract] AND outpatient* [Title/Abstract]) OR (older[Title/Abstract] AND out-patient* [Title/Abstract]) OR (older[Title/Abstract] AND patient* [Title/Abstract]) OR (older[Title/Abstract] AND people [Title/Abstract]) OR (older[Title/Abstract] AND person* [Title/Abstract]) OR (older[Title/Abstract] AND population* [Title/Abstract]) OR (older[Title/Abstract] AND women [Title/Abstract])) OR "age in place"[Title/Abstract] OR "aging in place"[Title/Abstract] OR "ageing in place"[Title/Abstract] OR ((active[Title/Abstract] OR healthy [Title/Abstract]) AND (ageing[Title/Abstract] OR aging [Title/Abstract])) OR (friendl*[Title/Abstract] AND (age[Title/Abstract] OR ages[Title/Abstract] OR aged[Title/Abstract] OR aging[Title/Abstract] OR ageing[Title/Abstract] OR elder* [Title/Abstract])))
B) Methodology terms	2	(systematic*[Title/Abstract] AND review* [Title/Abstract]) OR meta-analy*[Title/Abstract] OR "meta analy*"[Title/Abstract] OR "evidence map"[Title/Abstract] OR "evidence maps"[Title/Abstract] OR "evidence mapping"[Title/Abstract] OR "evidence and gap map"[Title/Abstract] OR EGM[Title/Abstract] OR "evaluation map"[Title/Abstract] OR "evaluation maps"[Title/Abstract] OR "evaluation mapping"[Title/Abstract] OR "systematic map"[Title/Abstract] OR "systematic maps"[Title/Abstract] OR "systematic mapping"[Title/Abstract] OR "descriptive map"[Title/Abstract] OR "descriptive maps"[Title/Abstract] OR "descriptive mapping"[Title/Abstract]

C) Interventions terms	3<br><br>Domain 6: Communication and information	communication [Title/Abstract] OR information [Title/Abstract] OR negot*[Title/Abstract] OR counselling [Title/Abstract] OR learning [Title/Abstract] OR literacy [Title/Abstract] OR training [Title/Abstract] OR coaching [Title/Abstract]
	4	(((ageing[Title/Abstract] OR aging[Title/Abstract] OR elder*[Title/Abstract] OR eldest[Title/Abstract] OR geriatric*[Title/Abstract] OR gerontolog*[Title/Abstract] OR age-friendly[Title/Abstract] OR "healthy ageing"[Title/Abstract] OR "active ageing"[Title/Abstract] OR ageing[Title/Abstract] OR aging[Title/Abstract] OR "care home resident*"[Title/Abstract] OR community-dwelling[Title/Abstract] OR elder*[Title/Abstract] OR frail*[Title/Abstract] OR geriatric*[Title/Abstract] OR "late life"[Title/Abstract] OR "later life"[Title/Abstract] OR "mature adult*"[Title/Abstract] OR nonagenarian*[Title/Abstract] OR "nursing home resident*"[Title/Abstract] OR octogenarian*[Title/Abstract] OR "old age"[Title/Abstract] OR "oldest old"[Title/Abstract] OR pensioner*[Title/Abstract] OR postmenopaus*[Title/Abstract] OR post-menopaus*[Title/Abstract] OR retired[Title/Abstract] OR retiree*[Title/Abstract] OR "senior citizen*"[Title/Abstract] OR seniors[Title/Abstract] OR (">=50* years old"[Title/Abstract] OR ">50* years old"[Title/Abstract] OR ">=60* years old"[Title/Abstract] OR ">60* years old"[Title/Abstract] OR ">=70* years old"[Title/Abstract] OR ">7*0 years old"[Title/Abstract] OR ">=80* years old"[Title/Abstract] OR ">80* years old"[Title/Abstract] OR ">=90* years old"[Title/Abstract] OR ">90* years old" [Title/Abstract]) OR ((older[Title/Abstract] AND adult* [Title/Abstract]) OR "old

age*"[Title/Abstract] OR (older[Title/Abstract] AND client* [Title/Abstract]) OR (older[Title/Abstract] AND communit* [Title/Abstract]) OR (older[Title/Abstract] AND female* [Title/Abstract]) OR (older[Title/Abstract] AND individual* [Title/Abstract]) OR (older[Title/Abstract] AND inpatient* [Title/Abstract]) OR (older[Title/Abstract] AND in-patient* [Title/Abstract]) OR (older[Title/Abstract] AND male* [Title/Abstract]) OR (older[Title/Abstract] AND men [Title/Abstract]) OR (older[Title/Abstract] AND outpatient* [Title/Abstract]) OR (older[Title/Abstract] AND out-patient* [Title/Abstract]) OR (older[Title/Abstract] AND patient* [Title/Abstract]) OR (older[Title/Abstract] AND people [Title/Abstract]) OR (older[Title/Abstract] AND person* [Title/Abstract]) OR (older[Title/Abstract] AND population* [Title/Abstract]) OR (older[Title/Abstract] AND women [Title/Abstract])) OR "age in place"[Title/Abstract] OR "aging in place"[Title/Abstract] OR "ageing in place"[Title/Abstract] OR ((active[Title/Abstract] OR healthy [Title/Abstract]) AND (ageing[Title/Abstract] OR aging [Title/Abstract])) OR (friendl*[Title/Abstract] AND (age[Title/Abstract] OR ages[Title/Abstract] OR aged[Title/Abstract] OR aging[Title/Abstract] OR ageing[Title/Abstract] OR elder* [Title/Abstract])))) AND ((systematic*[Title/Abstract] AND review* [Title/Abstract]) OR meta-analy*[Title/Abstract] OR "meta analy*"[Title/Abstract] OR "evidence map"[Title/Abstract] OR "evidence maps"[Title/Abstract] OR "evidence mapping"[Title/Abstract] OR "evidence and gap map"[Title/Abstract] OR EGM[Title/Abstract] OR "evaluation map"[Title/Abstract] OR "evaluation maps"[Title/Abstract] OR "evaluation mapping"[Title/Abstract] OR "systematic map"[Title/Abstract] OR "systematic maps"[Title/Abstract] OR "systematic

		mapping"[Title/Abstract] OR "descriptive map"[Title/Abstract] OR "descriptive maps"[Title/Abstract] OR "descriptive mapping"[Title/Abstract])) AND (communication[Title/Abstract] OR information[Title/Abstract] OR negot*[Title/Abstract] OR counselling[Title/Abstract] OR learning[Title/Abstract] OR literacy[Title/Abstract] OR training[Title/Abstract] OR coaching[Title/Abstract])
A) Age terms	1	(ageing[Title/Abstract] OR aging[Title/Abstract] OR elder*[Title/Abstract] OR eldest[Title/Abstract] OR geriatric*[Title/Abstract] OR gerontolog*[Title/Abstract] OR age-friendly[Title/Abstract] OR "healthy ageing"[Title/Abstract] OR "active ageing"[Title/Abstract] OR ageing[Title/Abstract] OR aging[Title/Abstract] OR "care home resident*"[Title/Abstract] OR community-dwelling[Title/Abstract] OR elder*[Title/Abstract] OR frail*[Title/Abstract] OR geriatric*[Title/Abstract] OR "late life"[Title/Abstract] OR "later life"[Title/Abstract] OR "mature adult*"[Title/Abstract] OR nonagenarian*[Title/Abstract] OR "nursing home resident*"[Title/Abstract] OR octogenarian*[Title/Abstract] OR "old age"[Title/Abstract] OR "oldest old"[Title/Abstract] OR pensioner*[Title/Abstract] OR postmenopaus*[Title/Abstract] OR post-menopaus*[Title/Abstract] OR retired[Title/Abstract] OR retiree*[Title/Abstract] OR "senior citizen*"[Title/Abstract] OR seniors[Title/Abstract] OR (">=50* years old"[Title/Abstract] OR ">50* years old"[Title/Abstract] OR ">=60* years old"[Title/Abstract] OR ">60* years old"[Title/Abstract] OR ">=70* years old"[Title/Abstract] OR ">7*0 years old"[Title/Abstract] OR ">=80* years

		old"[Title/Abstract] OR ">80* years old"[Title/Abstract] OR ">=90* years old"[Title/Abstract] OR ">90* years old" [Title/Abstract]) OR ((older[Title/Abstract] AND adult* [Title/Abstract]) OR "old age*"[Title/Abstract] OR (older[Title/Abstract] AND client* [Title/Abstract]) OR (older[Title/Abstract] AND communit* [Title/Abstract]) OR (older[Title/Abstract] AND female* [Title/Abstract]) OR (older[Title/Abstract] AND individual* [Title/Abstract]) OR (older[Title/Abstract] AND inpatient* [Title/Abstract]) OR (older[Title/Abstract] AND in-patient* [Title/Abstract]) OR (older[Title/Abstract] AND male* [Title/Abstract]) OR (older[Title/Abstract] AND men [Title/Abstract]) OR (older[Title/Abstract] AND outpatient* [Title/Abstract]) OR (older[Title/Abstract] AND out-patient* [Title/Abstract]) OR (older[Title/Abstract] AND patient* [Title/Abstract]) OR (older[Title/Abstract] AND people [Title/Abstract]) OR (older[Title/Abstract] AND person* [Title/Abstract]) OR (older[Title/Abstract] AND population* [Title/Abstract]) OR (older[Title/Abstract] AND women [Title/Abstract])) OR "age in place"[Title/Abstract] OR "aging in place"[Title/Abstract] OR "ageing in place"[Title/Abstract] OR ((active[Title/Abstract] OR healthy [Title/Abstract]) AND (ageing[Title/Abstract] OR aging [Title/Abstract])) OR (friendl*[Title/Abstract] AND (age[Title/Abstract] OR ages[Title/Abstract] OR aged[Title/Abstract] OR aging[Title/Abstract] OR ageing[Title/Abstract] OR elder* [Title/Abstract])))
B) Methodology term (s)	2	(systematic*[Title/Abstract] AND review* [Title/Abstract]) OR meta-analy*[Title/Abstract] OR "meta analy*"[Title/Abstract] OR "evidence map"[Title/Abstract] OR "evidence maps"[Title/Abstract] OR "evidence mapping"[Title/Abstract] OR "evidence and gap map"[Title/Abstract] OR EGM[Title/Abstract] OR

		"evaluation map"[Title/Abstract] OR "evaluation maps"[Title/Abstract] OR "evaluation mapping"[Title/Abstract] OR "systematic map"[Title/Abstract] OR "systematic maps"[Title/Abstract] OR "systematic mapping"[Title/Abstract] OR "descriptive map"[Title/Abstract] OR "descriptive maps"[Title/Abstract] OR "descriptive mapping"[Title/Abstract]
C) Interventions terms	3<br><br>Domain 7: Community support and health services	"community support"[Title/Abstract] OR "community assist*"[Title/Abstract] OR "community aid"[Title/Abstract] OR "local support"[Title/Abstract] OR "social support"[Title/Abstract] OR "community resource*"[Title/Abstract] OR "community service*"[Title/Abstract] OR "mutual aid"[Title/Abstract] OR "community welfare"[Title/Abstract] OR "community development"[Title/Abstract] OR "grassroot* support"[Title/Abstract] OR "volunteer support"[Title/Abstract] OR "community engagement*"[Title/Abstract] OR "community involvement*"[Title/Abstract] OR "health service*"[Title/Abstract] OR "healthcare service*"[Title/Abstract] OR "medical service*"[Title/Abstract] OR "healthcare facility*"[Title/Abstract] OR "healthcare system*"[Title/Abstract] OR "healthcare delivery"[Title/Abstract] OR "healthcare access"[Title/Abstract] OR "healthcare utilisation"[Title/Abstract] OR "healthcare utilization"[Title/Abstract] OR "healthcare management"[Title/Abstract] OR "healthcare policy"[Title/Abstract] OR "healthcare administration"[Title/Abstract] OR "healthcare quality"[Title/Abstract] OR "healthcare reform"[Title/Abstract] OR "assisted living"[Title/Abstract] OR AL[Title/Abstract] OR "retirement home*"[Title/Abstract] OR "retirement communit*"[Title/Abstract] OR "supportive

	hous*"[Title/Abstract] OR "long-term care"[Title/Abstract] OR "senior care"[Title/Abstract] OR "aged care"[Title/Abstract]
4	(((ageing[Title/Abstract] OR aging[Title/Abstract] OR elder*[Title/Abstract] OR eldest[Title/Abstract] OR geriatric*[Title/Abstract] OR gerontolog*[Title/Abstract] OR age-friendly[Title/Abstract] OR "healthy ageing"[Title/Abstract] OR "active ageing"[Title/Abstract] OR ageing[Title/Abstract] OR aging[Title/Abstract] OR "care home resident*"[Title/Abstract] OR community-dwelling[Title/Abstract] OR elder*[Title/Abstract] OR frail*[Title/Abstract] OR geriatric*[Title/Abstract] OR "late life"[Title/Abstract] OR "later life"[Title/Abstract] OR "mature adult*"[Title/Abstract] OR nonagenarian*[Title/Abstract] OR "nursing home resident*"[Title/Abstract] OR octogenarian*[Title/Abstract] OR "old age"[Title/Abstract] OR "oldest old"[Title/Abstract] OR pensioner*[Title/Abstract] OR postmenopaus*[Title/Abstract] OR post-menopaus*[Title/Abstract] OR retired[Title/Abstract] OR retiree*[Title/Abstract] OR "senior citizen*"[Title/Abstract] OR seniors[Title/Abstract] OR (">=50* years old"[Title/Abstract] OR ">50* years old"[Title/Abstract] OR ">=60* years old"[Title/Abstract] OR ">60* years old"[Title/Abstract] OR ">=70* years old"[Title/Abstract] OR ">7*0 years old"[Title/Abstract] OR ">=80* years old"[Title/Abstract] OR ">80* years old"[Title/Abstract] OR ">=90* years old"[Title/Abstract] OR ">90* years old" [Title/Abstract]) OR ((older[Title/Abstract] AND adult* [Title/Abstract]) OR "old age*"[Title/Abstract] OR (older[Title/Abstract] AND client* [Title/Abstract]) OR (older[Title/Abstract] AND communit* [Title/Abstract]) OR

(older[Title/Abstract] AND female* [Title/Abstract]) OR (older[Title/Abstract] AND individual* [Title/Abstract]) OR (older[Title/Abstract] AND inpatient* [Title/Abstract]) OR (older[Title/Abstract] AND in-patient* [Title/Abstract]) OR (older[Title/Abstract] AND male* [Title/Abstract]) OR (older[Title/Abstract] AND men [Title/Abstract]) OR (older[Title/Abstract] AND outpatient* [Title/Abstract]) OR (older[Title/Abstract] AND out-patient* [Title/Abstract]) OR (older[Title/Abstract] AND patient* [Title/Abstract]) OR (older[Title/Abstract] AND people [Title/Abstract]) OR (older[Title/Abstract] AND person* [Title/Abstract]) OR (older[Title/Abstract] AND population* [Title/Abstract]) OR (older[Title/Abstract] AND women [Title/Abstract])) OR "age in place"[Title/Abstract] OR "aging in place"[Title/Abstract] OR "ageing in place"[Title/Abstract] OR ((active[Title/Abstract] OR healthy [Title/Abstract]) AND (ageing[Title/Abstract] OR aging [Title/Abstract])) OR (friendl*[Title/Abstract] AND (age[Title/Abstract] OR ages[Title/Abstract] OR aged[Title/Abstract] OR aging[Title/Abstract] OR ageing[Title/Abstract] OR elder* [Title/Abstract])))) AND ((systematic*[Title/Abstract] AND review* [Title/Abstract]) OR meta-analy*[Title/Abstract] OR "meta analy*"[Title/Abstract] OR "evidence map"[Title/Abstract] OR "evidence maps"[Title/Abstract] OR "evidence mapping"[Title/Abstract] OR "evidence and gap map"[Title/Abstract] OR EGM[Title/Abstract] OR "evaluation map"[Title/Abstract] OR "evaluation maps"[Title/Abstract] OR "evaluation mapping"[Title/Abstract] OR "systematic map"[Title/Abstract] OR "systematic maps"[Title/Abstract] OR "systematic mapping"[Title/Abstract] OR "descriptive map"[Title/Abstract] OR "descriptive maps"[Title/Abstract] OR "descriptive

		mapping"[Title/Abstract])) AND ("community support"[Title/Abstract] OR "community assist*"[Title/Abstract] OR "community aid"[Title/Abstract] OR "local support"[Title/Abstract] OR "social support"[Title/Abstract] OR "community resource*"[Title/Abstract] OR "community service*"[Title/Abstract] OR "mutual aid"[Title/Abstract] OR "community welfare"[Title/Abstract] OR "community development"[Title/Abstract] OR "grassroot* support"[Title/Abstract] OR "volunteer support"[Title/Abstract] OR "community engagement*"[Title/Abstract] OR "community involvement*"[Title/Abstract] OR "health service*"[Title/Abstract] OR "healthcare service*"[Title/Abstract] OR "medical service*"[Title/Abstract] OR "healthcare facility*"[Title/Abstract] OR "healthcare system*"[Title/Abstract] OR "healthcare delivery"[Title/Abstract] OR "healthcare access"[Title/Abstract] OR "healthcare utilisation"[Title/Abstract] OR "healthcare utilization"[Title/Abstract] OR "healthcare management"[Title/Abstract] OR "healthcare policy"[Title/Abstract] OR "healthcare administration"[Title/Abstract] OR "healthcare quality"[Title/Abstract] OR "healthcare reform"[Title/Abstract] OR "assisted living"[Title/Abstract] OR AL[Title/Abstract] OR "retirement home*"[Title/Abstract] OR "retirement communit*"[Title/Abstract] OR "supportive hous*"[Title/Abstract] OR "long-term care"[Title/Abstract] OR "senior care"[Title/Abstract] OR "aged care"[Title/Abstract])
A) Age terms	1	(ageing[Title/Abstract] OR aging[Title/Abstract] OR elder*[Title/Abstract] OR eldest[Title/Abstract] OR geriatric*[Title/Abstract] OR gerontolog*[Title/Abstract] OR age-friendly[Title/Abstract] OR "healthy

ageing"[Title/Abstract] OR "active
ageing"[Title/Abstract] OR ageing[Title/Abstract]
OR aging[Title/Abstract] OR "care home
resident*"[Title/Abstract] OR community-
dwelling[Title/Abstract] OR elder*[Title/Abstract]
OR frail*[Title/Abstract] OR
geriatric*[Title/Abstract] OR "late
life"[Title/Abstract] OR "later life"[Title/Abstract]
OR "mature adult*"[Title/Abstract] OR
nonagenarian*[Title/Abstract] OR "nursing home
resident*"[Title/Abstract] OR
octogenarian*[Title/Abstract] OR "old
age"[Title/Abstract] OR "oldest old"[Title/Abstract]
OR pensioner*[Title/Abstract] OR
postmenopaus*[Title/Abstract] OR post-
menopaus*[Title/Abstract] OR retired[Title/Abstract]
OR retiree*[Title/Abstract] OR "senior
citizen*"[Title/Abstract] OR seniors[Title/Abstract]
OR (">=50* years old"[Title/Abstract] OR ">50*
years old"[Title/Abstract] OR ">=60* years
old"[Title/Abstract] OR ">60* years
old"[Title/Abstract] OR ">=70* years
old"[Title/Abstract] OR ">7*0 years
old"[Title/Abstract] OR ">=80* years
old"[Title/Abstract] OR ">80* years
old"[Title/Abstract] OR ">=90* years
old"[Title/Abstract] OR ">90* years old"
[Title/Abstract]) OR ((older[Title/Abstract] AND
adult* [Title/Abstract]) OR "old
age*"[Title/Abstract] OR (older[Title/Abstract] AND
client* [Title/Abstract]) OR (older[Title/Abstract]
AND communit* [Title/Abstract]) OR
(older[Title/Abstract] AND female* [Title/Abstract])
OR (older[Title/Abstract] AND individual*
[Title/Abstract]) OR (older[Title/Abstract] AND
inpatient* [Title/Abstract]) OR (older[Title/Abstract]
AND in-patient* [Title/Abstract]) OR
(older[Title/Abstract] AND male* [Title/Abstract])
OR (older[Title/Abstract] AND men [Title/Abstract])
OR (older[Title/Abstract] AND outpatient*

		[Title/Abstract]) OR (older[Title/Abstract] AND out-patient* [Title/Abstract]) OR (older[Title/Abstract] AND patient* [Title/Abstract]) OR (older[Title/Abstract] AND people [Title/Abstract]) OR (older[Title/Abstract] AND person* [Title/Abstract]) OR (older[Title/Abstract] AND population* [Title/Abstract]) OR (older[Title/Abstract] AND women [Title/Abstract])) OR "age in place"[Title/Abstract] OR "aging in place"[Title/Abstract] OR "ageing in place"[Title/Abstract] OR ((active[Title/Abstract] OR healthy [Title/Abstract]) AND (ageing[Title/Abstract] OR aging [Title/Abstract])) OR (friendl*[Title/Abstract] AND (age[Title/Abstract] OR ages[Title/Abstract] OR aged[Title/Abstract] OR aging[Title/Abstract] OR ageing[Title/Abstract] OR elder* [Title/Abstract])))
B) Methodology term (s)	2	(systematic*[Title/Abstract] AND review* [Title/Abstract]) OR meta-analy*[Title/Abstract] OR "meta analy*"[Title/Abstract] OR "evidence map"[Title/Abstract] OR "evidence maps"[Title/Abstract] OR "evidence mapping"[Title/Abstract] OR "evidence and gap map"[Title/Abstract] OR EGM[Title/Abstract] OR "evaluation map"[Title/Abstract] OR "evaluation maps"[Title/Abstract] OR "evaluation mapping"[Title/Abstract] OR "systematic map"[Title/Abstract] OR "systematic maps"[Title/Abstract] OR "systematic mapping"[Title/Abstract] OR "descriptive map"[Title/Abstract] OR "descriptive maps"[Title/Abstract] OR "descriptive mapping"[Title/Abstract]
C) Interventions terms	3<br><br>Domain 8: Outdoor space and buildings	(((urban[Title/Abstract] OR rural [Title/Abstract]) AND (plan*[Title/Abstract] OR develop*[Title/Abstract] OR design* [Title/Abstract])) OR ((built[Title/Abstract] OR green[Title/Abstract] OR neighb*[Title/Abstract] OR street*[Title/Abstract] OR rural[Title/Abstract] OR urban[Title/Abstract] OR cycling[Title/Abstract] OR

driving[Title/Abstract] OR walking [Title/Abstract]) AND environment* [Title/Abstract]) OR ((neighb*[Title/Abstract] OR rural[Title/Abstract] OR urban [Title/Abstract]) AND space* [Title/Abstract]) OR amenit*[Title/Abstract] OR "communit* environment*"[Title/Abstract] OR greener*[Title/Abstract] OR greenness[Title/Abstract] OR greenspace*[Title/Abstract] OR greenway*[Title/Abstract] OR ((green[Title/Abstract] OR blue [Title/Abstract]) AND (area*[Title/Abstract] OR space* [Title/Abstract])) OR "land use*"[Title/Abstract] OR streetscape*[Title/Abstract] OR (street*[Title/Abstract] AND connect* [Title/Abstract]) OR "rural renewal"[Title/Abstract] OR "urban renewal"[Title/Abstract] OR "rural form*"[Title/Abstract] OR "urban form*"[Title/Abstract] OR walkabilit*[Title/Abstract] OR ((population[Title/Abstract] OR residential[Title/Abstract] OR retail[Title/Abstract] OR rural[Title/Abstract] OR urban [Title/Abstract]) AND densit* [Title/Abstract]) OR ((aesthetic*[Title/Abstract] OR garden[Title/Abstract] OR gardens[Title/Abstract] OR park[Title/Abstract] OR parks[Title/Abstract] OR ((plan*[Title/Abstract] OR develop*[Title/Abstract] OR design* [Title/Abstract]) AND (facility[Title/Abstract] OR facilities [Title/Abstract])) OR ((outdoor*[Title/Abstract] OR public [Title/Abstract]) AND (space*[Title/Abstract] OR building*[Title/Abstract] OR facility[Title/Abstract] OR facilities* [Title/Abstract])) OR ((indoor*[Title/Abstract] OR living[Title/Abstract] OR natural[Title/Abstract] OR objective[Title/Abstract] OR outdoor*[Title/Abstract] OR perceived[Title/Abstract] OR physical[Title/Abstract] OR urban[Title/Abstract] OR rural [Title/Abstract])

AND environment* [Title/Abstract])) AND (home*[Title/Abstract] OR hous*[Title/Abstract] OR residen*[Title/Abstract] OR accommodation*[Title/Abstract] OR city[Title/Abstract] OR cities[Title/Abstract] OR communit*[Title/Abstract] OR neighb*[Title/Abstract] OR rural[Title/Abstract] OR societ*[Title/Abstract] OR town*[Title/Abstract] OR urban[Title/Abstract] OR village* [Title/Abstract])) OR ((neighb*[Title/Abstract] AND (space*[Title/Abstract] OR building*[Title/Abstract] OR facility[Title/Abstract] OR facilities [Title/Abstract])) AND (home*[Title/Abstract] OR hous*[Title/Abstract] OR residen*[Title/Abstract] OR accommodation*[Title/Abstract] OR city[Title/Abstract] OR cities[Title/Abstract] OR communit*[Title/Abstract] OR rural[Title/Abstract] OR societ*[Title/Abstract] OR town*[Title/Abstract] OR urban[Title/Abstract] OR village* [Title/Abstract])) OR ((communit*[Title/Abstract] AND (space*[Title/Abstract] OR building*[Title/Abstract] OR facility[Title/Abstract] OR facilities [Title/Abstract])) AND (home*[Title/Abstract] OR hous*[Title/Abstract] OR residen*[Title/Abstract] OR city[Title/Abstract] OR cities[Title/Abstract] OR neighb*[Title/Abstract] OR rural[Title/Abstract] OR societ*[Title/Abstract] OR town*[Title/Abstract] OR urban[Title/Abstract] OR village* [Title/Abstract])) OR (infrastructure*[Title/Abstract] AND (city[Title/Abstract] OR cities[Title/Abstract] OR communit*[Title/Abstract] OR cycling[Title/Abstract] OR driving[Title/Abstract] OR neighb*[Title/Abstract] OR pedestrian[Title/Abstract] OR public[Title/Abstract] OR rural[Title/Abstract] OR town*[Title/Abstract] OR transport*[Title/Abstract] OR urban[Title/Abstract] OR village* [Title/Abstract]))) OR (((emission*[Title/Abstract] OR air[Title/Abstract] OR "particulate

matter"[Title/Abstract] OR "ambient particulate"[Title/Abstract] OR "ultrafine particulate*"[Title/Abstract] OR "ultrafine particle*"[Title/Abstract] OR UFP [Title/Abstract]) AND (control*[Title/Abstract] OR regulation*[Title/Abstract] OR policy[Title/Abstract] OR policies[Title/Abstract] OR guideline[Title/Abstract] OR intervention[Title/Abstract] OR act[Title/Abstract] OR directive*[Title/Abstract] OR vehicle[Title/Abstract] OR transport*[Title/Abstract] OR traffic[Title/Abstract] OR automobile*[Title/Abstract] OR car[Title/Abstract] OR cars[Title/Abstract] OR industr*[Title/Abstract] OR fuel[Title/Abstract] OR "emission filter*"[Title/Abstract] OR cooking[Title/Abstract] OR heating[Title/Abstract] OR cookstove*[Title/Abstract] OR stove*[Title/Abstract] OR "power generat*"[Title/Abstract] OR zone*[Title/Abstract] OR Olympic[Title/Abstract] OR residential[Title/Abstract] OR "wood burning"[Title/Abstract] OR mobile[Title/Abstract] OR Low[Title/Abstract] OR Lower[Title/Abstract] OR Lowered[Title/Abstract] OR reduc*[Title/Abstract] OR improv*[Title/Abstract] OR clean*[Title/Abstract] OR congestion*[Title/Abstract] OR "coal burning"[Title/Abstract] OR ban[Title/Abstract] OR bans [Title/Abstract])) OR ((improved[Title/Abstract] OR clean*[Title/Abstract] OR "low emission"[Title/Abstract] OR efficient* [Title/Abstract]) AND (cookstove*[Title/Abstract] OR stove[Title/Abstract] OR stoves[Title/Abstract] OR heater [Title/Abstract]))) OR ((air[Title/Abstract] AND (pollut*[Title/Abstract] OR quality[Title/Abstract] OR ambient [Title/Abstract])) OR (atmospher*[Title/Abstract] AND pollut* [Title/Abstract]) OR ("particulate matter"[Title/Abstract] OR "ambient particulate"[Title/Abstract] OR "ultrafine

	particulate*"[Title/Abstract] OR "ultrafine particle*"[Title/Abstract] OR UFP [Title/Abstract]) OR ("coarse particle*"[Title/Abstract] OR "black smoke"[Title/Abstract] OR "black carbon"[Title/Abstract] OR "elemental carbon"[Title/Abstract] OR "wood smoke" [Title/Abstract]))
4	Search: (((ageing[Title/Abstract] OR aging[Title/Abstract] OR elder*[Title/Abstract] OR eldest[Title/Abstract] OR geriatric*[Title/Abstract] OR gerontolog*[Title/Abstract] OR age-friendly[Title/Abstract] OR "healthy ageing"[Title/Abstract] OR "active ageing"[Title/Abstract] OR ageing[Title/Abstract] OR aging[Title/Abstract] OR "care home resident*"[Title/Abstract] OR community-dwelling[Title/Abstract] OR elder*[Title/Abstract] OR frail*[Title/Abstract] OR geriatric*[Title/Abstract] OR "late life"[Title/Abstract] OR "later life"[Title/Abstract] OR "mature adult*"[Title/Abstract] OR nonagenarian*[Title/Abstract] OR "nursing home resident*"[Title/Abstract] OR octogenarian*[Title/Abstract] OR "old age"[Title/Abstract] OR "oldest old"[Title/Abstract] OR pensioner*[Title/Abstract] OR postmenopaus*[Title/Abstract] OR post-menopaus*[Title/Abstract] OR retired[Title/Abstract] OR retiree*[Title/Abstract] OR "senior citizen*"[Title/Abstract] OR seniors[Title/Abstract] OR (">=50* years old"[Title/Abstract] OR ">50* years old"[Title/Abstract] OR ">=60* years old"[Title/Abstract] OR ">60* years old"[Title/Abstract] OR ">=70* years old"[Title/Abstract] OR ">7*0 years old"[Title/Abstract] OR ">=80* years old"[Title/Abstract] OR ">80* years old"[Title/Abstract] OR ">=90* years old"[Title/Abstract] OR ">90* years old" [Title/Abstract]) OR ((older[Title/Abstract] AND

adult* [Title/Abstract]) OR "old age*"[Title/Abstract] OR (older[Title/Abstract] AND client* [Title/Abstract]) OR (older[Title/Abstract] AND communit* [Title/Abstract]) OR (older[Title/Abstract] AND female* [Title/Abstract]) OR (older[Title/Abstract] AND individual* [Title/Abstract]) OR (older[Title/Abstract] AND inpatient* [Title/Abstract]) OR (older[Title/Abstract] AND in-patient* [Title/Abstract]) OR (older[Title/Abstract] AND male* [Title/Abstract]) OR (older[Title/Abstract] AND men [Title/Abstract]) OR (older[Title/Abstract] AND outpatient* [Title/Abstract]) OR (older[Title/Abstract] AND out-patient* [Title/Abstract]) OR (older[Title/Abstract] AND patient* [Title/Abstract]) OR (older[Title/Abstract] AND people [Title/Abstract]) OR (older[Title/Abstract] AND person* [Title/Abstract]) OR (older[Title/Abstract] AND population* [Title/Abstract]) OR (older[Title/Abstract] AND women [Title/Abstract])) OR "age in place"[Title/Abstract] OR "aging in place"[Title/Abstract] OR "ageing in place"[Title/Abstract] OR ((active[Title/Abstract] OR healthy [Title/Abstract]) AND (ageing[Title/Abstract] OR aging [Title/Abstract])) OR (friendl*[Title/Abstract] AND (age[Title/Abstract] OR ages[Title/Abstract] OR aged[Title/Abstract] OR aging[Title/Abstract] OR ageing[Title/Abstract] OR elder* [Title/Abstract])))) AND ((systematic*[Title/Abstract] AND review* [Title/Abstract]) OR meta-analy*[Title/Abstract] OR "meta analy*"[Title/Abstract] OR "evidence map"[Title/Abstract] OR "evidence maps"[Title/Abstract] OR "evidence mapping"[Title/Abstract] OR "evidence and gap map"[Title/Abstract] OR EGM[Title/Abstract] OR "evaluation map"[Title/Abstract] OR "evaluation maps"[Title/Abstract] OR "evaluation mapping"[Title/Abstract] OR "systematic map"[Title/Abstract] OR "systematic

maps"[Title/Abstract] OR "systematic mapping"[Title/Abstract] OR "descriptive map"[Title/Abstract] OR "descriptive maps"[Title/Abstract] OR "descriptive mapping"[Title/Abstract])) AND ((((urban[Title/Abstract] OR rural [Title/Abstract]) AND (plan*[Title/Abstract] OR develop*[Title/Abstract] OR design* [Title/Abstract])) OR ((built[Title/Abstract] OR green[Title/Abstract] OR neighb*[Title/Abstract] OR street*[Title/Abstract] OR rural[Title/Abstract] OR urban[Title/Abstract] OR cycling[Title/Abstract] OR driving[Title/Abstract] OR walking [Title/Abstract]) AND environment* [Title/Abstract]) OR ((neighb*[Title/Abstract] OR rural[Title/Abstract] OR urban [Title/Abstract]) AND space* [Title/Abstract]) OR amenit*[Title/Abstract] OR "communit* environment*"[Title/Abstract] OR greener*[Title/Abstract] OR greenness[Title/Abstract] OR greenspace*[Title/Abstract] OR greenway*[Title/Abstract] OR ((green[Title/Abstract] OR blue [Title/Abstract]) AND (area*[Title/Abstract] OR space* [Title/Abstract])) OR "land use*"[Title/Abstract] OR streetscape*[Title/Abstract] OR (street*[Title/Abstract] AND connect* [Title/Abstract]) OR "rural renewal"[Title/Abstract] OR "urban renewal"[Title/Abstract] OR "rural form*"[Title/Abstract] OR "urban form*"[Title/Abstract] OR walkabilit*[Title/Abstract] OR ((population[Title/Abstract] OR residential[Title/Abstract] OR retail[Title/Abstract] OR rural[Title/Abstract] OR urban [Title/Abstract]) AND densit* [Title/Abstract]) OR ((aesthetic*[Title/Abstract] OR garden[Title/Abstract] OR gardens[Title/Abstract] OR park[Title/Abstract] OR parks[Title/Abstract] OR ((plan*[Title/Abstract] OR develop*[Title/Abstract]

OR design* [Title/Abstract]) AND (facility[Title/Abstract] OR facilities [Title/Abstract])) OR ((outdoor*[Title/Abstract] OR public [Title/Abstract]) AND (space*[Title/Abstract] OR building*[Title/Abstract] OR facility[Title/Abstract] OR facilities* [Title/Abstract])) OR ((indoor*[Title/Abstract] OR living[Title/Abstract] OR natural[Title/Abstract] OR objective[Title/Abstract] OR outdoor*[Title/Abstract] OR perceived[Title/Abstract] OR physical[Title/Abstract] OR urban[Title/Abstract] OR rural [Title/Abstract]) AND environment* [Title/Abstract])) AND (home*[Title/Abstract] OR hous*[Title/Abstract] OR residen*[Title/Abstract] OR accommodation*[Title/Abstract] OR city[Title/Abstract] OR cities[Title/Abstract] OR communit*[Title/Abstract] OR neighb*[Title/Abstract] OR rural[Title/Abstract] OR societ*[Title/Abstract] OR town*[Title/Abstract] OR urban[Title/Abstract] OR village* [Title/Abstract])) OR ((neighb*[Title/Abstract] AND (space*[Title/Abstract] OR building*[Title/Abstract] OR facility[Title/Abstract] OR facilities [Title/Abstract])) AND (home*[Title/Abstract] OR hous*[Title/Abstract] OR residen*[Title/Abstract] OR accommodation*[Title/Abstract] OR city[Title/Abstract] OR cities[Title/Abstract] OR communit*[Title/Abstract] OR rural[Title/Abstract] OR societ*[Title/Abstract] OR town*[Title/Abstract] OR urban[Title/Abstract] OR village* [Title/Abstract])) OR ((communit*[Title/Abstract] AND (space*[Title/Abstract] OR building*[Title/Abstract] OR facility[Title/Abstract] OR facilities [Title/Abstract])) AND (home*[Title/Abstract] OR hous*[Title/Abstract] OR residen*[Title/Abstract] OR city[Title/Abstract] OR cities[Title/Abstract] OR neighb*[Title/Abstract] OR rural[Title/Abstract] OR societ*[Title/Abstract] OR town*[Title/Abstract] OR urban[Title/Abstract] OR

village* [Title/Abstract])) OR (infrastructure*[Title/Abstract] AND (city[Title/Abstract] OR cities[Title/Abstract] OR communit*[Title/Abstract] OR cycling[Title/Abstract] OR driving[Title/Abstract] OR neighb*[Title/Abstract] OR pedestrian[Title/Abstract] OR public[Title/Abstract] OR rural[Title/Abstract] OR town*[Title/Abstract] OR transport*[Title/Abstract] OR urban[Title/Abstract] OR village* [Title/Abstract]))) OR (((emission*[Title/Abstract] OR air[Title/Abstract] OR "particulate matter"[Title/Abstract] OR "ambient particulate"[Title/Abstract] OR "ultrafine particulate*"[Title/Abstract] OR "ultrafine particle*"[Title/Abstract] OR UFP [Title/Abstract]) AND (control*[Title/Abstract] OR regulation*[Title/Abstract] OR policy[Title/Abstract] OR policies[Title/Abstract] OR guideline[Title/Abstract] OR intervention[Title/Abstract] OR act[Title/Abstract] OR directive*[Title/Abstract] OR vehicle[Title/Abstract] OR transport*[Title/Abstract] OR traffic[Title/Abstract] OR automobile*[Title/Abstract] OR car[Title/Abstract] OR cars[Title/Abstract] OR industr*[Title/Abstract] OR fuel[Title/Abstract] OR "emission filter*"[Title/Abstract] OR cooking[Title/Abstract] OR heating[Title/Abstract] OR cookstove*[Title/Abstract] OR stove*[Title/Abstract] OR "power generat*"[Title/Abstract] OR zone*[Title/Abstract] OR Olympic[Title/Abstract] OR residential[Title/Abstract] OR "wood burning"[Title/Abstract] OR mobile[Title/Abstract] OR Low[Title/Abstract] OR Lower[Title/Abstract] OR Lowered[Title/Abstract] OR reduc*[Title/Abstract] OR improv*[Title/Abstract] OR clean*[Title/Abstract] OR congestion*[Title/Abstract] OR "coal burning"[Title/Abstract] OR ban[Title/Abstract] OR

bans [Title/Abstract])) OR ((improved[Title/Abstract] OR clean*[Title/Abstract] OR "low emission"[Title/Abstract] OR efficient* [Title/Abstract]) AND (cookstove*[Title/Abstract] OR stove[Title/Abstract] OR stoves[Title/Abstract] OR heater [Title/Abstract]))) OR ((air[Title/Abstract] AND (pollut*[Title/Abstract] OR quality[Title/Abstract] OR ambient [Title/Abstract])) OR (atmospher*[Title/Abstract] AND pollut* [Title/Abstract]) OR ("particulate matter"[Title/Abstract] OR "ambient particulate"[Title/Abstract] OR "ultrafine particulate*"[Title/Abstract] OR "ultrafine particle*"[Title/Abstract] OR UFP [Title/Abstract]) OR ("coarse particle*"[Title/Abstract] OR "black smoke"[Title/Abstract] OR "black carbon"[Title/Abstract] OR "elemental carbon"[Title/Abstract] OR "wood smoke" [Title/Abstract])))

Web of Science Core Collection (via Web of Science)
Fields searched: Abstract

Category		Search terms
Age terms	1	AB=((ageing OR aging OR elder* OR eldest OR geriatric* OR gerontolog* OR age-friendly OR "healthy ageing" OR "active ageing" OR ageing OR aging OR "care home resident*" OR community-dwelling OR elder* OR frail* OR geriatric* OR "late life" OR "later life" OR "mature adult*" OR nonagenarian* OR "nursing home resident*" OR octogenarian* OR "old age" OR "oldest old" OR pensioner* OR postmenopaus* OR post-menopaus* OR retired OR retiree* OR "senior citizen*" OR seniors OR (">=50? years old" OR ">50? years old" OR ">=60? years old" OR ">60? years old" OR ">=70? years old" OR ">7?0 years old" OR ">=80? years old" OR ">80? years old" OR ">=90? years old" OR ">90? years old") OR ((older NEAR/3 adult*) OR "old age*" OR (older NEAR/3 client*) OR (older NEAR/3 communit*) OR (older NEAR/3 female*) OR (older NEAR/3 individual*) OR (older NEAR/3 inpatient*) OR (older NEAR/3 in-patient*) OR (older NEAR/3

		male*) OR (older NEAR/3 men) OR (older NEAR/3 outpatient*) OR (older NEAR/3 out-patient*) OR (older NEAR/3 patient*) OR (older NEAR/3 people) OR (older NEAR/3 person*) OR (older NEAR/3 population*) OR (older NEAR/3 women)) OR "age in place" OR "aging in place" OR "ageing in place" OR ((active OR healthy) NEAR/3 (ageing OR aging)) OR (friendl* NEAR/3 (age OR ages OR aged OR aging OR ageing OR elder*))))
Methodology term (s)	2	AB=((systematic* NEAR/2 review*) OR meta-analy* OR "meta analy*" OR "evidence map" OR "evidence maps" OR "evidence mapping" OR "evidence and gap map" OR EGM OR "evaluation map" OR "evaluation maps" OR "evaluation mapping" OR "systematic map" OR "systematic maps" OR "systematic mapping" OR "descriptive map" OR "descriptive maps" OR "descriptive mapping")
Intervention terms	3<br><br>Domain 1: Transportation	AB=(("active* transport*" OR "active* travel*" OR ((bicycl* OR cycl* OR driv* OR motorcycl* OR walk*) NEAR/3 (coach* OR course* OR educat* OR instruct* OR lesson* OR school* OR

train*)) OR ((bicycl* OR cycli* OR driv* OR motorcycl* OR motorist* OR passenger* OR pedestrian* OR rider* OR walk* OR traffic* OR transport* OR automobile* OR bus OR buses OR car OR cars OR carpark* OR "dial a ride" OR garage* OR metro OR railway* OR parking* OR sidewalk* OR subway* OR taxi* OR train OR trains OR underground OR vehicle* OR crossing* OR highway* OR lane OR lanes OR road OR roads OR street*) NEAR/3 (abilit* OR access* OR afford* OR behaviour* OR behavior* OR capacit* OR danger* OR difficult* OR ergonomic* OR habit* OR safe* OR stable OR stability OR unsafe OR ((fare* OR price* OR ticket*) NEAR/3 (discount* OR free OR reduc*)) OR accident* OR collision* OR crash* OR hazard* OR incident* OR speed*)) OR ((bicycl* OR cycli* OR driv* OR motorcycl* OR automobile* OR bus OR buses OR car OR cars OR metro OR railway* OR subway* OR taxi* OR train OR trains OR underground OR vehicle*) NEAR/3 (adapt* OR alteration* OR modif* OR support OR usability))))

	4	#1 AND #2 AND #3
Age terms	1	AB=((ageing OR aging OR elder* OR eldest OR geriatric* OR gerontolog* OR age-friendly OR "healthy ageing" OR "active ageing" OR ageing OR aging OR "care home resident*" OR community-dwelling OR elder* OR frail* OR geriatric* OR "late life" OR "later life" OR "mature adult*" OR nonagenarian* OR "nursing home resident*" OR octogenarian* OR "old age" OR "oldest old" OR pensioner* OR postmenopaus* OR post-menopaus* OR retired OR retiree* OR "senior citizen*" OR seniors OR (">=50? years old" OR ">50? years old" OR ">=60? years old" OR ">60? years old" OR ">=70? years old" OR ">7?0 years old" OR ">=80? years old" OR ">80? years old" OR ">=90? years old" OR ">90? years old") OR ((older NEAR/3 adult*) OR "old age*" OR (older NEAR/3 client*) OR (older NEAR/3 communit*) OR (older NEAR/3 female*) OR (older NEAR/3 individual*) OR (older NEAR/3 inpatient*) OR (older NEAR/3 in-patient*) OR (older NEAR/3 male*) OR (older NEAR/3 men) OR (older NEAR/3 outpatient*) OR (older

		NEAR/3 out-patient*) OR (older NEAR/3 patient*) OR (older NEAR/3 people) OR (older NEAR/3 person*) OR (older NEAR/3 population*) OR (older NEAR/3 women)) OR "age in place" OR "aging in place" OR "ageing in place" OR ((active OR healthy) NEAR/3 (ageing OR aging)) OR (friendl* NEAR/3 (age OR ages OR aged OR aging OR ageing OR elder*))))
Methodology term (s)	2	AB=((systematic* NEAR/2 review*) OR meta-analy* OR "meta analy*" OR "evidence map" OR "evidence maps" OR "evidence mapping" OR "evidence and gap map" OR EGM OR "evaluation map" OR "evaluation maps" OR "evaluation mapping" OR "systematic map" OR "systematic maps" OR "systematic mapping" OR "descriptive map" OR "descriptive maps" OR "descriptive mapping")
Intervention terms	3<br><br>Domain 2: Housing	AB=(((ambient* NEAR/3 assist* NEAR/3 living) OR ((smart OR automat*) NEAR/3 home*) OR (smart NEAR/3 environment*) OR ((home* OR hous* OR residen* OR accommodation* OR bath* OR bedroom* OR kitchen* OR room* OR shower* OR

		stair* OR toilet*) NEAR/3 (access* OR adapt* OR alteration* OR modif* OR renovat* OR support OR usability)) OR ((elevator* OR handrail* OR "hand rail*" OR lift OR lifts OR ramp OR ramps OR gerontechnolog* OR "voice assistant*" OR (service NEAR/3 robot*) OR ((assist* OR intelligen* OR safe* OR self-help* OR welfare*) NEAR/3 (aid OR aids OR device* OR platform* OR robot* OR solution* OR technolog*))) AND (home* OR hous* OR residen* OR accommodation* OR city OR cities OR communit* OR environment* OR neighb* OR rural OR societ* OR town* OR urban OR village*))))
4		AB=("naturally occurring retirement communi*" or NORC or housing or "cohousi*" or "co-hous*" or "university based retirement communi*" or "UBRC*" or apartment* or bungalow* or dwelling* or housing or landlord* or rehous* or squatter* or tenant* or bedsit or "bedsitting" or "bed sit" or "bed sitting" or "residential care" or highrise* or "high rise*" or homeowner* or "home owner*" or "indoor air

		qualit*” or “living environment*” or “live environment*”or “built environment” or “living quarter*” or multistor* or “multi stor*” or owner* occup* or towerblock* or “tower block*” or “cottage*" or "flat*" or "houses" or home or stair or staircase or stairwell or maisonette* or indoor*)
	5	#3 OR #4
	6	#1 AND #2 AND #5
Age terms	1	AB=("social participat*" OR "community involve*" OR community-dwelling OR "civic engage*" OR "active citizen*" OR "social involve*" OR "community participat*" OR "public engag*" OR "societal engage*" OR "participatory citizenship" OR "active participat*" OR "collective action" OR "engage* in society" OR "group participat*" OR "collaborat* involve*" OR "social integrat*" OR "involve* in public life" OR "social connect*" OR "social health" OR "social capital")
Methodology term (s)	2	AB=((systematic* NEAR/2 review*) OR meta-analy* OR "meta analy*" OR "evidence map" OR "evidence maps" OR "evidence mapping" OR "evidence and gap map" OR

		EGM OR "evaluation map" OR "evaluation maps" OR "evaluation mapping" OR "systematic map" OR "systematic maps" OR "systematic mapping" OR "descriptive map" OR "descriptive maps" OR "descriptive mapping")
Intervention terms	3<br><br>Domain 3: Social participation	AB=("social participat*" OR "community involve*" OR community-dwelling OR "civic engage*" OR "active citizen*" OR "social involve*" OR "community participat*" OR "public engag*" OR "societal engage*" OR "participatory citizenship" OR "active participat*" OR "collective action" OR "engage* in society" OR "group participat*" OR "collaborat* involve*" OR "social integrat*" OR "involve* in public life" OR "social connect*" OR "social health" OR "social capital")
	4	#3 AND #2 AND #1
Age terms	1	AB=((ageing OR aging OR elder* OR eldest OR geriatric* OR gerontolog* OR age-friendly OR "healthy ageing" OR "active ageing" OR ageing OR aging OR "care home resident*" OR community-dwelling OR elder* OR frail* OR geriatric* OR "late life" OR "later life" OR "mature

adult*" OR nonagenarian* OR "nursing home resident*" OR octogenarian* OR "old age" OR "oldest old" OR pensioner* OR postmenopaus* OR post-menopaus* OR retired OR retiree* OR "senior citizen*" OR seniors OR (">=50? years old" OR ">50? years old" OR ">=60? years old" OR ">60? years old" OR ">=70? years old" OR ">7?0 years old" OR ">=80? years old" OR ">80? years old" OR ">=90? years old" OR ">90? years old") OR ((older NEAR/3 adult*) OR "old age*" OR (older NEAR/3 client*) OR (older NEAR/3 communit*) OR (older NEAR/3 female*) OR (older NEAR/3 individual*) OR (older NEAR/3 inpatient*) OR (older NEAR/3 in-patient*) OR (older NEAR/3 male*) OR (older NEAR/3 men) OR (older NEAR/3 outpatient*) OR (older NEAR/3 out-patient*) OR (older NEAR/3 patient*) OR (older NEAR/3 people) OR (older NEAR/3 person*) OR (older NEAR/3 population*) OR (older NEAR/3 women)) OR "age in place" OR "aging in place" OR "ageing in place" OR ((active OR healthy) NEAR/3 (ageing OR aging)) OR (friendl* NEAR/3 (age OR ages OR

		aged OR aging OR ageing OR elder*))))
Methodology term (s)	2	AB=((systematic* NEAR/2 review*) OR meta-analy* OR "meta analy*" OR "evidence map" OR "evidence maps" OR "evidence mapping" OR "evidence and gap map" OR EGM OR "evaluation map" OR "evaluation maps" OR "evaluation mapping" OR "systematic map" OR "systematic maps" OR "systematic mapping" OR "descriptive map" OR "descriptive maps" OR "descriptive mapping")
Intervention terms	3<br><br>Domain 4: Respect and social inclusion	AB=("social inclusion" OR "community inclusion" OR e-inclusion OR "digital inclusion" OR "social cohesion" OR "community cohesion" OR "neighbo* cohesion" OR "social involvement" OR "community involvement" OR "social integration" OR "community integration" OR "social engagement" OR "civic engagement" OR "community engagement" OR intergeneration* OR "social recognition" OR "information and communication technolog*" OR "social exclusion" OR "neighbo* inclusion" OR "neighbo* exclusion" OR "community participation"

		OR "social participation" OR ageism OR agism OR "age stereotyp*" OR "ageing stereotyp*" OR "aging stereotyp*" OR "age discrimination" OR "ageing discrimination" OR "aging discrimination" OR "digital divide" OR "social interaction*" OR "social responsabilit*" OR "social capital" OR "social networks" OR "access services" OR "access information" OR "access opportunit*" OR "access faciliti*" OR "access volunteer*" OR "access learning" OR "social exchange*" OR solidarity)
	4	#3 AND #2 AND #1
Age terms	1	AB=((ageing OR aging OR elder* OR eldest OR geriatric* OR gerontolog* OR age-friendly OR "healthy ageing" OR "active ageing" OR ageing OR aging OR "care home resident*" OR community-dwelling OR elder* OR frail* OR geriatric* OR "late life" OR "later life" OR "mature adult*" OR nonagenarian* OR "nursing home resident*" OR octogenarian* OR "old age" OR "oldest old" OR pensioner* OR postmenopaus* OR post-menopaus* OR retired OR retiree* OR "senior citizen*" OR seniors OR (">=50? years

		old" OR ">50? years old" OR ">=60? years old" OR ">60? years old" OR ">=70? years old" OR ">7?0 years old" OR ">=80? years old" OR ">80? years old" OR ">=90? years old" OR ">90? years old") OR ((older NEAR/3 adult*) OR "old age*" OR (older NEAR/3 client*) OR (older NEAR/3 communit*) OR (older NEAR/3 female*) OR (older NEAR/3 individual*) OR (older NEAR/3 inpatient*) OR (older NEAR/3 in-patient*) OR (older NEAR/3 male*) OR (older NEAR/3 men) OR (older NEAR/3 outpatient*) OR (older NEAR/3 out-patient*) OR (older NEAR/3 patient*) OR (older NEAR/3 people) OR (older NEAR/3 person*) OR (older NEAR/3 population*) OR (older NEAR/3 women)) OR "age in place" OR "aging in place" OR "ageing in place" OR ((active OR healthy) NEAR/3 (ageing OR aging)) OR (friendl* NEAR/3 (age OR ages OR aged OR aging OR ageing OR elder*))))
Methodology term (s)	2	AB=((systematic* NEAR/2 review*) OR meta-analy* OR "meta analy*" OR "evidence map" OR "evidence maps" OR "evidence mapping" OR "evidence and gap map" OR

		EGM OR "evaluation map" OR "evaluation maps" OR "evaluation mapping" OR "systematic map" OR "systematic maps" OR "systematic mapping" OR "descriptive map" OR "descriptive maps" OR "descriptive mapping")
Intervention terms	3<br><br>Domain 5: Civic participation and employment	AB=("civil participation" OR "citizen participation" OR "community engagement" OR "public participation" OR "civic engagement" OR "political participation" OR employ* OR job OR occupation OR work* OR career OR "labour market" OR "labor market" OR "job market" OR workforce OR independence)
	4	#3 AND #2 AND #1
Age terms	1	AB=((ageing OR aging OR elder* OR eldest OR geriatric* OR gerontolog* OR age-friendly OR "healthy ageing" OR "active ageing" OR ageing OR aging OR "care home resident*" OR community-dwelling OR elder* OR frail* OR geriatric* OR "late life" OR "later life" OR "mature adult*" OR nonagenarian* OR "nursing home resident*" OR octogenarian* OR "old age" OR "oldest old" OR pensioner* OR postmenopaus* OR post-menopaus* OR retired OR

		retiree* OR "senior citizen*" OR seniors OR (">=50? years old" OR ">50? years old" OR ">=60? years old" OR ">60? years old" OR ">=70? years old" OR ">7?0 years old" OR ">=80? years old" OR ">80? years old" OR ">=90? years old" OR ">90? years old") OR ((older NEAR/3 adult*) OR "old age*" OR (older NEAR/3 client*) OR (older NEAR/3 communit*) OR (older NEAR/3 female*) OR (older NEAR/3 individual*) OR (older NEAR/3 inpatient*) OR (older NEAR/3 in-patient*) OR (older NEAR/3 male*) OR (older NEAR/3 men) OR (older NEAR/3 outpatient*) OR (older NEAR/3 out-patient*) OR (older NEAR/3 patient*) OR (older NEAR/3 people) OR (older NEAR/3 person*) OR (older NEAR/3 population*) OR (older NEAR/3 women)) OR "age in place" OR "aging in place" OR "ageing in place" OR ((active OR healthy) NEAR/3 (ageing OR aging)) OR (friendl* NEAR/3 (age OR ages OR aged OR aging OR ageing OR elder*))))
Methodology term (s)	2	AB=((systematic* NEAR/2 review*) OR meta-analy* OR "meta analy*" OR "evidence map" OR "evidence maps" OR

		"evidence mapping" OR "evidence and gap map" OR EGM OR "evaluation map" OR "evaluation maps" OR "evaluation mapping" OR "systematic map" OR "systematic maps" OR "systematic mapping" OR "descriptive map" OR "descriptive maps" OR "descriptive mapping")
Intervention terms	3<br><br>Domain 6: Communication and information	AB=(communication OR information OR negot* OR counselling OR learning OR literacy OR training OR coaching)
	4	#3 AND #2 AND #1
Age terms	1	AB=((ageing OR aging OR elder* OR eldest OR geriatric* OR gerontolog* OR age-friendly OR "healthy ageing" OR "active ageing" OR ageing OR aging OR "care home resident*" OR community-dwelling OR elder* OR frail* OR geriatric* OR "late life" OR "later life" OR "mature adult*" OR nonagenarian* OR "nursing home resident*" OR octogenarian* OR "old age" OR "oldest old" OR pensioner* OR postmenopaus* OR post-menopaus* OR retired OR retiree* OR "senior citizen*" OR seniors OR (">=50? years old" OR ">50? years old" OR ">=60? years old" OR ">60? years old" OR ">=70? years

		old" OR ">7?0 years old" OR ">=80? years old" OR ">80? years old" OR ">=90? years old" OR ">90? years old") OR ((older NEAR/3 adult*) OR "old age*" OR (older NEAR/3 client*) OR (older NEAR/3 communit*) OR (older NEAR/3 female*) OR (older NEAR/3 individual*) OR (older NEAR/3 inpatient*) OR (older NEAR/3 in-patient*) OR (older NEAR/3 male*) OR (older NEAR/3 men) OR (older NEAR/3 outpatient*) OR (older NEAR/3 out-patient*) OR (older NEAR/3 patient*) OR (older NEAR/3 people) OR (older NEAR/3 person*) OR (older NEAR/3 population*) OR (older NEAR/3 women)) OR "age in place" OR "aging in place" OR "ageing in place" OR ((active OR healthy) NEAR/3 (ageing OR aging)) OR (friendl* NEAR/3 (age OR ages OR aged OR aging OR ageing OR elder*))))
Methodology term (s)	2	AB=((systematic* NEAR/2 review*) OR meta-analy* OR "meta analy*" OR "evidence map" OR "evidence maps" OR "evidence mapping" OR "evidence and gap map" OR EGM OR "evaluation map" OR "evaluation maps" OR "evaluation mapping" OR

		"systematic map" OR "systematic maps" OR "systematic mapping" OR "descriptive map" OR "descriptive maps" OR "descriptive mapping")
Intervention terms	3<br><br>Domain 7: Community support and health services	AB=("community support" OR "community assist*" OR "community aid" OR "local support" OR "social support" OR "community resource*" OR "community service*" OR "mutual aid" OR "community welfare" OR "community development" OR "grassroot* support" OR "volunteer support" OR "community engagement*" OR "community involvement*" OR "health service*" OR "healthcare service*" OR "medical service*" OR "healthcare facility*" OR "healthcare system*" OR "healthcare delivery" OR "healthcare access" OR "healthcare utilisation" OR "healthcare utilization" OR "healthcare management" OR "healthcare policy" OR "healthcare administration" OR "healthcare quality" OR "healthcare reform" OR "assisted living" OR AL OR "retirement home*" OR "retirement communit*" OR "supportive hous*" OR "long-term care" OR "senior care" OR "aged care")

	4	#3 AND #2 AND #1
Age terms	1	AB=((ageing OR aging OR elder* OR eldest OR geriatric* OR gerontolog* OR age-friendly OR "healthy ageing" OR "active ageing" OR ageing OR aging OR "care home resident*" OR community-dwelling OR elder* OR frail* OR geriatric* OR "late life" OR "later life" OR "mature adult*" OR nonagenarian* OR "nursing home resident*" OR octogenarian* OR "old age" OR "oldest old" OR pensioner* OR postmenopaus* OR post-menopaus* OR retired OR retiree* OR "senior citizen*" OR seniors OR (">=50? years old" OR ">50? years old" OR ">=60? years old" OR ">60? years old" OR ">=70? years old" OR ">7?0 years old" OR ">=80? years old" OR ">80? years old" OR ">=90? years old" OR ">90? years old") OR ((older NEAR/3 adult*) OR "old age*" OR (older NEAR/3 client*) OR (older NEAR/3 communit*) OR (older NEAR/3 female*) OR (older NEAR/3 individual*) OR (older NEAR/3 inpatient*) OR (older NEAR/3 in-patient*) OR (older NEAR/3 male*) OR (older NEAR/3 men) OR (older NEAR/3 outpatient*) OR (older

		NEAR/3 out-patient*) OR (older NEAR/3 patient*) OR (older NEAR/3 people) OR (older NEAR/3 person*) OR (older NEAR/3 population*) OR (older NEAR/3 women)) OR "age in place" OR "aging in place" OR "ageing in place" OR ((active OR healthy) NEAR/3 (ageing OR aging)) OR (friendl* NEAR/3 (age OR ages OR aged OR aging OR ageing OR elder*))))
Methodology terms	2	AB=((systematic* NEAR/2 review*) OR meta-analy* OR "meta analy*" OR "evidence map" OR "evidence maps" OR "evidence mapping" OR "evidence and gap map" OR EGM OR "evaluation map" OR "evaluation maps" OR "evaluation mapping" OR "systematic map" OR "systematic maps" OR "systematic mapping" OR "descriptive map" OR "descriptive maps" OR "descriptive mapping")
Intervention terms	3<br><br>Domain 8: Outdoor space and housing	AB=((((urban OR rural) NEAR/3 (plan* OR develop* OR design*)) OR ((built OR green OR neighb* OR street* OR rural OR urban OR cycling OR driving OR walking) NEAR/3 environment*) OR ((neighb* OR rural OR urban) NEAR/3 space*) OR amenit* OR

"communit* environment*" OR greener* OR greenness OR greenspace* OR greenway* OR ((green OR blue) NEAR/3 (area* OR space*)) OR "land use*" OR streetscape* OR (street* NEAR/3 connect*) OR "rural renewal" OR "urban renewal" OR "rural form*" OR "urban form*" OR walkabilit* OR ((population OR residential OR retail OR rural OR urban) NEAR/3 densit*) OR ((aesthetic* OR garden OR gardens OR park OR parks OR ((plan* OR develop* OR design*) NEAR/3 (facility OR facilities)) OR ((outdoor* OR public) NEAR/3 (space* OR building* OR facility OR facilities*)) OR ((indoor* OR living OR natural OR objective OR outdoor* OR perceived OR physical OR urban OR rural) NEAR/3 environment*)) AND (home* OR hous* OR residen* OR accommodation* OR city OR cities OR communit* OR neighb* OR rural OR societ* OR town* OR urban OR village*)) OR ((neighb* NEAR/3 (space* OR building* OR facility OR facilities)) AND (home* OR hous* OR residen* OR accommodation* OR city OR

cities OR communit* OR rural OR societ* OR town* OR urban OR village*)) OR ((communit* NEAR/3 (space* OR building* OR facility OR facilities)) AND (home* OR hous* OR residen* OR city OR cities OR neighb* OR rural OR societ* OR town* OR urban OR village*)) OR (infrastructure* AND (city OR cities OR communit* OR cycling OR driving OR neighb* OR pedestrian OR public OR rural OR town* OR transport* OR urban OR village*))) OR (((emission* OR air OR "particulate matter" OR "ambient particulate" OR "ultrafine particulate*" OR "ultrafine particle*" OR UFP) NEAR/4 (control* OR regulation* OR policy OR policies OR guideline OR intervention OR act OR directive* OR vehicle OR transport* OR traffic OR automobile* OR car OR cars OR industr* OR fuel OR "emission filter*" OR cooking OR heating OR cookstove* OR stove* OR "power generat*" OR zone* OR Olympic OR residential OR "wood burning" OR mobile OR Low OR Lower OR Lowered OR reduc* OR improv* OR clean* OR congestion* OR "coal

	burning" OR ban OR bans)) OR ((improved OR clean* OR "low emission" OR efficient*) NEAR/1 (cookstove* OR stove OR stoves OR heater))) OR ((air NEAR/2 (pollut* OR quality OR ambient)) OR (atmospher* NEAR/2 pollut*) OR ("particulate matter" OR "ambient particulate" OR "ultrafine particulate*" OR "ultrafine particle*" OR UFP) OR ("coarse particle*" OR "black smoke" OR "black carbon" OR "elemental carbon" OR "wood smoke")))
4	#3 AND #2 AND #1

CINAHL, ERIC, PsycINFO, ASU, BSU, SWA, CMMC, PSC, EconLit, AWI, GreenFILE (Via EBSCO)

Fields searched: Abstract

Category			Search terms
A) Age terms	1	AT1	(ageing OR aging OR elder* OR eldest OR geriatric* OR gerontolog* OR age-friendly OR "healthy ageing" OR "active ageing" OR ageing OR aging OR "care home resident*" OR community-dwelling OR elder* OR frail* OR geriatric* OR "late life" OR "later life" OR "mature adult*" OR nonagenarian* OR "nursing home resident*" OR octogenarian* OR "old age" OR "oldest old" OR pensioner* OR postmenopaus* OR post-menopaus* OR retired OR retiree* OR "senior citizen*" OR seniors OR (">=50? years old" OR ">50? years old" OR ">=60? years old" OR ">60? years old" OR ">=70? years old" OR ">7?0 years old" OR ">=80? years old" OR ">80? years old" OR ">=90? years old" OR ">90? years old") OR ((older N3 adult*) OR "old age*" OR (older N3 client*) OR (older N3 communit*) OR (older N3 female*) OR (older N3 individual*) OR (older N3 inpatient*) OR (older N3 in-patient*) OR (older N3

			male*) OR (older N3 men) OR (older N3 outpatient*) OR (older N3 out-patient*) OR (older N3 patient*) OR (older N3 people) OR (older N3 person*) OR (older N3 population*) OR (older N3 women)) OR "age in place" OR "aging in place" OR "ageing in place" OR ((active OR healthy) N3 (ageing OR aging)) OR (friendl* N3 (age OR ages OR aged OR aging OR ageing OR elder*)))
B) Methodology terms		MT1	AB ((systematic* N2 review*) OR meta-analy* OR "meta analy*" OR "evidence map" OR "evidence maps" OR "evidence mapping" OR "evidence and gap map" OR EGM OR "evaluation map" OR "evaluation maps" OR "evaluation mapping" OR "systematic map" OR "systematic maps" OR "systematic mapping" OR "descriptive map" OR "descriptive maps" OR "descriptive mapping")
c) Interventions terms	Domain 1: Transportation	DT1	("active* transport*" OR "active* travel*" OR ((bicycl* OR cycl* OR driv* OR motorcycl* OR walk*) N3 (coach* OR course* OR educat* OR instruct* OR lesson* OR school* OR train*)) OR ((bicycl* OR cycli* OR driv* OR motorcycl* OR motorist* OR passenger* OR pedestrian* OR rider* OR walk* OR traffic* OR transport* OR automobile* OR bus OR buses OR car OR cars OR carpark* OR "dial a ride" OR garage* OR metro OR railway* OR parking* OR sidewalk* OR subway* OR taxi* OR train OR trains OR underground OR vehicle* OR crossing* OR highway* OR lane OR lanes OR road OR roads OR street*) N3 (abilit* OR access* OR afford* OR behaviour* OR behavior* OR capacit* OR danger* OR difficult* OR ergonomic* OR habit* OR safe* OR stable OR stability OR unsafe OR ((fare* OR price* OR ticket*) N3 (discount* OR free OR reduc*)) OR accident* OR collision* OR crash* OR hazard* OR incident* OR speed*)) OR ((bicycl* OR cycli* OR driv* OR motorcycl* OR automobile* OR bus OR buses OR car OR cars OR metro OR railway* OR subway* OR taxi* OR train OR trains OR underground OR vehicle*) N3 (adapt* OR alteration* OR modif* OR support OR usability)))
		DT2	AT1 AND MT1 AND DT1
	Domain 2: Housing	DH1	((ambient* N3 assist* N3 living) OR ((smart OR automat*) N3 home*) OR (smart N3 environment*) OR

		((home* OR hous* OR residen* OR accommodation* OR bath* OR bedroom* OR kitchen* OR room* OR shower* OR stair* OR toilet*) N3 (access* OR adapt* OR alteration* OR modif* OR renovat* OR support OR usability)) OR ((elevator* OR handrail* OR "hand rail*" OR lift OR lifts OR ramp OR ramps OR gerontechnolog* OR "voice assistant*" OR (service N3 robot*) OR ((assist* OR intelligen* OR safe* OR self-help* OR welfare*) N3 (aid OR aids OR device* OR platform* OR robot* OR solution* OR technolog*))) AND (home* OR hous* OR residen* OR accommodation* OR city OR cities OR communit* OR environment* OR neighb* OR rural OR societ* OR town* OR urban OR village*)))
	DH2	"naturally occurring retirement communi*" OR NORC OR housing OR cohousi* OR co-hous* OR "university based retirement communi*" OR UBRC* OR apartment* OR bungalow* OR dwelling* OR housing OR landlord* OR rehous* OR squatter* OR tenant* OR bedsit OR bedsitting OR "bed sit" OR "bed sitting" OR "residential care" OR highrise* OR "high rise*" OR homeowner* OR "home owner*" OR "indoor air qualit*" OR "living environment*" OR "live environment*" OR "built environment" OR "living quarter*" OR multistor* OR "multi stor*" OR "owner* occup*" OR towerblock* OR "tower block*" OR cottage* OR flat* OR houses OR home OR stair OR staircase OR stairwell OR maisonette* OR indoor*
	DH3	DH1 OR DH2
	DH4	AT1 AND MT1 AND DH3
Domain 3: Social participation	DSP1	AB (“social participat*” or “community involve*” or “community-dwelling” or “civic engage*” or “active citizen*” or “social involve*” or “community participat*” or “public engag*” or “societal engage*” or “participatory citizenship” or “active participat*” or “collective action” or “engage* in society” or “group participat*” or “collaborat* involve*” or “social integrat*” or “involve* in public life” or “social connect*” or “social health” or “social capital”)
	DSP2	AT1 AND MT1 AND DSP1
	DRSI1	"social inclusion" OR "community inclusion" OR e-inclusion OR "digital inclusion" OR "social cohesion" OR

Domain 4: Respect and Social inclusion		"community cohesion" OR "neighbo* cohesion" OR "social involvement" OR "community involvement" OR "social integration" OR "community integration" OR "social engagement" OR "civic engagement" OR "community engagement" OR intergeneration* OR "social recognition" OR "information and communication technolog*" OR "social exclusion" OR "neighbo* inclusion" OR "neighbo* exclusion" OR "community participation" OR "social participation" OR ageism OR agism OR "age stereotyp*" OR "ageing stereotyp*" OR "aging stereotyp*" OR "age discrimination" OR "ageing discrimination" OR "aging discrimination" OR "digital divide" OR "social interaction*" OR "social responsabilit*" OR "social capital" OR "social networks" OR "access services" OR "access information" OR "access opportunit*" OR "access faciliti*" OR "access volunteer*" OR "access learning" OR "social exchange*" OR solidarity
	DRSI2	AT1 AND MT1 AND DRSI1
Domain 5: Civil participation and employment	DCPE1	AB ("civil participation" OR "citizen participation" OR "community engagement" OR "public participation" OR "civic engagement" OR "political participation" OR "employ*" OR "job" OR "occupation" OR "work*" OR "career" OR "labour market" or "labor market" OR "job market" OR "workforce" OR independence)
	DCPE2	AT1 AND MT1 AND DCPE1
Domain 6: Communication and Information	DCI1	AB (communication OR information OR negot* OR counselling OR learning OR literacy OR training OR coaching)
	DCI2	AT1 AND MT1 AND DCI1
Domain 7: Community support and health services	DCSHS1	"community support" OR "community assist*" OR "community aid" OR "local support" OR "social support" OR "community resource*" OR "community service*" OR "mutual aid" OR "community welfare" OR "community development" OR "grassroot* support" OR "volunteer support" OR "community engagement*" OR "community involvement*" OR "health service*" OR "healthcare service*" OR "medical service*" OR "healthcare facility*" OR "healthcare system*" OR "healthcare delivery" OR "healthcare access" OR "healthcare utilisation" OR "healthcare utilization" OR

		"healthcare management" OR "healthcare policy" OR "healthcare administration" OR "healthcare quality" OR "healthcare reform" OR "assisted living" OR AL OR "retirement home*" OR "retirement communit*" OR "supportive hous*" OR "long-term care" OR "senior care" OR "aged care"
	DCSHS1	AT1 AND MT1 AND DCSHS1
Domain 8: Outdoor space and buildings	DOSB1	(((urban OR rural) N3 (plan* OR develop* OR design*)) OR ((built OR green OR neighb* OR street* OR rural OR urban OR cycling OR driving OR walking) N3 environment*) OR ((neighb* OR rural OR urban) N3 space*) OR amenit* OR "communit* environment*" OR greener* OR greenness OR greenspace* OR greenway* OR ((green OR blue) N3 (area* OR space*)) OR "land use*" OR streetscape* OR (street* N3 connect*) OR "rural renewal" OR "urban renewal" OR "rural form*" OR "urban form*" OR walkabilit* OR ((population OR residential OR retail OR rural OR urban) N3 densit*) OR ((aesthetic* OR garden OR gardens OR park OR parks OR ((plan* OR develop* OR design*) N3 (facility OR facilities)) OR ((outdoor* OR public) N3 (space* OR building* OR facility OR facilities*)) OR ((indoor* OR living OR natural OR objective OR outdoor* OR perceived OR physical OR urban OR rural) N3 environment*)) AND (home* OR hous* OR residen* OR accommodation* OR city OR cities OR communit* OR neighb* OR rural OR societ* OR town* OR urban OR village*)) OR ((neighb* N3 (space* OR building* OR facility OR facilities)) AND (home* OR hous* OR residen* OR accommodation* OR city OR cities OR communit* OR rural OR societ* OR town* OR urban OR village*)) OR ((communit* N3 (space* OR building* OR facility OR facilities)) AND (home* OR hous* OR residen* OR city OR cities OR neighb* OR rural OR societ* OR town* OR urban OR village*)) OR (infrastructure* AND (city OR cities OR communit* OR cycling OR driving OR neighb* OR pedestrian OR public OR rural OR town* OR transport* OR urban OR village*))) OR

	(((emission* OR air OR "particulate matter" OR "ambient particulate" OR "ultrafine particulate*" OR "ultrafine particle*" OR UFP) N4 (control* OR regulation* OR policy OR policies OR guideline OR intervention OR act OR directive* OR vehicle OR transport* OR traffic OR automobile* OR car OR cars OR industr* OR fuel OR "emission filter*" OR cooking OR heating OR cookstove* OR stove* OR "power generat*" OR zone* OR Olympic OR residential OR "wood burning" OR mobile OR Low OR Lower OR Lowered OR reduc* OR improv* OR clean* OR congestion* OR "coal burning" OR ban OR bans)) OR ((improved OR clean* OR "low emission" OR efficient*) N1 (cookstove* OR stove OR stoves OR heater))) OR ((air N2 (pollut* OR quality OR ambient)) OR (atmospher* N2 pollut*) OR ("particulate matter" OR "ambient particulate" OR "ultrafine particulate*" OR "ultrafine particle*" OR UFP) OR ("coarse particle*" OR "black smoke" OR "black carbon" OR "elemental carbon" OR "wood smoke"))
DOSB2	AT1 AND MT1 AND DOSB1

Cochrane Database of Systematic Reviews (via Cochrane library)

(ageing OR aging OR elder* OR eldest OR geriatric* OR gerontolog* OR age-friendly OR "healthy ageing" OR "active ageing" OR ageing OR aging OR ("care home" NEXT resident*) OR community-dwelling OR elder* OR frail* OR geriatric* OR "late life" OR "later life" OR ("mature" NEXT adult*) OR nonagenarian* OR ("nursing home" NEXT resident*) OR octogenarian* OR "old age" OR "oldest old" OR pensioner* OR postmenopaus* OR post-menopaus* OR retired OR retiree* OR ("senior" NEXT citizen*) OR seniors OR (">=50? years old" OR ">50? years old" OR ">=60? years old" OR ">60? years old" OR ">=70? years old" OR ">7?0 years old" OR ">=80? years old" OR ">80? years old" OR ">=90? years old" OR ">90? years old") OR ((older NEAR/3 adult*) OR ("old" NEXT age*) OR (older NEAR/3 client*) OR (older NEAR/3 communit*) OR (older NEAR/3 female*) OR (older NEAR/3 individual*) OR (older NEAR/3 inpatient*) OR (older NEAR/3 in-patient*) OR (older NEAR/3 male*) OR (older NEAR/3 men) OR (older NEAR/3 outpatient*) OR (older NEAR/3 out-patient*) OR (older NEAR/3 patient*) OR (older NEAR/3 people) OR (older NEAR/3 person*) OR (older NEAR/3 population*) OR (older NEAR/3 women)) OR "age in place" OR "aging in place" OR

"ageing in place" OR ((active OR healthy) NEAR/3 (ageing OR aging)) OR (friendl* NEAR/3 (age OR ages OR aged OR aging OR ageing OR elder*))) in Title Abstract Keyword - (Word variations have been searched)

Appendix 2: Eligibility criteria

Criteria	Inclusion	Exclusion
**Population**	For the purpose of this EGM, we will accept operational definitions for "older adults" from 50 years old onwards, considering that intervention studies commonly recruit participants at earlier ages to account for the lag between the intervention and its impacts. Whenever possible, estimates should be grouped by sub-population (e.g., age brackets, gender, socioeconomic status, degree of urbanization, and low- and middle-income settings).<br>• Setting: We will consider participants from cities and communities of any size and location in urban, peri-urban and rural areas (as per the degree of urbanization).	- Not older adults (< 50 years old)<br>- Mixed population without disaggregated data for target population (≥ 50 years old)<br>-Hospitalized patients
**Intervention**	1. Interventions under the eight domains of action according to the WHO framework and identify both interventions specifically targeted at improving the lives of older adults (e.g., public transport fare exemption for people aged 60 and over) and interventions with a broader target population that report the impact on older adults' mobility (e.g., interventions for accessible transportation). The eight domains of action are:<br><br>Housing; Transportation; Outdoor spaces and buildings; Community support and health services; Communication and information;	Interventions not falling into any of these intervention categories or deemed to be AFE programs

	Social participation; Respect and social inclusion; Civic participation and employment<br>2. Programs for age-friendly cities and communities will be classified as local (city or community level), subnational (e.g. regional, provincial, county or state level) and national.	
**Study design**	The study must be a systematic review or EGM, published either in a peer-reviewed journal or a technical report, according to the following definition:<br>• A systematic review is an academic research paper, also called a report, that uses a method called 'evidence synthesis' to look for answers to a pre-defined question.<br>• Systematic review:<br>• The purpose of a systematic review is to sum up the best available research on that specific question. This is done by synthesizing the results of several studies.<br>• A systematic review uses transparent procedures to find, evaluate, and synthesize the results of relevant research. Procedures are explicitly defined in advance, to ensure that the exercise is transparent and can be replicated. This practice is also designed to minimize bias.<br>• Studies included in a review are screened for quality, so that the findings of a large number of studies can be combined. Peer review is a key part of the process; qualified independent researchers review the author's methods and results.<br>• A systematic review must have:<br>o Clear inclusion and exclusion criteria<br>o An explicit search strategy<br>o Systematic coding and analysis of included studies<br>o Systematically report all eligible studies<br>o Meta-analysis (where possible)	- Not a systematic review (e.g., literature reviews) – does not satisfy at least 4/5 criteria (i) Were inclusion/exclusion criteria reported? (ii) Was the search adequate? (iii) Were the included studies synthesized? (iv) Was the quality of the studies included assessed; (v) Are sufficient details about the individual studies includedstudies included presented?<br><br>Any form of primary research study

	• Systematic reviews of quantitative, qualitative, and a mixture of quantitative and qualitative studies will be included. In the case of systematic reviews of qualitative studies, they will be included even if they did not appraise the quality of the included primary studies. We will not pre-define types of qualitative study designs of primary studies which we will include. All of the qualitative study designs included in reviews including qualitative studies relevant domains of actions.<br>• A systematic review may or may not include a meta-analysis. A meta-analysis refers to combining the results of the individual studies in a systematic review to produce an overall statistic.<br><br>EGM<br>• EGMs are systematic and visual presentations of the availability of evidence for a particular topic area. EGMs consolidate and provide a graphical display of areas with strong, weak, or non-existent evidence.<br>• An EGM uses transparent procedures to find, collect, and structure the results of relevant research. Procedures are explicitly defined in advance, to ensure that the exercise is transparent and can be replicated. This practice is also designed to minimize bias.<br>• A typical map is a matrix of intervention categories (rows) and outcome domain (columns). There may be additional filters for study design, location, and population sub-group.<br><br>• An EGM must have:	

	o Clear inclusion and exclusion criteria which are systematically applied<br>o An explicit and systematic search strategy<br>o Systematic coding and analysis of included studies<br>o Systematically report all eligible studies<br>o Systematic and visual presentations of the availability of evidence for a particular topic area.	

Appendix 3 - Coding tool

Categories	Question/Description
**Publication details**	
Publication ID	EPPI Publication ID
Publication Title	Title of publication
Publication date	Year (letter - if more than one study from that author and that year)
Type of evidence	What is the type of evidence?<br>Select the type of evidence:<br>1. Systematic review<br>2. Evidence gap map (EGM)
Language of publication	Language of publication of the systematic review or evidence gap map (EGM).<br>Select the language of publication:<br>1. Arabic<br>2. Chinese<br>3. English<br>4. French<br>5. Russian<br>6. Spanish
Equity	Depending on whether the objectives of the systematic review specifically address the topic (e.g., an SR aimed at looking into equity considerations and health inequalities).<br><br>Subgroup analysis: whether the systematic review reported subgroup analyses or disaggregated findings by any PROGRESS-Plus factor (e.g socio-economic status)<br><br>Equity focused targeting: whether the systematic review explicitly targeted or prioritised populations experiencing inequities (e.g older people in rural communities)<br>Options are:<br>1. Yes<br>2. No
**Context and Population**	
Country	List countries where the interventions included in the systematic review or evidence gap map (EGM) were conducted.

	NB: This information is contained in the primary studies included in the systematic review or evidence gap map (EGM) report.
WHO Region	The World Health Organization (WHO) divides the world into six regions for administrative and operational purposes. Select the region(s) where interventions included in the systematic review or evidence gap map (EGM) were conducted according to the World Bank.<br>Options are:<br>1. African Region (AFRO)<br>2. Region of the Americas (AMRO)<br>3. South-East Asia Region (SEARO)<br>4. European Region (EURO)<br>5. Eastern Mediterranean Region (EMRO)<br>6. Western Pacific Region (WPRO)<br>7. Unspecified<br>NB: This information is contained on the includes primary studies included in the systematic review or evidence gap map (EGM) report.
World Bank Income category	Select the World Bank income classification of the country where the interventions included in the systematic review or evidence gap map (EGM) were conducted according to the World bank.<br>Options are:<br>1. Low-income economies<br>2. Lower-middle income economies<br>3. Upper-middle income economies<br>4. High-income economies<br>5. Unspecified<br>NB: This information is contained in the primary studies included in the systematic review or evidence gap map (EGM) report.
Degree of urbanization	Select the degree of urbanisation for the location of interventions included in the systematic review or evidence gap map (EGM) was conducted according to the sourcebook published by WHO and UN-HABITAT in 2020: Integrating health in urban and territorial planning: a sourcebook. A classification that differentiates between the urban and rural area continuum in three main typologies: city; towns and semi-dense areas; and rural areas (Level 1)

	Options are:<br>1) **City (Densely populated areas):**<br>Consists of contiguous grid cells with a density of at least 1,500 inhabitants per km2. An urban centre has a population of at least 50,000.<br>2) **Towns and semi-dense areas (Intermediate density areas – that includes towns and suburb):**<br>Consists of contiguous grid cells with a density of at least 300 inhabitants per km2 and has a population of at least 5,000 in the cluster (The urban centres are subsets of the corresponding urban clusters).<br><br>3) **Rural areas (Thinly populated areas – that includes Village and Dispersed rural area):**<br>Cells that do not belong to an urban cluster. Most of these will have a density below 300 inhabitants per km2. Some rural cells will have a higher density, but they are not part of cluster with a large enough population size to be classified as an urban cluster.
Age group	The age of the participants/population receiving the intervention as reported in the study or in the primary studies included in the systematic review or evidence gap map (EGM) in years or mean age. Classified into:<br>• ≥50 years<br>• ≥55 years<br>• ≥60 years<br>• ≥65 years<br>• ≥70 years<br>• ≥75years<br>• ≥80 years<br>• >85 years
Sex	The sex of the participants/population receiving the intervention as reported in the study or in the primary studies included in the systematic review or evidence gap map (EGM). Options are:<br>1. Male<br>2. Female<br>3. Not reported/not clear

Disability	Are the interventions targeted or tailored interventions for older people with any disability? Options are:<br>1. Yes<br>2. No
**Intervention characteristics:** The intervention categories are specified for each WHO domain of action and for AFCC programs.	
Intervention type	1. Housing<br>2. Transportation<br>3. Outdoor spaces and buildings<br>4. Community support and health services<br>5. Communication and information<br>6. Social participation<br>7. Respect and social inclusion<br>8. Civic participation and employment<br>9. Age-Friendly Cities and Communities Programs
Levels of implementation	1. National<br>2. Sub-national/regional<br>3. City/metropolitan<br>4. Community<br>5. Care settings<br>6. Inter-individual<br>7. Individual
Instrument for implementation	1. Governance<br>2. Regulations<br>3. Taxes, levies and subsidies<br>4. Infrastructure, technology, and the built environment<br>5. Assessment, monitoring and evaluation<br>6. Information, education and communication<br>7. Other management and control<br>8. Other actions including capacity building
**Outcomes**	
Intermediate outcomes	Short- to mid-term changes in health status or related outcomes. Options are:<br>1. Behavior change<br>2. Social participation<br>3. Risk reduction and prevention<br>4. Removing barriers<br>5. Social Inclusion<br>6. Civic participation and employment<br>7. Falls-related

	8. Food and nutrition<br>9. Elder abuse
Domains of functional ability	Options are:<br><br>1. **Meet older people basic needs**<br>2. **Learn, grow, and make decisions**<br>3. **Be mobile**<br>4. **Build and maintain relationships**<br>5. **5.Contribute**
Domains of intrinsic capacity	Options are:<br>1. Locomotor capacity (physical movement)<br>2. Sensory capacity (such as vision and hearing)<br>3. Vitality (energy and equilibrium)<br>4. Cognitive capacity<br>5. Psychological capacity
Late health outcomes	Options are:<br>1. Mortality<br>2. Life expectancy<br>• Healthy life expectancy<br>• Life expectancy at birth<br>• Life expectancy at 60<br><br>3. Well-being and Quality of life
Economic impact	Options are:<br>1. Service use, quality, satisfaction<br>2. Cost/Cost-effectiveness/benefit
